# Job satisfaction across Europe: An analysis of the heterogeneous temporary workforce in 27 countries

**DOI:** 10.1177/0143831X221088306

**Published:** 2022-04-15

**Authors:** Leandro Iván Canzio, Felix Bühlmann, Jonas Masdonati

**Affiliations:** University of Lausanne, Switzerland

**Keywords:** Contract duration, involuntary temporary work, job satisfaction, temporary work, voluntary temporary work

## Abstract

The consequences of temporary jobs for job satisfaction are not clear. This article examines the effect of two crucial moderators in the association between temporary contracts and job satisfaction: the reason for being a temporary worker and the duration of temporary contracts. Using the ad-hoc module of the 2017 EU Labour Force Survey (EU-LFS), this study examines 27 European countries separately. Results show that involuntary temporary workers (those who wanted a permanent contract but could not find one) tend to be less satisfied than permanent employees. However, voluntary temporary workers (those who prefer temporary over permanent jobs) and temporary workers in apprenticeships or probation periods are generally as satisfied as permanent employees. Shorter contracts frequently exert negative effects on job satisfaction, but only among involuntary temporary workers. Results differ between countries: the differences between temporary and permanent workers are insignificant in Scandinavian countries but large in the post-Socialist states.

## Introduction

Temporary workers^1^ accounted for about 13.6% of the EU-28 workforce in 2019. Their situation in the labour market is a matter of public concern. Besides lacking job security due to the (almost) certain end of their job contract (Ellonen and Nätti, 2015; Parker et al., 2002), fixed-term workers frequently experience poorer job quality than permanent employees: they receive lower wages and economic benefits (Booth et al., 2002; Eurofound, 2015; OECD, 2014, 2015), are offered fewer training opportunities (Forrier and Sels, 2003) and have less autonomy (Goudswaard and Andries, 2002; Wagenaar et al., 2012). Nevertheless, it is not clear whether temporary jobs have negative consequences for job satisfaction (Wilkin, 2013). In the last decades, a fair number of research articles have pointed to three possible outcomes in this regard: temporary workers are more satisfied with their work than permanent employees (Beckmann et al., 2007; De Cuyper and De Witte, 2005, 2007a, 2007b; De Cuyper et al., 2010; Mauno et al., 2005); temporary workers are as satisfied as permanent employees (Allen and Van der Velden, 2001; Bruno et al., 2014; D’Addio et al., 2007; De Cuyper and De Witte, 2006; De Cuyper et al., 2019; De Graaf-Zijl, 2005, 2012; Green and Heywood, 2011; Guest et al., 2006); and temporary workers are less satisfied than permanent employees (Bardasi and Francesconi, 2004; Benavides et al., 2000; Chadi and Hetschko, 2016; Fabra and Camisón, 2009; Green and Tsitsianis, 2005; Kaiser, 2007; Pichler and Wallace, 2009). Job satisfaction is associated with subjective well-being and health (Bowling et al., 2010; Faragher et al., 2005), it predicts job quits (Böckerman and Ilmakunnas, 2009; Clark, 2001; Green, 2010), business outcomes (Harter et al., 2002) and productivity (Böckerman and Ilmakunnas, 2012). Understanding the consequences of temporary employment for job satisfaction is therefore a matter of interest for human resources practitioners, managers, career counsellors, employers, but also policymakers and public health professionals.

This article argues that part of the mixed findings about the effects of temporary employment on job satisfaction can be attributed to the heterogeneity of the temporary workforce between and within countries. More specifically, among the many micro determinants that shape the association between temporary employment and job satisfaction – such as previous work experiences or perceived employability (see De Cuyper et al. [2008] and Dawson [2017] for a review) – we focus on two compositional aspects that have received little or no attention. First, the association between temporary work and job satisfaction depends on workers’ contract preference (Ellingson et al., 1998; Krausz, 2000; Krausz et al., 1995) and the reason for being a temporary worker (De Cuyper and De Witte, 2008). Some workers take temporary jobs because they cannot find permanent employment, while others do so simply because they seek short-term engagement in the labour market or in a certain position. Nonetheless, these findings are limited to a small group of countries, namely the US (Krausz et al., 1995; Maynard et al., 2006), the UK (Guest et al., 2006) and Belgium (De Cuyper and De Witte, 2007b, 2008), and frequently rely on small samples. Secondly, temporary contracts can vary widely in terms of their duration, from just a few days to a few years. Still, the effects of the duration of these temporary contracts on job satisfaction are mostly unexplored. Given that the lack of job security is the main characteristic of fixed-term jobs, contract duration may be a relevant determinant of job satisfaction among temporary workers. These two individual aspects, therefore, might explain some of the cross-national differences on the effects of temporary employment on job satisfaction (De Witte and Näswall, 2003). At the same time, the association between temporary employment and job satisfaction is also affected by institutional factors – for example, cultural aspects or labour market institutions (Clark and Postel-Vinay, 2009; Kristensen and Johansson, 2008). In consequence, the effects of the reason for having a temporary contract, and the duration of the temporary contract on satisfaction, might differ across institutional contexts.

With the aim to tackle job satisfaction combining a micro and macro perspective, this article makes two contributions to the literature. First, we analyse the effects of the reason for being a temporary worker for job satisfaction in 27 European countries at the aggregate European level, and then we explore these effects for each country independently. Second, we evaluate for the first time the effect of contract duration on workers’ satisfaction in Europe, also obtaining estimates for each country. These country-specific analyses constituted an advantage: they unveiled that certain results are common across countries with similar institutional configurations, but also provided results that are particularly relevant for under-researched areas such as the post-Socialist countries.

## Background

### Job satisfaction and temporary work

Two mechanisms explain why temporary workers might be less satisfied than permanent employees. Firstly, fixed-term workers suffer from job insecurity due to the eventual termination of their contract, which negatively affects their overall job satisfaction (Dawson et al., 2017). However, it appears that the job satisfaction of temporary workers is more resistant to the negative effects of job insecurity compared to permanent employees (De Witte and Näswall, 2003; Mauno et al., 2005). Secondly, fixed-term employees experience poorer overall job quality than workers in permanent arrangements. Therefore, even if these workers were immune to job insecurity, they would still have reasons to be less satisfied than permanent employees.

At the same time, there are grounds to justify why temporary workers could be as satisfied or more satisfied than permanent employees. Even if temporary jobs are of poorer quality, temporary workers might experience what some authors call the *honeymoon-hangover effect* (Boswell et al., 2005; Georgellis and Yusuf, 2016). According to this effect, workers’ job satisfaction suddenly increases after they take a new job and progressively returns to pre-transition levels after some time. Thus, because temporary workers have started a new job more recently, it is likely that they will be more satisfied than permanent employees (Chadi and Hetschko, 2016). Similarly, temporary workers are more likely to have recently experienced unemployment, be new entrants in the labour market, and been informally employed. Consequently, a temporary position would be comparatively better than previous situations and may lead to a temporary job satisfaction *bonus*.

In addition to individual-level factors that shape the association between temporary employment on job satisfaction, there are institutional elements that affect this relationship. Cultural features influence the assessment of job satisfaction and the extent to which certain job characteristics such as job security are relevant for job satisfaction (Hauff et al., 2015; Kristensen and Johansson, 2008). Additionally, the effects of temporary jobs on satisfaction depend on economic cycles. For example, during recessions job security becomes more important for job satisfaction (Artz and Kaya, 2014). The same applies to labour market institutions. Regulations on the use of permanent and temporary contracts influence temporary workers’ satisfaction. For instance, temporary workers feel more satisfied with their job security in countries where unemployment benefits are higher (Clark and Postel-Vinay, 2009). Similarly, the legislation that regulates hiring and firing procedures for permanent and temporary workers influences how insecure temporary workers feel compared to permanent employees (Balz, 2017). In consequence, due to the influence of multiple institutional factors, the effects of temporary employment on job satisfaction cannot be deemed as constant across different contexts.

### Voluntary and involuntary temporary work

Given the lower wages and lack of security affecting temporary workers, it is not surprising that most find their engagement in the labour market to be suboptimal. According to Eurostat, in 2017 around 53% of temporary workers in the EU and EFTA countries claimed to have a fixed-term arrangement because they could not find a permanent job (see Table 1). These workers are what some researchers label as ‘involuntary temporary workers’ (e.g. Feldman et al., 1995; Krausz et al., 1995) and represent more than 80% of the temporary workforce in Cyprus, Spain, Portugal and Romania, but less than 10% in Iceland or Austria.

**Table 1.** Temporary workers by reason for having a temporary job, as a percentage of the temporary workforce aged 15–64 in Europe (2017).

	Could not find a permanent job	Did not want a permanent job	In education or training	Probationary period
EU+EFTA	53.1	12.4	15.2	8.0
Belgium	75.8	19.5	4.7	0.0
Bulgaria	68.3	12.4	0.0 (*)	16.2
Czechia	77.1	21.5	1.0	0.0
Denmark	39.6	27.2	28.5	4.6
Germany	15.1	3.2	39.6	13.5
Estonia	12.0 (*)	11.9 (*)	0.0 (*)	46.8
Ireland	39.1	21.8	8.3	4.7
Greece	72.5	3.5	9.2	6.1
Spain	85.2	3.1	4.6	1.0
France	54.2	21.9	13.3	2.4
Croatia	86.0	5.1	6.4	2.3 (*)
Italy	72.4	2.3	16.4	8.5
Cyprus	91.9	2.3 (*)	3.5	2.3 (*)
Latvia	56.3	20.1	0.0 (*)	18.1
Lithuania	56.6	11.7 (*)	13.8 (*)	17.9 (*)
Luxembourg	57.1	6.7	5.4	15.4
Hungary	77.5	9.1	2.1	11.3
Malta	46.5	19.8	7.6	26.1
Netherlands	31.1	12.3	2.6	26.9
Austria	9.1	35.5	43.1	12.2
Poland	58.8	19.8	9.0	12.4
Portugal	82.4	5.4	5.1	7.1
Romania	84.2	0.0	0.0	0.0
Slovenia	53.3	35.5	2.7 (*)	8.5
Slovakia	77.1	17.6	1.7	0.0
Finland	70.3	22.9	4.2	1.9
Sweden	51.2	32.6	1.1	12.6
United Kingdom	28.7	25.0	9.0	3.2
Iceland	5.8	49.2	3.6	8.0
Norway	50.1	22.5	10.4	0.0
Switzerland	11.3	5.5	56.3	2.8

Note: Some rows do not add up to 100% due to the missing answers. (*) Values with low reliability.

Source: European Labour Force Survey, 2017.

For others, a temporary job might be an instrument, a stepping-stone towards a permanent position or the path to achieve a set of skills that could open new labour market opportunities (Booth et al., 2002; De Jong et al., 2009; Van den Berg et al., 2002). This is the case for 8% of European temporary workers under probation periods and 15.2% who are doing internships or apprenticeships. In Switzerland, Germany and Austria, these workers account for more than half of fixed-term contracts, whereas in most Eastern Europe countries they represent a very small share of the workforce. For practical reasons, we follow previous studies in the field (e.g. De Cuyper and De Witte, 2008) and refer to temporary workers in probation periods, apprentices and trainees as ‘instrumental temporary workers’.

Another 12.4% of temporary workers are employees who claim to have a fixed-term contract simply because they did not want to have a permanent one. They might decide to have an intermittent engagement in the labour market due to their participation in other activities (Casey and Alach, 2004). Tan and Tan (2002) observed that some workers actively seek a temporary position for family and economic reasons (e.g. greater flexibility), self-improvement (e.g. gaining experience in different organizations) or simply because of a personal preference (e.g. a desire for less office politics). This group represents more than one-third of the temporary workforce of Slovenia, Austria and Iceland. We refer to them as ‘voluntary temporary workers’.

The reason why workers have a temporary position has been considered as a crucial moderator in the association between contract type and job satisfaction. This association has also been conceptualized as *work status congruence* or *contract mismatch*, or sometimes encompassed by the more general psychological concept of *volition*. Numerous scholars argue that being voluntarily engaged in a temporary job has positive impacts on job satisfaction (Connelly and Gallagher, 2004; Ellingson et al., 1998; Feldman et al., 1995; Guest, 2004; Krausz et al., 1995; Tan and Tan, 2002; Westover, 2012).

Nevertheless, studies on the association between the preference for temporary jobs and job satisfaction are scant and frequently rely on small and scarcely diverse samples in a limited number of countries. Some researchers have observed that workers who are voluntarily engaged in a temporary position are sometimes more satisfied than temporary workers who prefer a permanent job (Ellingson et al., 1998; Guest et al., 2006) or even permanent employees (Krausz et al., 1995), while Maynard et al. (2006) observed different effects for different facets of job satisfaction. By contrast, De Cuyper and De Witte (2007b) found no association between contract preference and job satisfaction, and De Cuyper and De Witte (2008) reported that ‘free choice’ temporary workers were less satisfied than ‘forced choice’ ones and permanent employees.

In line with most of the theoretical arguments and part of the evidence, three hypotheses are tested:

*H1*: Involuntary temporary workers are less satisfied than permanent employees.
*H2a*: Instrumental temporary workers are equally or more satisfied than permanent employees.
*H2b*: Voluntary temporary workers are equally or more satisfied than permanent employees.

### Contract duration

The duration of temporary contracts is a significant source of heterogeneity among the fixed-term workforce. Contract duration might be related to different perceptions of job security, but its effects on job satisfaction have received little research attention. As shown in Table 2, these differences exist between and within countries in Europe. For example, in Germany, Austria and Switzerland, close to 40% of temporary workers have contracts of more than 2 years in duration, unlike the Baltic countries, where temporary contracts lasting more than 1 year are negligible. In Estonia, Lithuania, Latvia, Croatia and Belgium, more than one-third of temporary workers have contracts with a maximum duration of 3 months, while in Germany, Cyprus and the Czech Republic (Czechia) fewer than 4% of contracts are of this type.^2^

**Table 2.** Temporary workers by contract duration, as a percentage of the temporary workforce aged 15–64 in Europe (2017).

	Up to 3 months	From 4 to 6 months	From 7 to 12 months	From 13 to 24 months	More than 2 years
EU+EFTA	16.1	14.9	25.2	10.7	15.9
Belgium	37.3	15.8	29.3	6.7	11.0
Bulgaria	17.8	39.0	23.2	2.5 (*)	0.0 (*)
Czechia	3.9 (*)	10.1	42.4	25.8	17.6
Denmark	11.3	12.8	20.7	20.6	34.6
Germany	3.3	11.5	28.7	15.4	38.0
Estonia	34.5 (*)	32.2 (*)	9.8 (*)	0.0 (*)	0.0 (*)
Ireland	13.3	9.8	19.4	8.8	12.4
Greece	12.7	28.8	39.5	7.0	12.0
Spain	17.7	15.3	14.8	1.6	4.6
France	30.6	14.7	21.6	15.6	8.3
Croatia	34.7	25.4	20.4	2.9	14.5
Italy	22.9	26.3	29.3	3.1	11.0
Cyprus	3.6 (*)	17.1	41.4	12.0 (*)	24.7 (*)
Latvia	38.3 (*)	25.6	14.1	0.0 (*)	7.9 (*)
Lithuania	42.1 (*)	27.7	17.9	0.0 (*)	0.0 (*)
Luxembourg	16.4	12.8	25.1	16.9	26.9
Hungary	24.6	17.9	50.3	4.2	2.9
Malta	8.9 (*)	24.1	33.9	11.6 (*)	17.0 (*)
Netherlands	5.1	4.4	27.7	3.5	2.0
Austria	11.2	14.0	25.7	9.2	39.7
Poland	15.1	11.7	31.9	20.9	20.5
Portugal	13.1	27.5	35.9	3.0	5.2
Romania	14.5 (*)	28.2	45.3	0.0 (*)	0.0 (*)
Slovenia	28.8	20.2	33.6	9.4	8.2 (*)
Slovakia	19.4	28.5	36.5	10.0	2.8 (*)
Finland	26.8	24.0	28.7	11.1	7.4
Sweden	21.7	15.5	14.6	12.5	11.6
United Kingdom	6.5	6.4	12.1	12.3	10.4
Iceland	31.8 (*)	19.7	29.5	0.0 (*)	6.4 (*)
Norway	5.4 (*)	5.3	13.0	11.0 (*)	17.8
Switzerland	12.1	10.7	23.2	10.2	43.7

Note: Some rows do not add up to 100% due to the missing answers. (*) Values with low reliability.

Source: European Labour Force Survey, 2017.

Although the effects of contract duration on temporary workers’ job satisfaction are not well known, some studies have focused on temporary agency workers, who normally have shorter contracts. The results more consistently point to the fact that temporary workers are less satisfied than permanent employees (Aletraris, 2010; Buddelmeyer et al., 2015; De Graaf-Zijl, 2005, 2012; Green and Heywood, 2011; Green et al., 2010; Jahn, 2015). However, it remains unanswered whether temporary agency workers are less satisfied because they have shorter contracts and experience more job insecurity, or because they are exposed to poorer job quality in general (De Graaf-Zijl, 2012; Green et al., 2010).

Following the previous evidence and given that shorter temporary contracts offer less job security, it is expected that temporary workers with short contracts will experience larger differences in job satisfaction with respect to permanent employees than temporary workers with longer contracts. However, voluntary temporary workers are not looking for job security, and for instrumental temporary workers, job security is probably not yet their main priority. Short contracts, then, should have a negative effect on the job satisfaction of those who seek job security: temporary workers who want a permanent job. Therefore:

*H3*: Compared to permanent employees, involuntary temporary workers are less satisfied with their jobs when their temporary contracts are short rather than long.

On the other hand:

*H4a*: Differences in job satisfaction between permanent and instrumental temporary workers do not depend on the duration of the temporary contract.
*H4b*: Differences in job satisfaction between permanent and voluntary temporary workers do not depend on the duration of the temporary contract.

## Data and methods

The study data were retrieved from the ad-hoc module of the 2017 European Labour Force Survey (EU-LFS), which contains information for 27 European countries: Austria, Belgium, Bulgaria, Cyprus, the Czech Republic, Germany, Denmark, Estonia, Spain, Finland, France, Greece, Hungary, Ireland, Italy, Lithuania, Luxembourg, Malta, the Netherlands, Norway, Poland, Portugal, Romania, Sweden, Switzerland, Slovak Republic and the UK. This is the only dataset that provides information about job satisfaction, the duration of temporary contracts, and the reason for being a temporary worker across Europe. The study population is employees aged 15–64.^3^ After discarding proxy interviews, observations with missing values for key variables, workers who had a secondary job in which they worked more than 10 hours per week, and employees who worked and resided in different countries, the final sample consisted of 378,112 observations. Among them, 46,172 were temporary workers and the rest permanent employees, although these numbers changed slightly in some analyses (further details about the sample are provided in the supplementary materials). The hypotheses are tested first on the aggregate European sample. Then we explore these associations on each country independently, obtaining specific results for each national context. This presents two advantages compared to an aggregate analysis of Europe. On the one hand, it allows detecting whether associations differ between territories and institutional configurations. On the other hand, country-level analyses provide detailed information for areas that are frequently under-researched, such as the post-Socialist countries.

The independent variable, *reason for having a temporary job*,^4^ covered four categories: (1) ‘it is a contract covering a period of training (apprentices, trainees, research assistants, etc.)’;^5^ (2) ‘person could not find a permanent job’; (3) ‘person did not want a permanent job’; and (4) ‘it is a contract for a probationary period’. The first and fourth categories were identified as ‘*instrumental* temporary workers’. These workers accept a temporary job to achieve something else: either a permanent position (probation periods) or a certain qualification and skills (apprenticeships and internships).^6^ The second category was identified as ‘*involuntary* temporary work’ and comprised workers who were seeking a permanent position but could not find one. Finally, ‘not wanting a permanent job’ was labelled as ‘*voluntary* temporary work’.

The second independent variable, *contract duration*, was codified in three different categories to capture non-linearities: up to 6 months, 7–12 months, and more than 1 year. This categorization attempts to pool a relevant number of observations for each category and country.

The dependent variable, *job satisfaction*, was assessed with the question ‘To what extent are you satisfied with your current job?’, and four possible responses: ‘satisfied to a large extent’, ‘satisfied to some extent’, ‘satisfied to a small extent’ and ‘not satisfied at all’. The responses were recorded as if job satisfaction were a continuous variable, assigning the values 100, 66.66, 33.33 and 0 to each respective answer. This facilitated the interpretation of the results by reducing the number of coefficients shown to the reader and allowed for the use of linear regression with heteroscedasticity-robust indicators. Although linear regression is not suitable for analysing ordinal outcomes, other techniques entailed similar issues. Ordinal regression implied the violation of the parallel lines assumption, logistic regression caused a loss of information and differing results depending on how the dichotomization was done, and multinomial logistic regression provided less efficient results. Nonetheless, multinomial regression models are provided as a robustness check. The results (in the online supplementary materials) are virtually the same.

The control variables were age, gender, nationality, education, working time, supervisory role, occupation and tenure (further details can be found in the online supplementary materials). To mitigate concerns about collinearity with the independent variable, all the models were tested with and without tenure. The results were essentially the same (results available upon request). Tenure was not included in the models which studied the effects of different contract durations.

Income, number of dependent children and number of unemployed adults in the household were confounders, but they were not included in the main models presented in the article. In some countries, these variables were not available or had many missing values. Therefore, when possible, the analyses were repeated including these controls only for the analysis of the reason for being a temporary worker, but not for contract duration (because of the few observations). The relationships (results in the online supplementary materials) remain mostly unchanged, both in their coefficients and significance. Contrary to other studies, the analyses did not control for agency work. This is because legal regulations differ between countries and there are very few agency workers in some countries. The associations remain equal once temporary agency workers are eliminated from the sample (results available upon request). The models without agency workers were not tested in the analysis of contract duration due to the low number of observations.

In the first step of the analysis, we performed linear regression models to quantify the job satisfaction gap between permanent employees and each of the three categories of temporary workers (involuntary, instrumental and voluntary). The first model was performed for the total sample of countries (this is, including country dummies), and the next models were performed on each country separately. The second step consisted in the analysis of contract duration, first for the total sample, and then for each country separately. In these models the three categories of temporary workers were independently compared with permanent employees and the association between contract duration and job satisfaction was measured. In the country-specific analyses some coefficients had to be suppressed and some countries were fully discarded from the analyses due to the low number of observations for certain categories of the independent variables. This especially occurred in the analyses concerning contract duration due to the high number of missing values for this variable. These suppressions were done attending to the Eurostat guidelines, which require minimum number of observations per category for each country.^7^ This risk of sample bias and the impossibility of assuming causality (due to the cross-sectional sample design) constitute the two main weaknesses of the analysis.

## Results

### Reason for having a temporary job and job satisfaction

**Figure 1.**
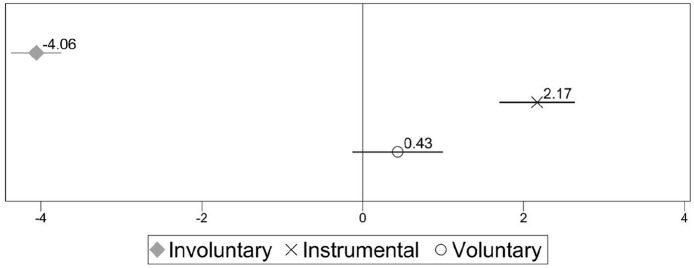
Difference in job satisfaction between permanent (ref.) and different kinds of temporary workers for the overall sample. Estimates from linear regression models (C.I. 95%). Note: Full models are available in Table A1 in supplementary materials.

Each of the coefficients with confidence intervals reported in Figure 1 represents the gap in job satisfaction between permanent employees and each category of temporary workers (involuntary, instrumental and voluntary) for the total sample of countries, including control variables.^8^

The figure shows that in Europe involuntary temporary workers are on average less satisfied than permanent employees, with a significant difference of 4.06 points on the job satisfaction scale (going from 0 to 100). Instrumental temporary workers are in the opposite situation, as they are significantly more satisfied than permanent employees (*B* = 2.17). The voluntary temporary workers, instead, are just as satisfied as permanent employees (*B* = 0.43). The model (full results available in Table A1 in supplementary materials) also shows that workers aged 15–24 tend to be more satisfied than workers aged 35–44 (*B* = 1.483). No significant association is observed for gender, education and marginal workers (those working fewer than 15 hours per week), while part-time workers (those working between 15 and 30 hours per week) are significantly less satisfied (*B* = −0.081) than full-timers. Migrant workers also report lower job satisfaction than native workers, both if they are European (*B* = −1.664) and non-European (*B* = −1.244). Unsurprisingly, supervisors are significantly more satisfied with their jobs (*B* = 1.685), as well as workers with higher occupational status, compared to elementary workers. For example, while managers present a difference of 11.537 points, for service and sales workers it is of 4.781, and for plant and machine operators and assemblers it is of 2.762 points. Tenure also presents a positive association with job satisfaction (*B* = 0.003), which is not surprising, as dissatisfied workers are more likely to quit.

**Figure 2.**
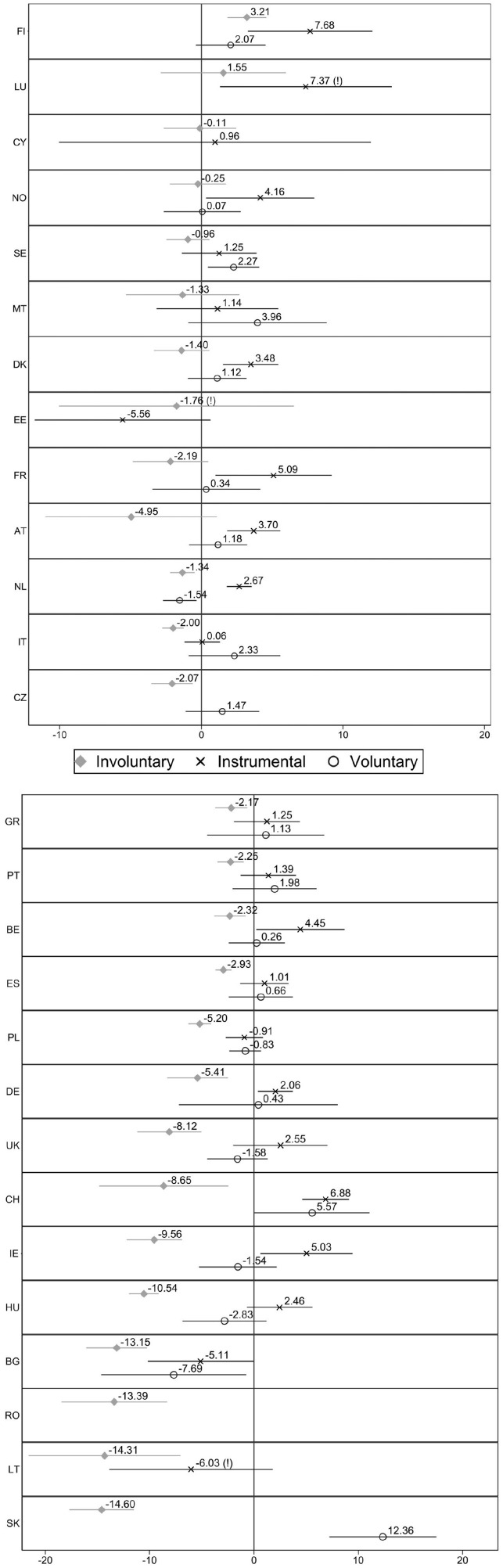
Difference in job satisfaction between permanent (ref.) and different kinds of temporary workers, by country. Estimates from linear regression models (C.I. 95%). Note: Full models are available in Table A2 in supplementary materials. Some coefficients or countries are not reported because of few observations. (!) Indicates that the coefficient is unreliable because of few observations, according to Eurostat guidelines.

In Figure 2 we present the country-specific results of these associations. It shows the gap in job satisfaction between permanent employees and each category of temporary worker for each country. To facilitate the interpretation of the results, the countries are sorted by the size of the coefficient of involuntary temporary workers (those with non-significant associations rank first).

Involuntary temporary workers are as satisfied as permanent employees in 9 out of 27 countries, and more satisfied in one country (Finland, *B* = 3.21). This is the case in the Scandinavian countries (Norway, Sweden and Denmark), as well as in Estonia, Luxembourg, France, Austria, and the two Mediterranean islands of Malta and Cyprus. In the other 17 countries, involuntary temporary workers report less job satisfaction compared to permanent employees, but the cross-national variation is high. The first identifiable cluster is composed of the remaining Southern European countries (Italy, Greece, Portugal and Spain), the Netherlands, Belgium and the Czech Republic. Their gaps are significant but small: below 3 points. This cluster is followed by Germany and Poland, which present negative coefficients of 5 points, and by the UK, Switzerland and Ireland, where the coefficients range from 8 to 9 points. The largest gaps – larger than 10 negative points – are observed in most of the post-Socialist countries (Hungary, Bulgaria, Romania, Lithuania and Slovakia), with Slovakia showing the largest difference (*B* = −14.60).

Instrumental temporary workers exhibit less variation between countries than involuntary temporary workers. In 11 out of 24 countries they are significantly more satisfied than permanent employees, particularly in some Scandinavian countries (Finland, Norway and Denmark) and in most of the Western European ones (Luxembourg, France, Austria, the Netherlands, Belgium, Germany, Switzerland and Ireland). In 12 out of 24 countries instrumental temporary workers were as satisfied as permanent employees. This is the case of Sweden, the UK, the Southern European countries, and most of the post-Socialist ones (some coefficients are not reported because of insufficient observations). Finland, Luxembourg and Switzerland showed the largest significant positive coefficients (about 7 points), while Germany and the Netherlands showed the lowest (about 2 points). Bulgaria stands out as the only country where instrumental temporary workers are less satisfied than permanent employees (*B* = −5.11, *p* = 0.045).

Lastly, voluntary temporary workers report the same satisfaction as permanent employees in all but five countries: in Sweden (*B* = 2.27), Switzerland (*B* = 5.57, *p* = 0.048) and Slovakia (*B* = 12.36), where they are more satisfied than permanent employees, and in Bulgaria (*B* = −7.69) and the Netherlands (*B* = −1.54), where the associations are negative.

Given these results, Hypothesis 1 can be confirmed: involuntary temporary workers are, on average, less satisfied than permanent employees. However, this job satisfaction difference is not the same across countries, as it is observed in 17 out of the 27 national samples. The gap does not exist in the Scandinavian countries but is very frequent and large in the post-Socialist ones. The results for Western Europe are mixed, but the difference appears to be larger in the most liberal economies (i.e. Ireland, the UK and Switzerland). Hypothesis 2a is also supported by the results. On average, instrumental temporary workers are more satisfied than permanent employees in Europe. This especially occurs in the Scandinavian and Western European regions, while in Southern Europe and most post-Socialist countries they present the same job satisfaction as permanent employees. Hypothesis 2b is confirmed too: except in a few countries, voluntary temporary workers are as satisfied as permanent employees.

### Duration of temporary contracts and job satisfaction

**Figure 3.**
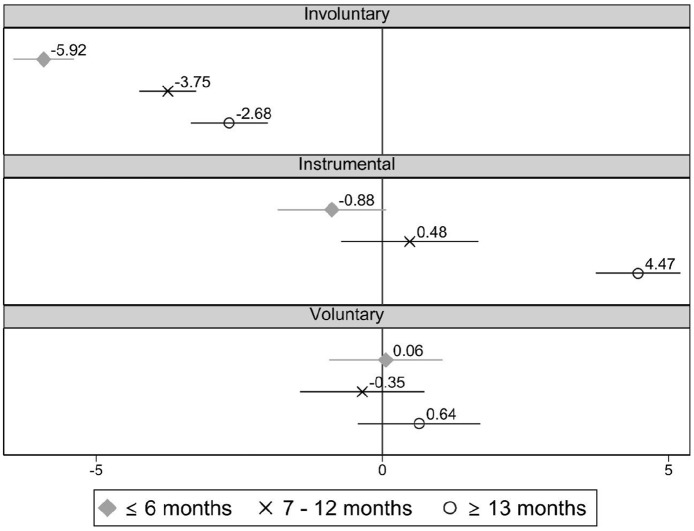
Difference in job satisfaction between permanent (ref.) and different kinds of temporary workers with different contract durations for the overall sample. Estimates from linear regression models (C.I. 95%). Note: Full models are available in Table A3 in supplementary materials.

In Figure 3, each coefficient and confidence interval represents the gap in satisfaction between permanent and involuntary temporary workers depending on the duration of their contract for the aggregate sample of countries, controls being included.

Involuntary temporary workers tend to present larger job satisfaction differences with respect to permanent workers when their temporary contracts are shorter. These differences are of 5.92 points for those with contracts lasting 6 months or less, but only of 2.68 points when their contracts last 13 months or more. Results in the supplementary materials (Table A3) also show that the three categories of contract duration are also significantly different from each other, being those with short(long) temporary contracts the least(more) satisfied among the temporary employees. Instrumental temporary workers tend to present the same job satisfaction as permanent employees, except if their temporary contracts are long, when they are significantly more satisfied (*B* = 4.47). For the voluntary temporary workers, results show that they are as satisfied as permanent employees, regardless of their contract duration.

**Figure 4.**
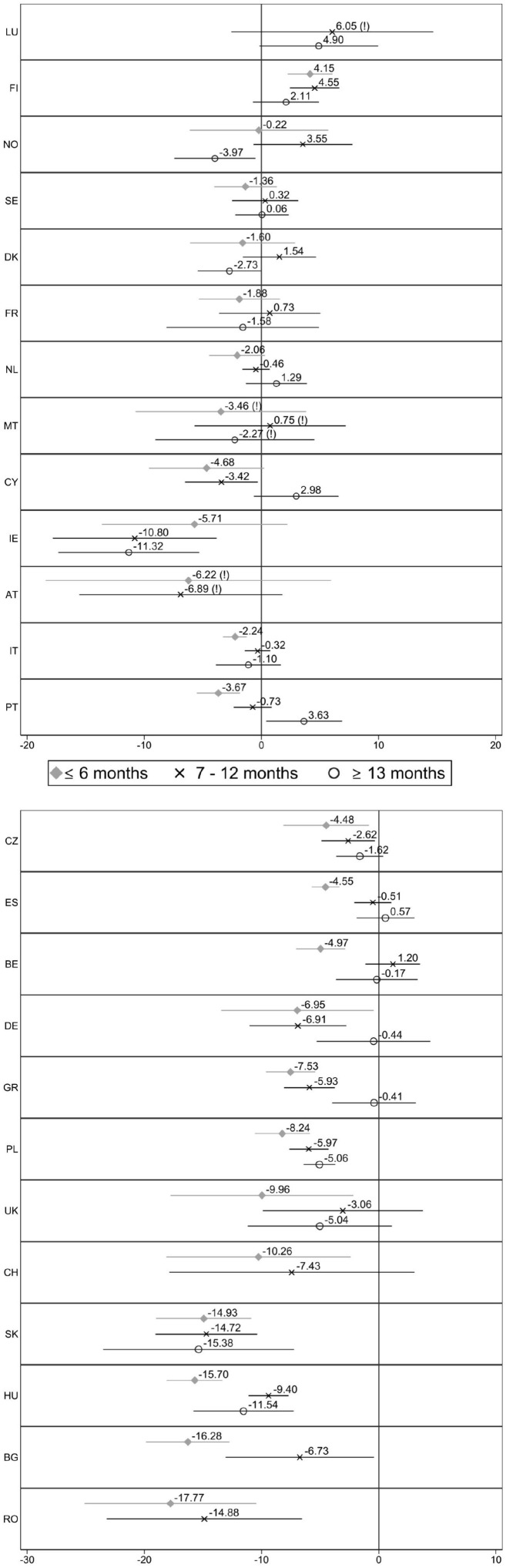
Difference in job satisfaction between permanent (ref.) and involuntary temporary workers with different contract durations, by country. Estimates from linear regression models (C.I. 95%). Note: Full models are available in Table A4 in supplementary materials. Some coefficients or countries are not reported because of few observations. (!) Indicates that the coefficient is unreliable because of few observations, according to Eurostat guidelines.

Figure 4 shows the country-specific results of the job satisfaction gap between involuntary temporary workers and permanent employees by contract duration. Countries are sorted by the size of the coefficient involuntary temporary workers with short contracts present (with those at the top showing non-significant associations). Several coefficients are not reported because of the small number of observations.

Involuntary temporary workers with short contracts (6 months or less) are significantly less satisfied than permanent employees in 14 out of 24 countries. However, when their temporary contracts are long (more than 1 year in length), they are significantly less satisfied than permanent employees in only 6 out of 21 countries. In 18 out of 24 countries, involuntary temporary workers with short contracts are, on average, less satisfied than temporary workers with longer contracts. Further analyses (results not shown) reveal that these differences are statistically significant in 9 out of 24 countries (Cyprus, Italy, Portugal, Spain, Belgium, Greece, Poland, Hungary and Bulgaria). Workers with short temporary contracts are not found to be significantly more satisfied than workers with longer contracts in any of the countries. Figure 2 previously showed that there was no gap in job satisfaction between involuntary temporary workers and permanent employees in the Scandinavian countries and Malta, Cyprus, Austria, France and Luxembourg. Now, Figure 4 suggests that involuntary temporary workers in these countries are as satisfied as permanent employees – and even more satisfied than them in Finland (*B* = 4.15) – even when their contracts provide job security for only 6 months or less. Surprisingly, temporary workers in Denmark and Norway are less satisfied than permanent employees, but only when their contracts last more than 1 year (*B* = −2.73, *p* = 0.047, and *B* = −3.97, respectively). Ireland’s situation is similar: employees with short contracts are as satisfied as permanent ones, but those with longer contracts present significant negative coefficients. The gaps in satisfaction for workers with short contracts range from 2 to 5 points in Italy, Portugal, the Czech Republic, Spain and Belgium and widen (6–11 points) in Germany, Greece, Poland, the UK and Switzerland, with the post-Socialist countries showing the largest gaps. Specifically, involuntary temporary workers with short contracts in Slovakia, Hungary, Bulgaria and Romania report a 14–18 point lower job satisfaction than permanent employees. Although some coefficients are missing, the results suggest that involuntary temporary workers are significantly less satisfied than permanent employees in this group of countries, regardless of the contract duration.

**Figure 5.**
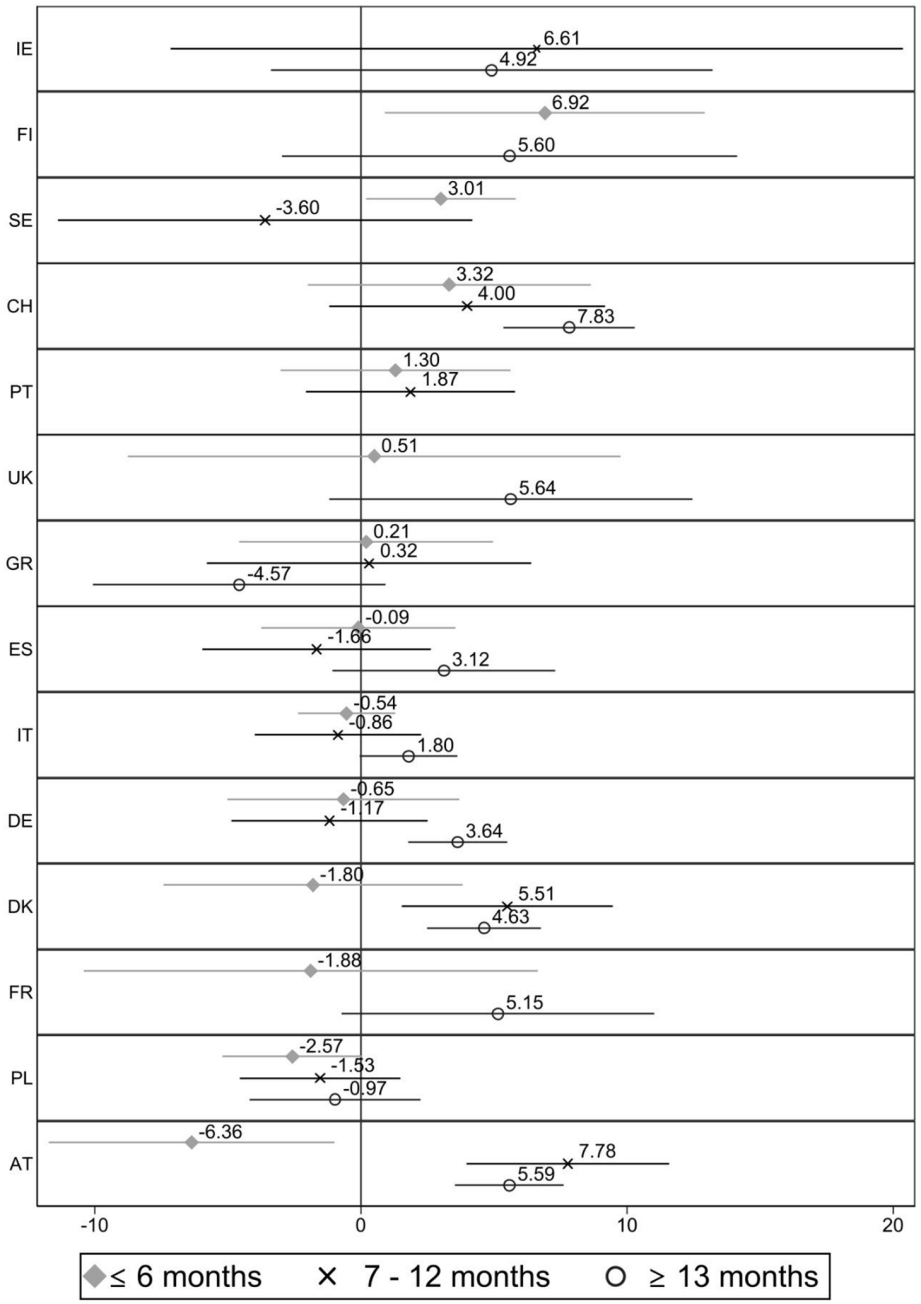
Difference in job satisfaction between permanent (ref.) and instrumental temporary workers with different contract durations, by country. Estimates from linear regression models (C.I. 95%). Note: Full models are available in Table A5 in supplementary materials. Some coefficients or countries are not reported because of few observations. (!) Indicates that the coefficient is unreliable because of few observations, according to Eurostat guidelines.

**Figure 6.**
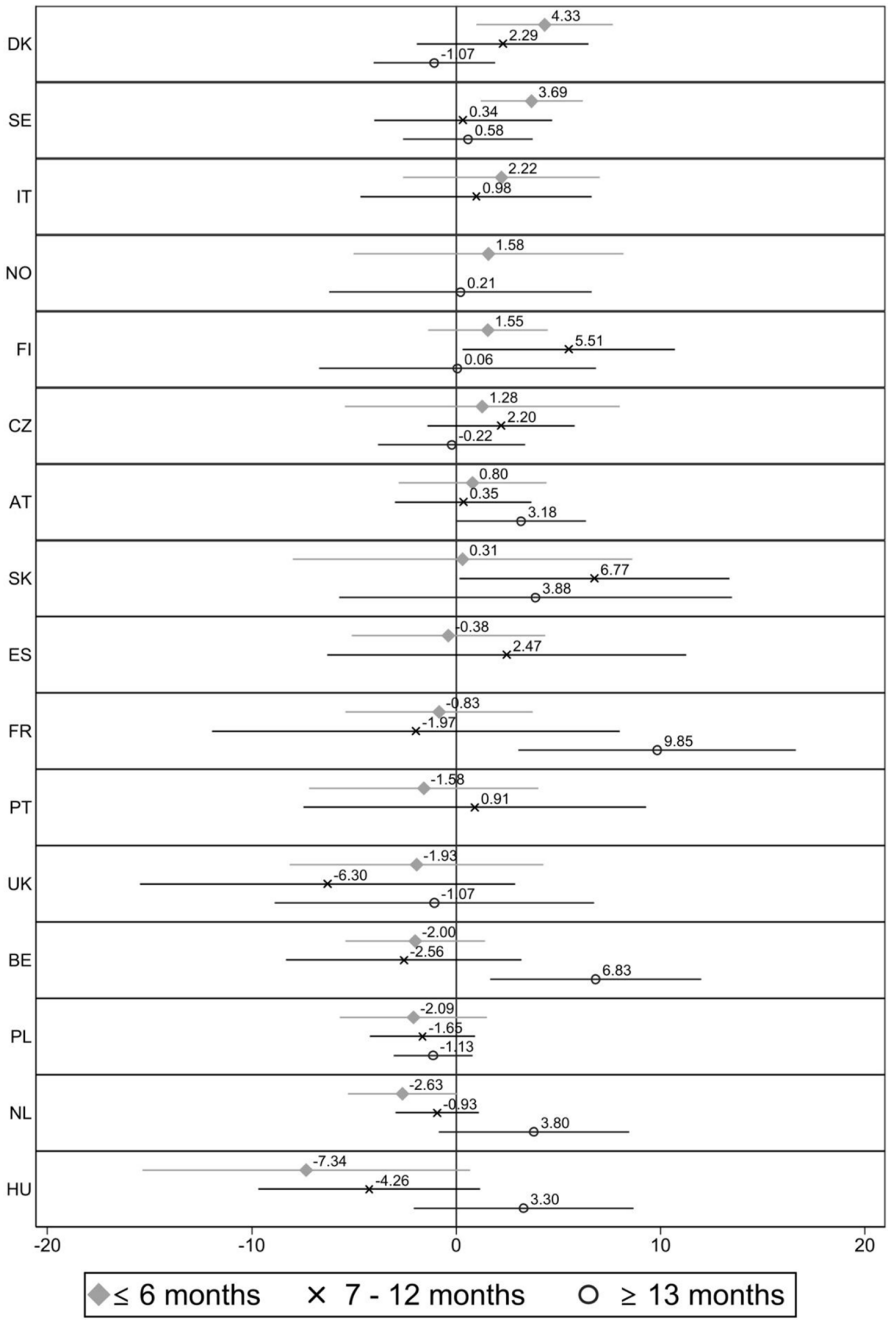
Difference in job satisfaction between permanent (ref.) and voluntary temporary workers with different contract durations, by country. Estimates from linear regression models (C.I. 95%). Note: Full models are available in Table A6 in supplementary materials. Some coefficients or countries are not reported because of few observations. (!) Indicates that the coefficient is unreliable because of few observations, according to Eurostat guidelines.

Figure 5 displays gaps in job satisfaction between permanent and instrumental temporary workers of different duration. Figure 6 shows the same information but for permanent and voluntary temporary workers. Only half of the countries are reported in each figure due to the small number of observations in some of them.

For instrumental temporary workers, contract duration does not seem to affect job satisfaction. The difference in job satisfaction with respect to permanent workers is usually similar between the three contract lengths. In some cases, instrumental temporary workers report more satisfaction when the contracts are longer, and in other cases the opposite effect occurs. These temporary workers are significantly less satisfied than permanent employees only in Austria when they have contracts lasting 6 months or less (*B* = −6.36). However, those in Finland and Sweden are also more satisfied when their contracts are short (*B* = 6.92 and *B* = 3.01, respectively). When their contracts are longer than 1 year, instrumental temporary workers report more satisfaction than permanent employees in Switzerland, Germany, Denmark and Austria, with coefficients ranging from 3 to 7 points.

The picture is similar for voluntary temporary workers (Figure 6). Again, the mean gap in job satisfaction with respect to permanent employees across countries does not seem to be systematically lower when they have short rather than long contracts. Voluntary temporary workers with short contracts were not found to be less satisfied in any of the 16 countries observed, while they are significantly more satisfied in Denmark and Sweden (*B* = 4.33 and *B* = 3.69, respectively). By contrast, in three countries (France, Austria and Belgium) they are significantly more satisfied when their contracts last more than 1 year, with coefficients ranging from 3 to 9 points. The same applies to Finland (*B* = 5.51) and Slovakia (6.77) for workers with contracts of 7–12 months in duration.

According to the results, Hypothesis 3 is only partially confirmed: analysis for the overall sample showed that, compared to permanent employees, involuntary temporary workers report less job satisfaction when their temporary contracts are short. However, the evidence is less consistent than in previous hypotheses. Contract duration appears to be positively related to job satisfaction among involuntary temporary workers, but only in some Southern and Western European countries. Conversely, involuntary temporary workers in the Scandinavian countries are generally as satisfied as permanent employees, regardless of the contract duration. In the post-Socialist countries, these workers were generally *less* satisfied than permanent employees, with no frequent differences found for contract duration. Hypotheses 4a and 4b are both confirmed. The duration of temporary contracts does not affect the job satisfaction gap between permanent and instrumental temporary workers, nor between permanent and voluntary temporary workers. Contrary to findings for involuntary temporary workers, instrumental and voluntary temporary workers are not less satisfied when their contracts are short. Nonetheless, results suggest that instrumental and voluntary temporary workers in some countries might experience a job satisfaction *bonus* compared to permanent ones when their contracts are long.

## Conclusions

This article investigates under which conditions temporary workers are more, equally or less satisfied than permanent employees. This is addressed by exploring the effects of the reason for having a temporary contract and the duration of these temporary contracts on job satisfaction across 27 European countries.

The results show that involuntary temporary workers are generally less satisfied than permanent employees, albeit with significant variations depending on the context. In the Scandinavian countries there is no job satisfaction gap between permanent and involuntary temporary workers, in Western and Southern Europe the gaps were significant but small, while in most of the post-Socialist countries the differences were large. The duration of temporary contracts also affects the job satisfaction of involuntary temporary workers. When temporary contracts are short, these workers tend to be less satisfied than permanent employees, and when they are long, the differences in job satisfaction are smaller. This applies to most of the Southern and Western European countries. By contrast, involuntary temporary workers in the Scandinavian countries are generally as satisfied as permanent employees, regardless of the contract duration. In most of the post-Socialist countries these workers show substantial differences in job satisfaction with respect to permanent employees, even when their temporary contracts are long.

The fact that the Scandinavian and the post-Socialist countries arise as two clearly distinct clusters suggests that structural factors might affect the association between temporary employment and job satisfaction. This could be attributed to the generous unemployment benefits of the Scandinavian countries, which could mitigate the negative consequences of job insecurity for job satisfaction. Conversely, in the post-Socialist countries, where social protection is generally low, the effects of job insecurity on job satisfaction would be more pronounced. However, the countries in these two regions share cultural elements and recent economic trajectories that might also determine how certain job characteristics impact on workers’ satisfaction. For example, during the Soviet period job insecurity was technically not an individual concern as the state was supposed to provide stable jobs for all workers. Therefore, it might be possible that insecure jobs in the post-Socialist countries have a deeper negative impact on job satisfaction.

The analyses also show that the voluntary temporary and instrumental temporary workers (i.e. those in probation periods, interns and trainees) are, in general, as satisfied as permanent employees. However, instrumental temporary workers appear to be even more satisfied than permanent employees if their contracts are long, especially in Western Europe. This particular result could be explained by the institutionalization of vocational education and training systems in this region, where public and private support for and involvement in these programmes is strong (Busemeyer and Schlicht-Schmälzle, 2014).

These findings have methodological implications for future research. They illustrate that the temporary workforce is deeply heterogeneous within and between countries, as well as the effects of temporary contracts on job satisfaction. The reasons for accepting a temporary contract and the duration of the temporary contract seem to determine workers’ well-being. Not accounting for these factors can easily lead to spuriousness as is clearly reflected in countries like Ireland, Switzerland or Germany. In these countries, apprentices and interns drive the average job satisfaction of temporary workers upwards, while involuntary temporary workers do the opposite. The same applies to the contract duration in most of Southern Europe. Although most temporary workers are involuntary, those with long temporary contracts are as satisfied as permanent employees. When assessing well-being at work, precariousness, or job insecurity, researchers might consider focusing on specific profiles of temporary workers, or at least accounting for these compositional differences.

For managers, human resources practitioners and policymakers, our results might preliminarily lead to two main implications. First, the fact that longer temporary contracts tend to mitigate the negative impacts on job satisfaction of involuntary temporary workers suggests that long contracts should be promoted to enhance workers’ job satisfaction. This speaks against offering consecutive short temporary contracts, a practice that some employers seem to follow to avoid firing costs, and to obtain more productivity from workers at risk of job loss (Engellandt and Riphahn, 2005; Güell and Petrongolo, 2007; Polavieja, 2003). Legislation also imposes limitations on the duration of the temporary contracts (Tomas, n.d.; Wexels-Riser, n.d.). Whereas limiting the number of consecutive temporary contracts might protect workers from abusive situations, limiting the duration of temporary contracts might negatively impact their well-being. Second, the voluntariness dimension of temporary contracts, and particularly the fact that involuntary temporary workers tend to be less satisfied than permanent ones, should also be considered. While offering temporary contracts to workers who pursue temporary positions seems positive for their job satisfaction, initiatives should be implemented to foster the access to permanent contracts to workers who do not aspire to temporary jobs.

Finally, these results open new questions that need to be explored. This article only presented associations, but longitudinal designs could better identify causal relationships. Such designs could also help to discern whether the negative effect of short temporary contracts on job satisfaction is partially offset by the honeymoon-hangover effect. Indeed, in the absence of this effect, workers with short contracts might present deeper differences in job satisfaction compared to permanent employees. At the same time, it is relevant to track changes in contract preferences over time and how they affect job satisfaction. For instance, what began as an ‘involuntary temporary’ position might become a personal preference for temporary over permanent contracts. Furthermore, some of the gaps in job satisfaction between different kinds of temporary workers and permanent employees seem to be determined by institutional features. Future studies could investigate these elements and under which mechanisms they operate. For example, involuntary temporary jobs might report lower job satisfaction compared to permanent workers in countries where permanent workers are more protected against dismissals, as these permanent positions guarantee more job security and stability (Balz, 2017). Similarly, it is pertinent to study whether the negative impacts of involuntary temporary employment on job satisfaction might be stronger in certain socio-demographic groups. This could be the case for older workers, who have higher career expectations, or even men in countries where the male breadwinner is more prevalent. Finally, the fact that involuntary temporary workers with short contracts present the largest gaps in job satisfaction raises another question: Are they less satisfied because of the lack of job security or because they experience poorer job quality in general?

## Supplemental Material

sj-pdf-1-eid-10.1177_0143831X221088306 – Supplemental material for Job satisfaction across Europe: An analysis of the heterogeneous temporary workforce in 27 countries Click here for additional data file. Supplemental material, sj-pdf-1-eid-10.1177_0143831X221088306 for Job satisfaction across Europe: An analysis of the heterogeneous temporary workforce in 27 countries by Leandro Iván Canzio, Felix Bühlmann and Jonas Masdonati in Economic and Industrial Democracy

## Notes

1. This article follows the ILO (2016) definition of temporary work: ‘Temporary employment, whereby workers are engaged only for a specific period of time, includes fixed-term, project- or task-based contracts, as well as seasonal or casual work, including day labour.’ Thus, ‘temporary’ and ‘fixed-term’ are used interchangeably.

2. Part of these differences across countries can be attributed to limitations on the extension of temporary contracts, as in the case of Lithuania or Latvia (Tomas, n.d.; Wexels-Riser, n.d.).

3. While including young workers might be problematic, in some countries they constitute a relevant share of the temporary workforce, especially those in apprenticeships. Moreover, the EU-LFS only classifies workers in age groups of 5 years.

4. The questions differ slightly between countries. They normally follow the structure ‘Why do you have a temporary contract instead of a permanent one?’ or ‘Why did you accept a temporary contract?’ The complete questionnaires can be found at: https://ec.europa.eu/eurostat/statistics-explained/index.php/EU_labour_force_survey_-_methodology#Core_questionnaires

5. Eurostat only computes internships and traineeships if workers receive some kind of remuneration for their work.

6. These two groups present several differences and some similarities, but doing separate analyses for each of them was not optimal due to the small sample size in several countries.

7. Further details about these guidelines can be found at: https://ec.europa.eu/eurostat/statistics-explained/index.php?title=EU_labour_force_survey_%E2%80%93_data_and_publication#Publication_guidelines_and_thresholds

8. All the figures were obtained with COEFPLOT (Jann, 2014) for STATA.
