# Supplementary material for: Job satisfaction across Europe: An analysis of the heterogeneous temporary workforce in 27 countries
Source: Econ Ind Democr. 2022 Apr 15;44(3):728–54. doi: 10.1177/0143831X221088306 (PMC10396796; doi:10.1177/0143831X221088306)
Supplement: sj-pdf-1-eid-10.1177_0143831X221088306 – Supplemental material for Job satisfaction across Europe: An analysis of the heterogeneous temporary workforce in 27 countries [file sj-pdf-1-eid-10.1177_0143831X221088306.pdf]

ONLINE APPENDIX

Table of contents

I – Methodological aspects

A. Selection of observations **3**

B. Details about specific variables **3**

C. Robustness tests: Multinomial models **4**

II – Full models

Table A1: Linear regression estimates. Difference in job satisfaction between permanent (ref.) and different kinds of temporary workers for the overall sample - Full models corresponding to Figure 1. **5**

Table A2: Linear regression estimates. Difference in job satisfaction between permanent (ref.) and different kinds of temporary workers, by country - Full models corresponding to Figure 2. **7**

Table A3: Linear regression estimates. Difference in job satisfaction between permanent (ref.) and different kinds of temporary workers with different contract durations for the overall sample - Full models corresponding to Figure 3. **10**

Table A4: Linear regression estimates. Difference in job satisfaction between permanent (ref.) and involuntary temporary workers with different contract durations, by country - Full models corresponding to Figure 4. **12**

Table A5: Linear regression estimates. Difference in job satisfaction between permanent (ref.) and instrumental temporary workers with different contract durations, by country - Full models corresponding to Figure 5. **16**

Table A6: Linear regression estimates. Difference in job satisfaction between permanent (ref.) and voluntary temporary workers with different contract durations, by country - Full models corresponding to Figure 6. **18**

III – Robustness tests

Table B1: Average marginal effects from multinomial logistic regression models. Difference in job satisfaction between permanent (ref.) and different kinds of temporary workers for the overall sample - Replications of models in Figure 1 and Table A1. **20**

Table B2: Average marginal effects from multinomial logistic regression models. Difference in job satisfaction between permanent (ref.) and different kinds of temporary workers, by country - Replications of models in Figure 2 and Table A2. **21**

Table B3: Average marginal effects from multinomial logistic regression models. Difference in job satisfaction between permanent (ref.) and different kinds of temporary workers with different contract durations for the overall sample - Replications of models in Figure 3 and Table A3. **23**

Table B4: Average marginal effects from multinomial logistic regression models. Difference in job satisfaction between permanent (ref.) and involuntary temporary workers with different contract durations, by country - Replications of models in Figure 4 and Table A4 **24**

Table B5: Average marginal effects from multinomial logistic regression models. Difference in job satisfaction between permanent (ref.) and instrumental temporary workers with different contract durations, by country. - Replications of models in Figure 5 and Table A5. **26**

Table B6: Average marginal effects from multinomial logistic regression models. Difference in job satisfaction between permanent (ref.) and voluntary temporary workers with different contract durations, by country. - Replications of models in Figure 6 and Table A6. **27**

Table B7: Linear regression estimates. Determinants of job satisfaction - Income and household composition included as control variables, compared to reference models (Figure 2 and Table A2). **28**

IV- Descriptive statistics of the sample

Table C1: Descriptive statistics of the sample - Correspondence with samples from Figure 1, Table A1 and Table B1. **31**

Table C2: Descriptive statistics of the sample - Correspondence with samples from Figure 2, Table A2 and Table B2. **32**

Table C3: Descriptive statistics of the sample - Correspondence with samples from Figure 3, Table A3 and Table B3. **35**

Table C4: Descriptive statistics of the sample - Correspondence with samples from Figure 4, Table A4 and Table B4. **36**

Table C5: Descriptive statistics of the sample - Correspondence with samples from Figure 5, Table A5 and Table B5. **39**

Table C6: Descriptive statistics of the sample - Correspondence with samples from Figure 6, Table A6 and Table B6. **41**

Table C7: Descriptive statistics of the sample - Correspondence with samples from Table B7. **43**

I - Methodological aspects

A. Selection of observations

Starting with the full original sample from the Ad-Hoc module of the EU-LFS of 2017, the observations that had missing values for the following variables were discarded:

- Job satisfaction.
- Professional status.
- Type of work contract (permanent or temporary).
- Reason for being a temporary worker (among those with temporary contracts).
- Occupation.
- Education.
- Nationality.
- Working time (see the characteristics of working time below).
- Number of hours worked in the second job (among those with a second job).
- Tenure.
- Duration of temporary contract (observations with missing values for this variable were only discarded when contract duration was used as independent variable).

In addition, workers with the following characteristics were discarded:

- Workers whose questionnaire was answered by a third person (proxy interviews).
- Self-employed workers and family workers.
- Workers aged over 64 years.
- Army and military workers.
- Workers who live in a different country than the country where they work.
- Workers who devote more than 10 hours per week to a second job.

Finally, other countries were not analysed because of specific issues:

- Slovenia was not analysed because “reason for being a temporary worker” was missing for the whole sample.
- Iceland and Latvia were not included because they contained too few temporary workers to perform a reliable analysis.
- Croatia was not included because the data for Croatia was not provided by Eurostat.

B. Details about specific variables

Education is introduced as a continuous variable, following the ISCED 2011 scale. This classification avoided collinearity issues with *Occupation*, that was introduced mostly as a categorical variable.

Working time is introduced a category with three items: “Full-time work”, “Part-time work” and “Marginalwork”. In most of the cases, this classification depended on the number of hours worked per week “as usual” in the main job. Hence, “full-time work” refers to more than 30 hours of work per week, “part-time work” refers to between 15 and 30 hours of work per week, and “marginal work” refers to less than 15 hours of work per week “as usual”. In those cases where the number of hours worked per week “as usual” was missing, the number of hours of work during the week of reference was used instead, following the same criteria. If this variable was also missing, the self-classification provided by the worker was used instead. However, workers could only classify themselves as “part-time” or “full-time” workers.Therefore, the analysis might underestimate, to some extent, the number of real “marginal workers”. Insummary, the classification gives priority to the usual number of hours per week, then to the number ofhours of work during the reference week, and finally to the self-classification provided by the worker. Those observations where all these variables were missing were discarded.

C. Robustness tests: Multinomial models

The dependent variable was ordinal (4-point Likert scale), but it was recoded as if it was a continuous one, allowing for the performance of linear regression models. Nonetheless, this violated some regression assumptions. When the dependent variable is ordinal, ordinal logistic regression models aremore appropriate. However, this technique was not fully adequate as it entailed the violation of the assumption of parallel lines. For this reason, multinomial logistic regression models were performed instead. To allow for the comparison of coefficients, average marginal effects are provided instead of odds or odds ratio. As in some countries there were some categories of the dependent variables that contained very few observations, two categories were collapsed: “Not satisfied at all” and “Satisfied to a small extent” were recoded in the same category.

Table B1- B6 report the average marginal effects of the independent variables of these multinomial logistic regression models. These models included the same control variables as the models they replicate (these are indicated in the table).

II - Full models

Table A1: Linear regression estimates. Difference in job satisfaction between permanent (ref.) and different kinds of temporary workers for the overall sample - Full models corresponding to Figure 1.

	B (Robust SE)	B (Robust SE)	B (Robust SE)	B (Robust SE)
**Age (ref: 35 to 44)**				
15 to 24	1.483*** (0.160)			
25 to 34	-0.107 (0.111)			
45 to 54	0.0186 (0.101)			
55 to 64	0.0429 (0.119)			
**Gender (ref: Man)**				
Woman	-0.0419 (0.0815)			
**Educational level**	-0.0325 (0.0277)			
**Working time (ref: Full-time)**				
Part-time	-0.956*** (0.115)			
Marginal work	-0.0811 (0.220)			
**Nationality (ref: Local)**				
EU/EFTA	-1.664*** (0.212)			
Non-EU/EFTA	-1.244*** (0.234)			
**Contract duration**				
Permanent	(ref)	4.056*** (0.159)	-2.168*** (0.240)	-0.433 (0.288)
Involuntary temporary	-4.056*** (0.159)	(ref)	-6.224*** (0.274)	-4.489*** (0.317)
Instrumental temporary	2.168*** (0.240)	6.224*** (0.274)	(ref)	1.734*** (0.360)
Voluntary temporary	0.433 (0.288)	4.489*** (0.317)	-1.734*** (0.360)	(ref)
**Supervisory role (ref: No / DK)**				
Yes	1.685*** (0.0976)			
**Occupation (ref: Elementary occupations)**				
Managers	11.537*** (0.232)			
Professionals	10.885*** (0.183)			
Technicians and associate professionals	8.836*** (0.170)			
Clerical support workers	6.815*** (0.179)			
Service and sales workers	4.781*** (0.161)			
Skilled agricultural, forestry and fisheries	5.336*** (0.400)			
Craft and related trades workers	4.818*** (0.176)			
Plant and machine operators, and assemblers	2.762*** (0.186)			
**Tenure**	0.00297*** (0.000373)			
**Country (ref: Italy)**				
Austria	-1.867*** (0.215)			
Belgium	-4.319*** (0.208)			
Bulgaria	-14.75*** (0.251)			
Switzerland	0.907** (0.308)			
Cyprus	-4.877*** (0.415)			
Czech Republic	-3.720*** (0.207)			

(continued)

Germany	-11.704***			
	(0.218)			
Denmark	0.972***			
	(0.237)			
Estonia	-1.427***			
	(0.306)			
Spain	-3.348***			
	(0.177)			
Finland	-9.615***			
	(0.239)			
France	-9.895***			
	(0.361)			
Greece	-7.928***			
	(0.250)			
Hungary	-6.379***			
	(0.201)			
Ireland	-3.461***			
	(0.245)			
Lithuania	-1.209***			
	(0.299)			
Luxembourg	-13.860***			
	(0.471)			
Malta	3.929***			
	(0.342)			
Netherlands	-9.912***			
	(0.155)			
Norway	-2.593***			
	(0.221)			
Poland	-7.701***			
	(0.204)			
Portugal	-9.767***			
	(0.236)			
Romania	-9.020***			
	(0.193)			
Sweden	2.082***			
	(0.196)			
Slovak Republic	-12.622***			
	(0.315)			
United Kingdom	-10.425***			
	(0.198)			
**Constant**	77.25***	73.19***	79.42***	77.68***
	(0.207)	(0.240)	(0.306)	(0.351)
**Observations**	378112			
**Adjusted R-squared**	0.073			

Note: * p < 0.05, ** p < 0.01, *** p < 0.001.

Table A2: Linear regression estimates. Difference in job satisfaction between permanent (ref.) and different kinds of temporary workers, by country - Full models corresponding to Figure 2.

	Finland	Luxembourg	Cyprus	Norway	Sweden	Malta	Denmark	Estonia	France
	B (Robust SE)	B (Robust SE)	B (Robust SE)	B (Robust SE)	B (Robust SE)	B (Robust SE)	B (Robust SE)	B (Robust SE)	B (Robust SE)
**Age (ref: 35 to 44)**									
15 to 24	2.400**	-0.210	-4.092*	1.575*	-0.0443	2.117	1.538	1.250	0.692
	(0.897)	(2.151)	(1.829)	(0.795)	(0.731)	(1.095)	(0.835)	(1.242)	(1.591)
25 to 34	-0.121	-2.750*	-2.273*	-0.627	-1.626**	1.795	0.588	1.790*	-2.931**
	(0.637)	(1.356)	(1.094)	(0.588)	(0.500)	(0.970)	(0.702)	(0.836)	(1.073)
45 to 54	1.108	-1.829	-0.523	2.185***	0.849	1.005	-0.396	-0.886	0.513
	(0.608)	(1.204)	(1.036)	(0.546)	(0.451)	(1.001)	(0.651)	(0.777)	(0.944)
55 to 64	2.055**	1.869	-2.564*	3.108***	2.024***	2.400*	0.589	-0.939	2.092
	(0.719)	(1.650)	(1.291)	(0.633)	(0.499)	(1.021)	(0.705)	(0.882)	(1.173)
**Gender (ref: Man)**									
Woman	-0.298	-0.714	2.201*	0.782	-0.981**	0.920	0.140	-0.806	-1.004
	(0.469)	(0.995)	(0.867)	(0.418)	(0.339)	(0.692)	(0.439)	(0.630)	(0.775)
**Educational level**	-0.902***	0.101	-0.544	-0.665***	-0.509***	0.0210	-0.496**	-0.109	-0.290
	(0.165)	(0.313)	(0.311)	(0.141)	(0.127)	(0.240)	(0.168)	(0.205)	(0.269)
**Working time (ref: Full-time)**									
Part-time	0.539	2.383	-8.015***	-1.257*	-1.950**	0.714	0.239	-2.074	0.436
	(0.702)	(1.319)	(1.615)	(0.579)	(0.595)	(0.895)	(0.659)	(1.291)	(1.005)
Marginal work	2.339*	6.360** (!)	-15.77***	-2.973**	-0.577	-2.511 (!)	0.948	-8.185**	-0.125
	(1.144)	(2.412)	(4.713)	(0.989)	(1.057)	(2.585)	(0.755)	(2.822)	(2.466)
**Nationality (ref: Local)**									
EU/EFTA	0.0543	-3.029**	0.579	-4.411***	-2.527*	-2.540 (j)	-3.840*	-3.762 (!)	-0.338
	(1.916)	(0.990)	(1.247)	(0.897)	(1.231)	-2,746	(1.582)	(3.308)	(2.699)
Non-EU/EFTA	2.526	-6.665*	14.22***	-3.796**	-4.377**	-	-0.178	-6.548***	0.544
	(2.207)	(2.752)	(1.490)	(1.361)	(1.458)		(1.553)	(1.002)	(2.097)
**Work contract (ref: Permanent)**									
Involuntary temporary	3.209***	1.551	-0.109	-0.247	-0.955	-1.330	-1.403	-1.756 (!)	-2.191
	(0.701)	(2.261)	(1.308)	(1.010)	(0.771)	(2.045)	(1.003)	(4.227)	(1.363)
Instrumental temporary	7.685***	7.370* (!)	0.962	4.161*	1.249	1.138	3.485***	-5.560	5.093*
	(2.240)	(3.095)	(5.621)	(1.953)	(1.342)	(2.192)	(0.994)	(3.159)	(2.093)
Voluntary temporary	2.068	na	na	0.0672	2.274*	3.958	1.118	na	0.343
	(1.256)			(1.387)	(0.923)	(2.493)	(1.058)		(1.941)
**Supervisory role (ref: No / DK)**									
Yes	2.479***	0.919	2.307*	1.177**	0.965**	2.853***	2.166***	1.322	1.166
	(0.572)	(1.014)	(1.087)	(0.432)	(0.355)	(0.697)	(0.610)	(0.698)	(1.025)
**Occupation (ref: Elementary occupations)**									
Managers	12.84***	-0.00433 (k)	9.727***	6.699***	8.447***	-0.00456* (k)	5.452***	17.86***	8.785***
	(1.606)	(0.00272)	(2.382)	(1.356)	(1.130)	(0.00183)	(1.576)	(1.497)	(2.008)
Professionals	7.676***	-	8.234***	4.767***	6.048***	-	2.923**	18.46***	7.396***
	(1.071)		(1.918)	(1.282)	(1.068)		(0.966)	(1.387)	(1.659)
Technicians and associate professionals	7.242***	-	3.750*	4.601***	4.991***	-	3.895***	14.56***	8.119***
	(1.020)		(1.783)	(1.261)	(1.021)		(0.857)	(1.399)	(1.406)
Clerical support workers	5.012***	-	1.040	3.058*	2.354*	-	2.813**	11.22***	4.673**
	(1.194)		(1.665)	(1.380)	(1.150)		(1.002)	(1.587)	(1.565)
Service and sales workers	3.168**	-	-3.330*	2.506*	0.792	-	0.743	8.952***	4.497**
	(0.982)		(1.586)	(1.229)	(1.018)		(0.782)	(1.375)	(1.488)
Skilled agricultural, forestry and fisheries	6.739**	-	2.587	6.962***	2.770	-	4.414	11.37*** (!)	3.372
	(2.212)		(4.611)	(2.093)	(2.052)		(2.469)	(2.641)	(2.797)
Craft and related trades workers	4.054***	-	-2.179	3.807**	3.733***	-	2.954**	9.946***	2.621
	(1.100)		(1.789)	(1.344)	(1.100)		(1.042)	(1.404)	(1.798)
Plant and machine operators, and assemblers	3.579**	-	-2.162	2.249	1.864	-	0.319	5.411***	-0.639
	(1.184)		(2.355)	(1.417)	(1.175)		(1.223)	(1.407)	(1.717)
**Tenure**	-0.00859***	-0.00732	0.0315***	-0.00291	0.000472	0.00148	-0.000283	0.00448	-0.0127***
	(0.00216)	(0.00470)	(0.00419)	(0.00202)	(0.00141)	(0.00285)	(0.00222)	(0.00317)	(0.00346)
**Constant**	72.27***	74.61***	74.51***	80.63***	85.76***	87.66***	84.19***	72.76***	72.38***
	(1.175)	-2,694	(1.824)	(1.338)	(1.103)	-1,722	(1.011)	(1.451)	(1.646)
**Observations**	9553	2944	3763	11314	16657	4379	11020	5615	5134
**Adjusted R-squared**	0.021	0.013	0.104	0.018	0.021	0.009	0.007	0.099	0.016

Note: * p < 0.05, ** p < 0.01, *** p < 0.001. **(i)** Indicates that the categories "Part-time" and "Marginal work" are included under the same category. **(j)** Indicates that the categories "EU/EFTA" and "Non-EU/EFTA" were included under the same category. **(k)** Indicates that Occupation was included as a continuous (instead of categorical) variable. **(!)** Indicates that the coefficient is unreliable because of few observations, according to Eurostat guidelines. **na** Refers to coefficients that are not shown because of the low number of observations.

(continued)

	Austria	Netherlands	Italy	Czechia	Greece	Portugal	Belgium	Spain	Poland
	B<br>(Robust SE)	B<br>(Robust SE)	B<br>(Robust SE)	B<br>(Robust SE)	B<br>(Robust SE)	B<br>(Robust SE)	B<br>(Robust SE)	B<br>(Robust SE)	B<br>(Robust SE)
**Age (ref: 35 to 44)**									
15 to 24	1.463<br>(0.757)	1.419***<br>(0.414)	2.566***<br>(0.553)	0.379<br>(0.789)	-3.347**<br>(1.091)	1.818<br>(0.984)	2.469**<br>(0.796)	2.604***<br>(0.707)	0.950<br>(0.803)
25 to 34	0.219<br>(0.569)	-0.458<br>(0.342)	0.480<br>(0.351)	0.409<br>(0.511)	-2.695***<br>(0.650)	1.901**<br>(0.632)	-0.481<br>(0.503)	0.0260<br>(0.420)	0.190<br>(0.462)
45 to 54	0.866<br>(0.531)	0.522<br>(0.316)	-0.130<br>(0.297)	-0.832<br>(0.464)	0.818<br>(0.557)	-0.896<br>(0.537)	-0.182<br>(0.486)	-0.492<br>(0.362)	-1.201*<br>(0.479)
55 to 64	1.727**<br>(0.663)	0.932**<br>(0.347)	-2.087***<br>(0.372)	-1.275*<br>(0.530)	0.181<br>(0.737)	-1.418*<br>(0.689)	0.0628<br>(0.603)	0.584<br>(0.453)	-0.412<br>(0.541)
**Gender (ref: Man)**									
Woman	1.895***<br>(0.443)	0.459<br>(0.249)	1.071***<br>(0.258)	-1.585***<br>(0.376)	-0.878<br>(0.454)	0.287<br>(0.447)	-0.221<br>(0.402)	0.688*<br>(0.305)	-1.693***<br>(0.370)
**Educational level**	-0.222<br>(0.152)	-0.0257<br>(0.0726)	-0.533***<br>(0.0801)	0.387**<br>(0.143)	0.644***<br>(0.166)	-1.284***<br>(0.160)	-0.256*<br>(0.127)	-0.528***<br>(0.103)	0.654***<br>(0.129)
**Working time (ref: Full-time)**									
Part-time	0.432<br>(0.516)	-1.193***<br>(0.279)	-1.103***<br>(0.291)	1.371<br>(0.824)	-9.034***<br>(0.600)	-2.607**<br>(0.945)	-0.807<br>(0.474)	-2.431***<br>(0.426)	1.252<br>(0.716)
Marginal work	1.981*<br>(0.899)	-1.310**<br>(0.421)	-8.364***<br>(0.953)	4.963*<br>(1.957)	-24.40***<br>(2.008)	-10.69***<br>(1.991)	-0.770<br>(1.311)	-8.810***<br>(0.877)	-2.026<br>(2.082)
**Nationality (ref: Local)**									
EU/EFTA	-3.418***<br>(0.732)	-2.766**<br>(0.871)	0.115<br>(0.550)	-1.711<br>(1.584)	-1.078<br>(1.713)	2.442<br>(2.151)	-0.411<br>(0.649)	0.819<br>(0.946)	2.494 (j)<br>(2.814)
Non-EU/EFTA	-3.348***<br>(0.850)	-4.194***<br>(0.998)	-1.124**<br>(0.427)	1.303<br>(2.088)	-2.080*<br>(0.903)	2.453<br>(1.867)	-4.695***<br>(1.278)	-2.060*<br>(0.867)	-
**Work contract (ref: Permanent)**									
Involuntary temporary	-4.955<br>(3.088)	-1.341**<br>(0.445)	-1.999***<br>(0.387)	-2.071**<br>(0.747)	-2.173**<br>(0.785)	-2.245***<br>(0.646)	-2.316**<br>(0.768)	-2.927***<br>(0.388)	-5.197***<br>(0.549)
Instrumental temporary	3.695***<br>(0.957)	2.672***<br>(0.446)	0.0605<br>(0.642)	na	1.250<br>(1.623)	1.387<br>(1.357)	4.445*<br>(2.158)	1.011<br>(1.180)	-0.910<br>(0.906)
Voluntary temporary	1.177<br>(1.051)	-1.537*<br>(0.601)	2.329<br>(1.653)	1.472<br>(1.328)	1.132<br>(2.865)	1.985<br>(2.052)	0.260<br>(1.371)	0.660<br>(1.572)	-0.827<br>(0.771)
**Supervisory role (ref: No / DK)**									
Yes	2.622***<br>(0.451)	0.587*<br>(0.279)	2.106***<br>(0.293)	2.372***<br>(0.500)	0.281<br>(0.675)	2.344***<br>(0.494)	2.684***<br>(0.467)	0.807*<br>(0.373)	2.073***<br>(0.486)
**Occupation (ref: Elementary occupations)**									
Managers	8.503***<br>(1.143)	4.264***<br>(0.663)	8.482***<br>(1.049)	14.97***<br>(1.197)	18.25***<br>(1.851)	5.701***<br>(1.438)	5.032***<br>(1.056)	9.744***<br>(0.995)	19.83***<br>(1.063)
Professionals	7.567***<br>(0.931)	2.571***<br>(0.491)	9.853***<br>(0.531)	16.30***<br>(0.993)	20.74***<br>(1.016)	4.843***<br>(1.093)	7.058***<br>(0.822)	10.26***<br>(0.649)	20.25***<br>(0.899)
Technicians and associate professionals	7.820***<br>(0.817)	2.773***<br>(0.475)	5.189***<br>(0.470)	12.72***<br>(0.878)	16.72***<br>(1.025)	2.136*<br>(0.896)	5.340***<br>(0.794)	5.242***<br>(0.610)	15.87***<br>(0.849)
Clerical support workers	5.727***<br>(0.904)	1.572**<br>(0.500)	3.805***<br>(0.472)	10.20***<br>(0.934)	12.01***<br>(0.898)	-0.470<br>(0.934)	4.781***<br>(0.777)	4.574***<br>(0.594)	11.23***<br>(0.939)
Service and sales workers	4.026***<br>(0.811)	1.676***<br>(0.438)	3.330***<br>(0.431)	6.845***<br>(0.903)	6.422***<br>(0.826)	-0.560<br>(0.780)	3.168***<br>(0.759)	2.755***<br>(0.508)	7.657***<br>(0.830)
Skilled agricultural, forestry and fisheries	8.972***<br>(2.010)	3.765***<br>(1.108)	3.248**<br>(1.132)	6.636**<br>(2.046)	-1.977<br>(2.575)	1.206<br>(1.627)	2.288<br>(2.617)	3.041*<br>(1.308)	4.745<br>(3.219)
Craft and related trades workers	4.933***<br>(0.874)	1.380*<br>(0.550)	3.468***<br>(0.476)	4.275***<br>(0.901)	6.208***<br>(1.011)	-0.709<br>(0.891)	3.043***<br>(0.830)	2.992***<br>(0.598)	8.550***<br>(0.838)
Plant and machine operators, and assemblers	2.680*<br>(1.062)	1.799**<br>(0.629)	2.005***<br>(0.538)	1.365<br>(0.882)	5.673***<br>(1.064)	-0.426<br>(0.917)	3.327***<br>(0.855)	0.251<br>(0.632)	6.614***<br>(0.876)
**Tenure**	-0.000163<br>(0.00185)	-0.00704***<br>(0.00101)	0.000816<br>(0.00114)	0.00391*<br>(0.00177)	0.0388***<br>(0.00240)	-0.00262<br>(0.00213)	-0.00235<br>(0.00176)	0.00212<br>(0.00141)	0.00707***<br>(0.00172)
**Constant**	75.61***<br>(0.957)	73.70***<br>(0.540)	80.80***<br>(0.498)	70.61***<br>(0.969)	60.33***<br>(0.959)	76.97***<br>(0.872)	76.86***<br>(0.781)	77.72***<br>(0.592)	61.99***<br>(0.903)
**Observations**	14752	30871	35925	14530	11336	13186	15728	29157	18963
**Adjusted R-squared**	0.023	0.009	0.024	0.088	0.202	0.014	0.014	0.031	0.122

Note: * p < 0.05, ** p < 0.01, *** p < 0.001. **(i)** Indicates that the categories "Part-time" and "Marginal work" are included under the same category. **(j)** Indicates that the categories "EU/EFTA" and "Non-EU/EFTA" were included under the same category. **(k)** Indicates that Occupation was included as a continuous (instead of categorical) variable. **(!)** Indicates that the coefficient is unreliable because of few observations, according to Eurostat guidelines. **na** Refers to coefficients that are not shown because of the low number of observations.

(continued)

	Germany	UK	Switzerland	Ireland	Hungary	Bulgaria	Romania	Lithuania	Slovakia
	B (Robust SE)	B (Robust SE)	B (Robust SE)	B (Robust SE)	B (Robust SE)	B (Robust SE)	B (Robust SE)	B (Robust SE)	B (Robust SE)
**Age (ref: 35 to 44)**									
15 to 24	1.801*	-0.851	-0.613	-1.443	-1.412*	0.454	-1.063	0.313	2.623*
	(0.834)	(0.624)	(1.214)	(0.910)	(0.699)	(1.203)	(0.807)	(1.322)	(1.326)
25 to 34	1.178	-1.425**	-0.850	-0.00302	-0.605	1.329*	0.295	-0.674	-0.512
	(0.602)	(0.462)	(0.929)	(0.611)	(0.473)	(0.652)	(0.431)	(0.862)	(0.843)
45 to 54	-0.455	1.310**	0.149	1.421*	-0.860*	0.507	0.0619	-2.070**	-0.642
	(0.566)	(0.457)	(0.822)	(0.607)	(0.426)	(0.561)	(0.382)	(0.741)	(0.773)
55 to 64	-0.248	2.393***	-0.164	2.865***	-0.939	-0.105	-0.490	-2.685***	-0.684
	(0.627)	(0.532)	(0.964)	(0.748)	(0.508)	(0.604)	(0.497)	(0.802)	(0.878)
**Gender (ref: Man)**									
Woman	-0.619	1.834***	-1.429*	1.211*	-1.632***	-2.317***	-1.519***	-2.299***	-3.045***
	(0.451)	(0.359)	(0.671)	(0.502)	(0.354)	(0.444)	(0.321)	(0.605)	(0.616)
**Educational level**	-0.296	0.409***	-0.00803	0.229	0.737***	0.909***	0.643***	1.698***	0.795***
	(0.155)	(0.108)	(0.192)	(0.166)	(0.170)	(0.180)	(0.144)	(0.234)	(0.214)
**Working time (ref: Full-time)**									
Part-time	0.430	-1.742***	1.027	-1.081	-2.944***	-14.11*** (i)	0.228 (i)	-4.737***	-5.665***
	(0.548)	(0.443)	(0.849)	(0.580)	(0.856)	-1,722	-1,595	(1.126)	(1.711)
Marginal work	3.025***	0.685	2.526*	0.463	-2.085	-	-	-10.49***	-19.70***
	(0.770)	(0.828)	(1.158)	(1.256)	(3.207)			(3.019)	(-3.312)
**Nationality (ref: Local)**									
EU/EFTA	0.147	-1.945**	-3.429***	-5.118***	7.603**	-	-	-5.579 (j) (!)	-
	(0.940)	(0.650)	(0.679)	(0.809)	(2.939)			(-4.780)	
Non-EU/EFTA	-1.479	-2.864**	-4.601***	-3.124*	0.986	-	-	-	
	(0.929)	(0.892)	(1.271)	(1.323)	(6.377)				
**Work contract (ref: Permanent)**									
Involuntary temporary	-5.413***	-8.119***	-8.651**	-9.562***	-10.54***	-13.15***	-13.39***	-14.31***	-14.60***
	(1.481)	(1.572)	(3.153)	(1.353)	(0.728)	-1,485	-2,581	(3.718)	(-1.587)
Instrumental temporary	2.064*	2.549	6.877***	5.034*	2.459	-5.113*	na	-6.025 (!)	na
	(0.851)	(2.306)	(1.147)	(2.254)	(1.601)	-2,592		(4.004)	
Voluntary temporary	0.430	-1.577	5.569*	-1.540	-2.827	-7.687*	na	na	12.36***
	(3.887)	(1.481)	(2.811)	(1.904)	(2.062)	(3.545)			(-2.617)
**Supervisory role (ref: No / DK)**									
Yes	1.885***	1.657***	0.797	0.115	2.912***	4.794***	1.826**	2.622**	2.676**
	(0.499)	(0.372)	(0.689)	(0.535)	(0.508)	(0.751)	(0.596)	(0.832)	(0.940)
**Occupation (ref: Elementary occupations)**									
Managers	9.762***	11.12***	5.321**	10.83***	16.43***	20.83***	15.41***	13.83***	25.06***
	(1.294)	(0.822)	(1.951)	(1.267)	(1.066)	(1.444)	(1.356)	(1.485)	(1.964)
Professionals	8.986***	10.06***	5.311**	11.57***	17.71***	20.75***	15.23***	14.20***	23.53***
	(1.001)	(0.759)	(1.783)	(1.094)	(0.935)	(1.133)	(0.867)	(1.268)	(1.584)
Technicians and associate professionals	6.546***	8.316***	5.032**	8.955***	14.44***	16.62***	13.00***	13.52***	19.60***
	(0.874)	(0.771)	(1.724)	(1.084)	(0.733)	(1.064)	(0.834)	(1.340)	(1.441)
Clerical support workers	6.065***	5.564***	4.605*	8.272***	11.15***	14.88***	9.297***	10.14***	16.53***
	(0.903)	(0.788)	(1.847)	(1.104)	(0.808)	(1.069)	(0.886)	(1.580)	(1.486)
Service and sales workers	4.072***	5.128***	0.803	4.630***	9.980***	4.821***	5.862***	4.643***	11.64***
	(0.881)	(0.719)	(1.764)	(1.016)	(0.704)	(0.867)	(0.714)	(1.259)	(1.401)
Skilled agricultural, forestry and fisheries	6.923**	14.76***	0.278	9.383***	7.216***	1.170	5.867**	4.480*	12.96***
	(2.110)	(2.456)	(3.163)	(2.051)	(1.183)	(1.976)	(1.883)	(2.261)	(3.617)
Craft and related trades workers	3.528***	7.439***	3.530	9.382***	6.897***	7.191***	4.468***	5.412***	8.628***
	(0.927)	(0.906)	(1.845)	(1.165)	(0.699)	(0.894)	(0.694)	(1.274)	(1.446)
Plant and machine operators, and assemblers	2.381*	3.995***	1.673	4.569***	3.753***	6.975***	4.083***	5.502***	5.908***
	(1.084)	(0.999)	(2.319)	(1.310)	(0.676)	(0.886)	(0.712)	(1.273)	(1.425)
**Tenure**	-0.00219	-0.0136***	0.00394	0.00196	0.0160***	0.0118***	0.0168***	0.0187***	0.0111***
	(0.00179)	(0.00180)	(0.00329)	(0.00229)	(0.00165)	(0.00246)	(0.00186)	(0.00332)	(0.00281)
**Constant**	68.21***	65.96***	82.97***	71.89***	65.59***	55.02***	63.20***	67.95***	56.26***
	(1.051)	(0.798)	(1.902)	(1.130)	(0.794)	(0.972)	(0.793)	(1.438)	(1.538)
**Observations**	15221	28979	5770	11754	20102	10877	17325	5918	7339
**Adjusted R-squared**	0.016	0.026	0.024	0.043	0.143	0.193	0.095	0.145	0.188

Note: * p < 0.05, ** p < 0.01, *** p < 0.001. **(i)** Indicates that the categories "Part-time" and "Marginal work" are included under the same category. **(j)** Indicates that the categories "EU/EFTA" and "Non-EU/EFTA" were included under the same category. **(k)** Indicates that Occupation was included as a continuous (instead of categorical) variable. **(!)** Indicates that the coefficient is unreliable because of few observations, according to Eurostat guidelines. **na** Refers to coefficients that are not shown because of the low number of observations.

Table A3: Linear regression estimates. Difference in job satisfaction between permanent (ref.) and different kinds of temporary workers with different contract durations for the overall sample - Full models corresponding to Figure 3.

	Involuntary temporary vs. Permanent				Instrumental temporary vs. Permanent	Voluntary temporary vs. Permanent
	B (Robust SE)	B (Robust SE)	B (Robust SE)	B (Robust SE)	B (Robust SE)	B (Robust SE)
**Age (ref: 35 to 44)**						
15 to 24	1.132*** (0.173)				0.871*** (0.178)	0.704*** (0.180)
25 to 34	-0.205 (0.112)				-0.325** (0.115)	-0.311** (0.115)
45 to 54	0.196 (0.101)				0.181 (0.102)	0.174 (0.102)
55 to 64	0.310** (0.113)				0.350** (0.114)	0.392*** (0.114)
**Gender (ref: Man)**						
Woman	-0.106 (0.0839)				-0.284*** (0.0852)	-0.292*** (0.0854)
**Educational level**	-0.0279 (0.0285)				-0.00512 (0.0289)	-0.00543 (0.0290)
**Working time (ref: Full-time)**						
Part-time	-0.876*** (0.120)				-0.501*** (0.123)	-0.459*** (0.123)
Marginal work	0.0653 (0.240)				0.996*** (0.245)	1.179*** (0.238)
**Nationality (ref: Local)**						
EU/EFTA	-1.703*** (0.218)				-1.823*** (0.223)	-1.828*** (0.224)
Non-EU/EFTA	-1.156*** (0.243)				-2.075*** (0.252)	-2.127*** (0.257)
**Contract duration (ref: Permanent)**						
Permanent	(ref)	5.917*** (0.270)	3.749*** (0.253)	2.677*** (0.343)	(ref)	(ref)
6 months or less	-5.917*** (0.270)	(ref)	-2.168*** (0.361)	-3.239*** (0.431)	-0.883 (0.485)	0.0608 (0.504)
Between 7 and 12 months	-3.749*** (0.253)	2.168*** (0.361)	(ref)	-1.072* (0.420)	0.478 (0.612)	-0.351 (0.553)
More than one year	-2.677*** (0.343)	3.239*** (0.431)	1.072* (0.420)	(ref)	4.467*** (0.377)	0.643 (0.544)
**Supervisory role (ref: No / DK)**						
Yes	1.759*** (0.0989)				1.861*** (0.0998)	1.879*** (0.0997)
**Occupation (ref: Elementary occupations)**						
Managers	11.50*** (0.237)				10.79*** (0.242)	10.82*** (0.241)
Professionals	10.93*** (0.189)				10.23*** (0.196)	10.25*** (0.195)
Technicians and associate professionals	8.879*** (0.176)				8.189*** (0.182)	8.227*** (0.182)
Clerical support workers	6.943*** (0.185)				6.319*** (0.192)	6.337*** (0.192)
Service and sales workers	4.845*** (0.168)				4.150*** (0.175)	4.195*** (0.175)
Skilled agricultural, forestry and fisheries	5.601*** (0.416)				4.944*** (0.431)	5.221*** (0.432)
Craft and related trades workers	4.720*** (0.182)				4.163*** (0.188)	4.122*** (0.189)
Plant and machine operators, and assemblers	2.706*** (0.192)				1.900*** (0.200)	1.850*** (0.200)
**Country (ref: Italy)**						
Austria	-2.155*** (0.223)				-1.658*** (0.220)	-1.664*** (0.223)
Belgium	-4.490*** (0.211)				-4.260*** (0.218)	-4.266*** (0.218)
Bulgaria	-14.67*** (0.252)				-13.68*** (0.252)	-13.65*** (0.254)
Switzerland	0.264 (0.328)				1.042*** (0.313)	0.831* (0.329)
Cyprus	-5.075*** (0.417)				-5.905*** (0.443)	-5.866*** (0.443)
Czechia	-4.011*** (0.210)				-3.511*** (0.216)	-3.455*** (0.216)
Germany	-11.98*** (0.226)				-11.56*** (0.223)	-11.58*** (0.230)
Denmark	0.451 (0.246)				0.729** (0.247)	0.699** (0.249)
Estonia	-1.554*** (0.306)				-1.059*** (0.308)	-0.977** (0.309)
Spain	-3.446*** (0.185)				-3.322*** (0.192)	-3.338*** (0.194)

(continued)

Finland	-9.992***				-10.33***	-10.27***
	(0.244)				(0.255)	(0.254)
France	-10.19***				-10.05***	-10.11***
	(0.374)				(0.383)	(0.383)
Greece	-7.894***				-6.930***	-6.812***
	(0.253)				(0.263)	(0.266)
Hungary	-6.523***				-4.802***	-4.835***
	(0.203)				(0.206)	(0.207)
Ireland	-3.554***				-2.995***	-3.041***
	(0.251)				(0.252)	(0.253)
Lithuania	-1.447***				-0.868**	-0.805**
	(0.299)				(0.300)	(0.301)
Luxembourg	-14.23***				-13.88***	-14.00***
	(0.478)				(0.484)	(0.489)
Malta	3.703***				4.065***	4.101***
	(0.349)				(0.350)	(0.352)
Netherlands	-10.34***				-10.23***	-10.23***
	(0.162)				(0.166)	(0.167)
Norway	-3.024***				-2.519***	-2.572***
	(0.226)				(0.228)	(0.229)
Poland	-7.609***				-6.558***	-6.503***
	(0.213)				(0.221)	(0.221)
Portugal	-9.814***				-9.960***	-10.02***
	(0.243)				(0.256)	(0.258)
Romania	-9.252***				-8.660***	-8.638***
	(0.195)				(0.197)	(0.198)
Sweden	1.823***				2.199***	2.194***
	(0.201)				(0.205)	(0.205)
Slovak Republic	-12.86***				-10.82***	-10.75***
	(0.318)				(0.314)	(0.313)
United Kingdom	-10.67***				-10.20***	-10.23***
	(0.200)				(0.202)	(0.203)
**Constant**	77.74***	71.83***	73.99***	75.07***	77.85***	77.83***
	(0.208)	(0.324)	(0.316)	(0.398)	(0.215)	(0.216)
**Observations**	357222				339346	337257
**Adjusted R-squared**	0.073				0.068	0.068

Note: * $p < 0.05$, ** $p < 0.01$, *** $p < 0.001$.

Table A4: Linear regression estimates. Difference in job satisfaction between permanent (ref.) and involuntary temporary workers with different contract durations, by country - Full models corresponding to Figure 4.

	Luxembourg	Finland	Norway	Sweden	Denmark	France	Netherlands	Malta
	B (Robust SE)	B (Robust SE)	B (Robust SE)	B (Robust SE)	B (Robust SE)	B (Robust SE)	B (Robust SE)	B (Robust SE)
**Age (ref: 35 to 44)**								
15 to 24	2.256	3.007**	1.102	-0.265	0.992	1.818	2.026***	2.428*
	(2.172)	(0.944)	(0.831)	(0.820)	(0.898)	(1.831)	(0.455)	(1.083)
25 to 34	-2.531	0.201	-0.521	-1.793***	0.695	-2.436*	-0.0644	1.946*
	(1.333)	(0.641)	(0.593)	(0.507)	(0.718)	(1.091)	(0.361)	(0.954)
45 to 54	-2.379*	0.469	1.977***	0.787	-0.307	-0.486	0.174	1.163
	(1.176)	(0.597)	(0.540)	(0.449)	(0.648)	(0.918)	(0.323)	(0.998)
55 to 64	0.766	0.782	2.600***	2.000***	0.602	0.240	0.0385	2.539*
	(1.578)	(0.648)	(0.590)	(0.468)	(0.670)	(1.094)	(0.334)	(1.021)
**Gender (ref: Man)**								
Woman	-0.591	-0.351	0.576	-0.964**	0.160	-1.073	0.389	0.849
	(1.011)	(0.480)	(0.427)	(0.348)	(0.459)	(0.806)	(0.269)	(0.703)
**Educational level**	0.0852	-0.845***	-0.644***	-0.543***	-0.516**	-0.108	0.0489	0.000533
	(0.314)	(0.168)	(0.144)	(0.131)	(0.175)	(0.281)	(0.0766)	(0.246)
**Working time (ref: Full-time)**								
Part-time	2.482	0.580	-1.130	-1.848**	0.449	0.588	-1.081***	0.925
	(1.331)	(0.727)	(0.600)	(0.639)	(0.694)	(1.041)	(0.301)	(0.926)
Marginal work	8.191** (!)	2.510	-2.734*	-1.634	1.647	-0.251	-1.133*	-3.220 (!)
	(2.575)	(1.305)	(1.088)	(1.417)	(0.851)	(2.843)	(0.467)	(2.988)
**Nationality (ref: Local)**								
EU/EFTA	-2.700**	0.455	-4.262***	-2.146	-3.406*	0.843	-2.174*	-3.255 (j)
	(0.983)	(1.978)	(0.915)	(1.260)	(1.660)	(2.722)	(0.914)	(2.882)
Non-EU/EFTA	-6.718*	3.431	-3.897**	-3.562*	-1.064	1.419	-3.814***	-
	(2.774)	(2.289)	(1.448)	(1.559)	(1.672)	(2.204)	(1.152)	
**Contract duration (ref: Permanent)**								
6 months or less	na	4.153***	-0.217	-1.360	-1.601	-1.882	-2.063	-3.462 (!)
		(0.977)	(3.018)	(1.362)	(2.288)	(1.760)	(1.214)	(3.713)
Between 7 and 12 months	6.051 (!)	4.552***	3.546	0.325	1.538	0.726	-0.462	0.751 (!)
	(4.404)	(1.075)	(2.147)	(1.445)	(1.594)	(2.206)	(0.599)	(3.290)
More than one year	4.901	2.106	-3.966*	0.0586	-2.729*	-1.581	1.289	-2.272 (!)
	(2.594)	(1.438)	(1.761)	(1.170)	(1.373)	(3.321)	(1.331)	(3.469)
**Supervisory role (ref: No / DK)**								
Yes	1.081	2.381***	1.103*	1.100**	2.204***	1.349	0.541	3.097***
	(1.022)	(0.578)	(0.434)	(0.360)	(0.617)	(1.035)	(0.288)	(0.709)
**Occupation (ref: Elementary occupations)**								
Managers	-0.00411 (k)	12.77***	6.673***	8.216***	5.451***	7.900***	4.311***	-0.00439* (k)
	(0.00274)	(1.637)	(1.394)	(1.211)	(1.599)	(2.065)	(0.702)	(0.00189)
Professionals	-	7.366***	4.788***	5.877***	2.858**	6.494***	2.371***	-
		(1.110)	(1.325)	(1.158)	(1.014)	(1.730)	(0.540)	
Technicians and associate professionals	-	6.920***	4.609***	4.886***	3.833***	7.695***	2.538***	-
		(1.057)	(1.300)	(1.109)	(0.892)	(1.458)	(0.521)	
Clerical support workers	-	4.653***	3.112*	2.356	2.699*	4.687**	1.754**	-
		(1.231)	(1.420)	(1.240)	(1.048)	(1.623)	(0.548)	
Service and sales workers	-	3.064**	2.597*	0.532	0.899	4.332**	1.847***	-
		(1.024)	(1.273)	(1.118)	(0.831)	(1.559)	(0.491)	
Skilled agricultural, forestry and fisheries	-	6.274**	7.228**	1.754	5.301	2.248	3.143**	-
		(2.327)	(2.201)	(2.262)	(2.719)	(3.293)	(1.185)	
Craft and related trades workers	-	3.889***	3.647**	3.414**	3.066**	2.445	1.324*	-
		(1.136)	(1.388)	(1.186)	(1.121)	(1.904)	(0.602)	
Plant and machine operators, and assemblers	-	3.130*	1.800	1.863	0.205	0.0487	1.754*	-
		(1.225)	(1.465)	(1.252)	(1.260)	(1.788)	(0.690)	
**Constant**	73.48***	71.48***	80.49***	86.13***	84.17***	70.42***	72.55***	87.64***
	-2555	(1.190)	(1.363)	(1.166)	(1.030)	(1.664)	(0.568)	(1.718)
**Observations**	2872	9129	10775	15551	10194	4768	27580	4246
**Adjusted R-squared**	0.014	0.017	0.018	0.021	0.006	0.012	0.005	0.010

Note: * $p < 0.05$, ** $p < 0.01$, *** $p < 0.001$. **(i)** Indicates that the categories "Part-time" and "Marginal work" are included under the same category. **(j)** Indicates that the categories "EU/EFTA" and "Non-EU/EFTA" were included under the same category. **(k)** Indicates that Occupation was included as a continuous (instead of categorical) variable. **(!)** Indicates that the coefficient is unreliable because of few observations, according to Eurostat guidelines. **na** Refers to coefficients that are not shown because of the low number of observations.

(continued)

	Cyprus	Ireland	Austria	Italy	Portugal	Czechia	Spain	Belgium
	B (Robust SE)	B (Robust SE)	B (Robust SE)	B (Robust SE)	B (Robust SE)	B (Robust SE)	B (Robust SE)	B (Robust SE)
**Age (ref: 35 to 44)**								
15 to 24	-6.756*** (1.843)	-1.960* (0.939)	0.942 (0.796)	2.018** (0.618)	2.456* (1.091)	0.601 (0.808)	3.545*** (0.841)	3.036*** (0.819)
25 to 34	-4.039*** (1.081)	-0.187 (0.610)	0.443 (0.580)	0.418 (0.356)	2.227*** (0.638)	0.175 (0.505)	0.0900 (0.446)	-0.354 (0.497)
45 to 54	0.885 (1.024)	1.357* (0.605)	0.971 (0.526)	-0.0963 (0.291)	-0.907 (0.529)	-0.629 (0.460)	-0.313 (0.365)	-0.312 (0.473)
55 to 64	0.120 (1.239)	3.029*** (0.704)	1.757** (0.619)	-2.041*** (0.341)	-1.913** (0.647)	-1.020* (0.510)	0.777 (0.418)	-0.300 (0.559)
**Gender (ref: Man)**								
Woman	2.242* (0.876)	1.156* (0.512)	2.210*** (0.465)	0.961*** (0.265)	0.0661 (0.457)	-1.645*** (0.380)	0.455 (0.322)	-0.217 (0.409)
**Educational level**	-0.624* (0.313)	0.280 (0.169)	-0.163 (0.159)	-0.554*** (0.0811)	-1.341*** (0.164)	0.358* (0.145)	-0.489*** (0.107)	-0.203 (0.128)
**Working time (ref: Full-time)**								
Part-time	-9.077*** (1.622)	-0.874 (0.592)	0.297 (0.533)	-1.049*** (0.300)	-1.817 (0.994)	1.422 (0.867)	-2.222*** (0.459)	-0.867 (0.482)
Marginal work	-16.05** (4.967)	0.407 (1.346)	2.098* (0.940)	-8.346*** (1.002)	-11.78*** (2.302)	5.321* (2.615)	-8.108*** (1.005)	-1.622 (1.482)
**Nationality (ref: Local)**								
EU/EFTA	-0.936 -1,239	-5.470*** (0.819)	-3.150*** (0.764)	0.303 (0.551)	2.447 -2,306	-1.732 (1.624)	0.910 (1.028)	-0.406 (0.654)
Non-EU/EFTA	12.19*** (1.601)	-3.514* (1.389)	-3.250*** (0.886)	-1.103* (0.436)	3.725* (1.857)	1.289 (2.093)	-0.998 (0.933)	-4.582*** (1.325)
**Contract duration (ref: Permanent)**								
6 months or less	-4.677 (2.517)	-5.711 (4.038)	-6.225 (!) (6.217)	-2.244*** (0.519)	-3.674*** (0.947)	-4.484* (1.867)	-4.549*** (0.604)	-4.967*** (1.064)
Between 7 and 12 months	-3.420* (1.585)	-10.80** (3.563)	-6.889 (!) (4.427)	-0.324 (0.554)	-0.734 (0.826)	-2.616* (1.165)	-0.507 (0.804)	1.201 (1.192)
More than one year	2.976 (1.846)	-11.32*** (3.071)	na	-1.103 (1.419)	3.633* (1.656)	-1.617 (1.020)	0.568 (1.265)	-0.169 (1.778)
**Supervisory role (ref: No / DK)**								
Yes	3.182** (1.086)	0.171 (0.537)	2.806*** (0.456)	2.169*** (0.296)	2.248*** (0.501)	2.445*** (0.502)	0.886* (0.381)	2.744*** (0.470)
**Occupation (ref: Elementary occupations)**								
Managers	10.61*** -2,397	10.58*** (1.293)	8.039*** (1.169)	8.275*** (1.064)	5.800*** -1,462	15.35*** (1.218)	9.142*** (1.025)	4.445*** (1.061)
Professionals	9.643*** -1,914	11.19*** (1.126)	7.315*** (0.960)	9.484*** (0.540)	4.793*** -1,119	16.79*** (1.009)	9.563*** (0.692)	6.293*** (0.825)
Technicians and associate professionals	5.542** -1,773	8.766*** (1.114)	7.546*** (0.837)	4.928*** (0.479)	1.938* (0.908)	13.07*** (0.894)	4.657*** (0.650)	4.817*** (0.798)
Clerical support workers	2.570 (1.672)	8.160*** (1.134)	5.670*** (0.923)	3.681*** (0.480)	-0.688 (0.957)	10.55*** (0.950)	4.263*** (0.631)	4.392*** (0.781)
Service and sales workers	-2.725 (1.602)	4.507*** (1.055)	4.074*** (0.835)	3.070*** (0.443)	-0.529 (0.797)	7.109*** (0.927)	2.688*** (0.550)	2.804*** (0.771)
Skilled agricultural, forestry and fisheries	3.109 (4.530)	9.776*** (2.096)	9.303*** (2.191)	3.102** (1.174)	1.073 (1.674)	7.077*** (2.084)	2.943* (1.410)	2.742 (2.535)
Craft and related trades workers	-1.099 (1.802)	9.170*** (1.194)	4.740*** (0.902)	3.315*** (0.489)	-0.832 (0.909)	4.634*** (0.918)	2.477*** (0.663)	2.701** (0.836)
Plant and machine operators, and assemblers	-1.688 (2.392)	4.324** (1.349)	2.594* (1.087)	1.669** (0.553)	-0.593 (0.939)	1.657 (0.900)	-0.0858 (0.679)	3.138*** (0.864)
**Constant**	77.69*** -1,780	72.18*** (1.129)	75.27*** (0.965)	81.24*** (0.481)	76.92*** (0.857)	70.84*** (0.973)	78.19*** (0.618)	76.78*** (0.777)
**Observations**	3724	11263	13558	34183	12379	14231	25517	15371
**Adjusted R-squared**	0.094	0.038	0.022	0.023	0.015	0.088	0.026	0.015

Note: * p < 0.05, ** p < 0.01, *** p < 0.001. **(i)** Indicates that the categories "Part-time" and "Marginal work" are included under the same category. **(j)** Indicates that the categories "EU/EFTA" and "Non-EU/EFTA" were included under the same category. **(k)** Indicates that Occupation was included as a continuous (instead of categorical) variable. **(!)** Indicates that the coefficient is unreliable because of few observations, according to Eurostat guidelines. **na** Refers to coefficients that are not shown because of the low number of observations.

(continued)

	Germany	Greece	Poland	UK	Switzerland	Slovakia	Hungary
	B<br>(Robust SE)	B<br>(Robust SE)	B<br>(Robust SE)	B<br>(Robust SE)	B<br>(Robust SE)	B<br>(Robust SE)	B<br>(Robust SE)
**Age (ref: 35 to 44)**							
15 to 24	1.710	-6.408***	1.096	-0.0262	-1.075	1.980	-2.183**
	(0.919)	(1.133)	(0.951)	(0.628)	(1.316)	(1.329)	(0.703)
25 to 34	1.347*	-4.512***	-0.0833	-0.888	-0.913	-0.976	-1.412**
	(0.611)	(0.653)	(0.477)	(0.461)	(0.948)	(0.831)	(0.469)
45 to 54	-0.565	3.427***	-0.437	0.801	0.293	-0.0122	-0.241
	(0.554)	(0.538)	(0.472)	(0.455)	(0.821)	(0.754)	(0.421)
55 to 64	-0.488	4.797***	0.219	1.295*	0.199	0.277	0.174
	(0.597)	(0.686)	(0.510)	(0.518)	(0.919)	(0.825)	(0.491)
**Gender (ref: Man)**							
Woman	-0.607	-1.101*	-2.003***	1.846***	-1.470*	-3.091***	-1.850***
	(0.474)	(0.464)	(0.387)	(0.364)	(0.728)	(0.619)	(0.358)
**Educational level**	-0.297	0.514**	0.717***	0.462***	-0.0197	0.694**	0.677***
	(0.161)	(0.170)	(0.134)	(0.109)	(0.200)	(0.216)	(0.172)
**Working time (ref: Full-time)**							
Part-time	0.320	-9.207***	1.603*	-1.744***	0.938	-7.218***	-2.842**
	(0.562)	(0.619)	(0.814)	(0.450)	(0.894)	(1.826)	(0.884)
Marginal work	2.736***	-26.06***	-4.061	1.120	2.617*	-24.15***	-3.892
	(0.794)	(2.108)	(3.041)	(0.863)	(1.194)	(3.791)	(3.340)
**Nationality (ref: Local)**						-	
EU/EFTA	0.317	-1.914	3.326 (j)	-1.500*	-3.836***	-	6.013*
	(0.964)	(1.729)	(3.295)	(0.658)	(0.717)		(2.943)
Non-EU/EFTA	-1.645	-2.779**	-	-2.346**	-5.249***	-	0.664
	(0.992)	(0.918)		(0.904)	(1.400)		(6.527)
**Contract duration (ref: Permanent)**							
6 months or less	-6.955*	-7.533***	-8.236***	-9.962*	-10.26*	-14.93***	-15.70***
	(3.320)	(1.073)	(1.199)	(3.977)	(4.016)	(2.073)	(1.214)
Between 7 and 12 months	-6.909**	-5.926***	-5.969***	-3.065	-7.431	-14.72***	-9.403***
	(2.106)	(1.106)	(0.854)	(3.489)	(5.338)	(2.220)	(0.877)
More than one year	-0.441	-0.407	-5.065***	-5.035	na	-15.38***	-11.54***
	(2.470)	(1.821)	(0.690)	(3.141)		(4.155)	(2.177)
**Supervisory role (ref: No / DK)**							
Yes	1.807***	1.020	1.961***	1.372***	0.826	2.929**	3.187***
	(0.506)	(0.687)	(0.505)	(0.373)	(0.697)	(0.942)	(0.511)
**Occupation (ref: Elementary occupations)**							
Managers	9.052***	20.12***	19.11***	10.89***	5.734**	25.56***	17.40***
	(1.315)	(1.896)	(1.116)	(0.834)	(2.005)	(1.983)	(1.075)
Professionals	8.454***	22.93***	19.48***	9.739***	5.894**	24.28***	19.03***
	(1.030)	(1.031)	(0.953)	(0.774)	(1.844)	(1.599)	(0.941)
Technicians and associate professionals	5.708***	18.70***	15.32***	8.050***	5.582**	20.01***	15.41***
	(0.899)	(1.034)	(0.902)	(0.784)	(1.781)	(1.458)	(0.736)
Clerical support workers	5.537***	13.59***	10.75***	5.354***	5.400**	16.73***	11.84***
	(0.924)	(0.912)	(0.999)	(0.803)	(1.915)	(1.510)	(0.815)
Service and sales workers	3.741***	6.910***	6.975***	5.115***	1.185	11.81***	10.48***
	(0.904)	(0.845)	(0.896)	(0.735)	(1.833)	(1.425)	(0.714)
Skilled agricultural, forestry and fisheries	7.048**	-0.673	3.286	15.34***	-0.149	14.98***	7.340***
	(2.187)	(2.781)	(3.424)	(2.427)	(3.851)	(3.580)	(1.198)
Craft and related trades workers	2.645**	7.212***	7.627***	7.147***	3.468	8.807***	7.547***
	(0.956)	(1.028)	(0.893)	(0.921)	(1.953)	(1.466)	(0.707)
Plant and machine operators, and assemblers	1.597	5.872***	5.957***	4.031***	2.257	5.877***	4.177***
	(1.112)	(1.083)	(0.930)	(1.018)	(2.405)	(1.448)	(0.685)
**Constant**	68.61***	64.43***	63.38***	64.69***	83.11***	57.68***	66.92***
	(1.055)	(0.956)	(0.942)	(0.800)	(1.943)	(1.548)	(0.797)
**Observations**	14109	11059	16983	28276	5221	7199	19702
**Adjusted R-squared**	0.015	0.184	0.123	0.023	0.020	0.190	0.142

Note: * $p < 0.05$, ** $p < 0.01$, *** $p < 0.001$. **(i)** Indicates that the categories "Part-time" and "Marginal work" are included under the same category. **(j)** Indicates that the categories "EU/EFTA" and "Non-EU/EFTA" were included under the same category. **(k)** Indicates that Occupation was included as a continuous (instead of categorical) variable. **(!)** Indicates that the coefficient is unreliable because of few observations, according to Eurostat guidelines. **na** Refers to coefficients that are not shown because of the low number of observations.

(continued)

	Bulgaria	Romania
	B<br>(Robust SE)	B<br>(Robust SE)
Age (ref: 35 to 44)		
15 to 24	-0.232<br>(1.235)	-2.227**<br>(0.799)
25 to 34	0.784<br>(0.649)	-0.388<br>(0.425)
45 to 54	0.762<br>(0.554)	0.574<br>(0.379)
55 to 64	0.526<br>(0.590)	0.639<br>(0.482)
Gender (ref: Man)		
Woman	-2.296***<br>(0.447)	-1.563***<br>(0.322)
Educational level	0.933***<br>(0.181)	0.599***<br>(0.144)
Working time (ref: Full-time)		
Part-time	-15.10***<br>(1.844)	0.329<br>(1.613)
Marginal work	-	-
Nationality (ref: Local)	-	-
EU/EFTA	-	-
Non-EU/EFTA	-	-
Work contract (ref: Permanent)		
6 months or less	-16.28***<br>(1.818)	-17.77*** (!)<br>(3.733)
Between 7 and 12 months	-6.732*<br>(3.226)	-14.88***<br>(4.254)
More than one year	na	na
Supervisory role (ref: No / DK)		
Yes	4.960***<br>(0.752)	1.968**<br>(0.599)
Occupation (ref: Elementary occupations)		
Managers	20.64***<br>(1.451)	16.02***<br>(1.353)
Professionals	21.14***<br>(1.138)	16.15***<br>(0.861)
Technicians and associate professionals	16.94***<br>(1.074)	13.75***<br>(0.831)
Clerical support workers	14.85***<br>(1.081)	9.834***<br>(0.884)
Service and sales workers	4.711***<br>(0.885)	6.032***<br>(0.713)
Skilled agricultural, forestry and fisheries	2.797<br>(2.106)	6.418***<br>(1.887)
Craft and related trades workers	7.257***<br>(0.906)	4.974***<br>(0.691)
Plant and machine operators, and assemblers	7.022***<br>(0.897)	4.497***<br>(0.710)
**Constant**	56.05***<br>(0.976)	64.58***<br>(0.778)
**Observations**	10644	17301
**Adjusted R-squared**	0.185	0.090

Note: * $p < 0.05$, ** $p < 0.01$, *** $p < 0.001$. **(i)** Indicates that the categories "Part-time" and "Marginal work" are included under the same category. **(j)** Indicates that the categories "EU/EFTA" and "Non EU/EFTA" were included under the same category. **(k)** Indicates that Occupation was included as a continuous (instead of categorical) variable. **(!)** Indicates that the coefficient is unreliable because of few observations, according to Eurostat guidelines. **na** Refers to coefficients that are not shown because of the low number of observations.

Table A5: Linear regression estimates. Difference in job satisfaction between permanent (ref.) and instrumental temporary workers with different contract durations, by country - Full models corresponding to Figure 5.

	Ireland	Finland	Sweden	Switzerland	Portugal	UK	Greece	Spain	Italy
	B (Robust SE)	B (Robust SE)	B (Robust SE)	B (Robust SE)	B (Robust SE)	B (Robust SE)	B (Robust SE)	B (Robust SE)	B (Robust SE)
**Age (ref: 35 to 44)**									
15 to 24	-1.997*	3.037**	-0.680	-1.007	1.758	0.0189	-5.761***	4.607***	1.999**
	(0.950)	(1.048)	(0.864)	(1.244)	(1.398)	(0.630)	(1.200)	(0.984)	(0.665)
25 to 34	-0.197	0.271	-1.716***	-0.893	1.736*	-0.822	-5.047***	0.322	0.330
	(0.611)	(0.687)	(0.517)	(0.921)	(0.701)	(0.462)	(0.684)	(0.482)	(0.376)
45 to 54	1.506*	0.346	0.858	0.391	-0.898	0.858	3.624***	-0.551	-0.152
	(0.606)	(0.626)	(0.451)	(0.818)	(0.553)	(0.456)	(0.561)	(0.379)	(0.304)
55 to 64	3.170***	0.728	2.044***	0.348	-2.099**	1.302*	4.963***	0.882*	-1.942***
	(0.707)	(0.679)	(0.473)	(0.913)	(0.672)	(0.519)	(0.711)	(0.431)	(0.351)
**Gender (ref: Man)**									
Woman	1.182*	-0.306	-1.047**	-1.338*	0.293	1.842***	-1.491**	0.394	0.837**
	(0.513)	(0.506)	(0.357)	(0.677)	(0.488)	(0.364)	(0.482)	(0.338)	(0.277)
**Educational level**	0.273	-0.778***	-0.471***	-0.0135	-1.183***	0.488***	0.773***	-0.357**	-0.625***
	(0.169)	(0.179)	(0.134)	(0.192)	(0.175)	(0.109)	(0.175)	(0.113)	(0.0840)
**Working time (ref: Full-time)**									
Part-time	-1.055	0.612	-2.124**	1.153	-1.235	-1.685***	-8.253***	-1.638**	-0.943**
	(0.596)	(0.792)	(0.675)	(0.855)	(1.229)	(0.451)	(0.652)	(0.498)	(0.316)
Marginal work	-0.0958	3.600*	-1.096	2.860*	-8.062**	1.212	-24.64***	-6.750***	-7.514***
	(1.368)	(1.489)	(1.626)	(1.172)	(2.567)	(0.866)	(2.414)	(1.173)	(1.137)
**Nationality (ref: Local)**									
EU/EFTA	-5.350***	0.458	-2.374	-3.489***	1.305	-1.443*	-2.503	1.383	0.542
	(0.816)	(2.055)	(1.317)	(0.679)	(2.724)	(0.660)	(2.038)	(1.113)	(0.591)
Non-EU/EFTA	-3.008*	2.800	-5.108**	-4.550***	3.099	-2.274*	-4.239***	-0.766	-1.029*
	(1.360)	(2.494)	(1.789)	(1.282)	(2.397)	(0.904)	(1.004)	(1.039)	(0.456)
**Contract duration (ref: Permanent)**									
6 months or less	na	6.924*	3.008*	3.322	1.304	0.506	0.206	-0.0901	-0.540
		(3.067)	(1.431)	(2.711)	(2.203)	(4.724)	(2.436)	(1.866)	(0.931)
Between 7 and 12 months	5.558	na	-3.597	3.997	1.868	na	0.317	-1.655	-0.858
	(4.243)		(3.967)	(2.641)	(2.009)		(3.108)	(2.189)	(1.599)
More than one year	4.916	5.597	na	7.834***	na	5.638	-4.566	3.125	1.796
	(4.234)	(4.360)		(1.261)		(3.479)	(2.803)	(2.136)	(0.938)
**Supervisory role (ref: No / DK)**									
Yes	0.241	2.399***	1.127**	0.879	2.547***	1.361***	0.976	0.922*	2.271***
	(0.536)	(0.593)	(0.362)	(0.693)	(0.522)	(0.373)	(0.689)	(0.387)	(0.302)
**Occupation (ref: Elementary occupations)**									
Managers	10.64***	13.43***	8.330***	5.867**	5.324***	10.89***	18.70***	8.912***	8.499***
	(1.287)	(1.720)	(1.262)	(1.976)	(1.541)	(0.835)	(1.925)	(1.059)	(1.047)
Professionals	10.91***	7.992***	6.105***	6.006***	4.385***	9.661***	21.05***	9.274***	9.250***
	(1.130)	(1.218)	(1.217)	(1.800)	(1.228)	(0.776)	(1.097)	(0.736)	(0.569)
Technicians and associate professionals	8.643***	7.301***	4.959***	5.562**	2.288*	7.991***	17.58***	4.532***	4.779***
	(1.119)	(1.165)	(1.167)	(1.743)	(0.986)	(0.786)	(1.094)	(0.685)	(0.506)
Clerical support workers	7.989***	5.384***	2.526	4.925**	-0.178	5.411***	12.47***	4.108***	3.483***
	(1.140)	(1.356)	(1.305)	(1.866)	(1.054)	(0.805)	(0.987)	(0.669)	(0.509)
Service and sales workers	4.517***	3.441**	1.079	1.134	0.131	5.162***	5.949***	2.714***	2.739***
	(1.059)	(1.132)	(1.184)	(1.786)	(0.899)	(0.737)	(0.933)	(0.595)	(0.475)
Skilled agricultural, forestry and fisheries	10.43***	6.464*	0.0720	0.776	1.133	14.74***	-1.454	2.374	4.040**
	(2.117)	(2.602)	(2.590)	(3.269)	(1.927)	(2.487)	(2.815)	(1.532)	(1.452)
Craft and related trades workers	8.994***	4.799***	3.875**	4.022*	-0.358	7.286***	6.516***	2.542***	3.015***
	(1.199)	(1.231)	(1.237)	(1.859)	(1.005)	(0.922)	(1.106)	(0.701)	(0.516)
Plant and machine operators, and assemblers	3.975**	4.003**	2.086	1.866	0.0499	4.309***	4.511***	0.0652	1.559**
	(1.352)	(1.316)	(1.309)	(2.361)	(1.043)	(1.018)	(1.161)	(0.732)	(0.584)
**Constant**	72.28***	70.61***	85.62***	82.68***	76.02***	64.52***	64.58***	77.64***	81.66***
	(1.129)	(1.290)	(1.228)	(1.894)	(0.942)	(0.802)	(1.019)	(0.657)	(0.514)
**Observations**	11143	8222	14959	5653	10691	28183	10089	22431	31582
**Adjusted R-squared**	0.034	0.018	0.020	0.021	0.010	0.022	0.165	0.021	0.019

Note: * $p < 0.05$, ** $p < 0.01$, *** $p < 0.001$. **(i)** Indicates that the categories "Part-time" and "Marginal work" are included under the same category. **(j)** Indicates that the categories "EU/EFTA" and "Non-EU/EFTA" were included under the same category. **(!)** Indicates that the coefficient is unreliable because of few observations, according to Eurostat guidelines. **na** Refers to coefficients that are not shown because of the low number of observations.

(continued)

	Germany	Denmark	France	Poland	Austria
	B (Robust SE)	B (Robust SE)	B (Robust SE)	B (Robust SE)	B (Robust SE)
**Age (ref: 35 to 44)**					
15 to 24	1.751* (0.845)	1.186 (0.882)	2.976 (2.061)	1.516 (1.104)	1.162 (0.772)
25 to 34	1.335* (0.603)	0.442 (0.722)	-1.887 (1.129)	-0.0291 (0.503)	0.307 (0.575)
45 to 54	-0.519 (0.553)	-0.259 (0.653)	-0.606 (0.944)	-0.371 (0.491)	0.945 (0.526)
55 to 64	-0.468 (0.596)	0.788 (0.670)	-0.505 (1.135)	1.071* (0.523)	1.741** (0.618)
**Gender (ref: Man)**					
Woman	-0.728 (0.455)	0.173 (0.461)	-1.026 (0.825)	-1.976*** (0.403)	2.086*** (0.450)
**Educational level**	-0.265 (0.156)	-0.535** (0.175)	-0.0550 (0.287)	0.776*** (0.138)	-0.218 (0.156)
**Working time (ref: Full-time)**					
Part-time	0.510 (0.553)	0.481 (0.697)	0.708 (1.110)	2.559** (i) (0.840)	0.186 (0.526)
Marginal work	2.848*** (0.779)	1.151 (0.833)	1.290 (3.043)	-	2.290* (0.931)
**Nationality (ref: Local)**					
EU/EFTA	0.267 (0.955)	-4.203* (1.679)	-0.101 (2.825)	0.927 (j) (3.837)	-3.203*** (0.754)
Non-EU/EFTA	-1.293 (0.930)	-0.164 (1.670)	0.444 (2.334)	-	-3.072*** (0.852)
**Contract duration (ref: Permanent)**					
6 months or less	-0.649 (2.220)	-1.796 (2.872)	-1.882 (4.349)	-2.567 (1.338)	-6.357* (2.731)
Between 7 and 12 months	-1.169 (1.879)	5.506** (2.028)	na	-1.528 (1.536)	7.780*** (1.943)
More than one year	3.637*** (0.952)	4.634*** (1.092)	5.153 (3.002)	-0.965 (1.642)	5.585*** (1.041)
**Supervisory role (ref: No / DK)**					
Yes	1.872*** (0.502)	2.264*** (0.617)	1.372 (1.053)	1.988*** (0.522)	2.734*** (0.456)
**Occupation (ref: Elementary occupations)**					
Managers	9.208*** (1.300)	4.772** (1.601)	7.841*** (2.131)	18.95*** (1.207)	8.035*** (1.162)
Professionals	8.391*** (1.013)	2.211* (1.013)	6.187*** (1.783)	19.35*** (1.045)	7.125*** (0.948)
Technicians and associate professionals	5.815*** (0.880)	3.111*** (0.891)	7.640*** (1.527)	15.12*** (1.002)	7.611*** (0.822)
Clerical support workers	5.345*** (0.908)	2.091* (1.047)	4.794** (1.684)	10.61*** (1.111)	5.449*** (0.910)
Service and sales workers	3.466*** (0.888)	0.259 (0.841)	4.371** (1.632)	6.985*** (1.022)	3.891*** (0.821)
Skilled agricultural, forestry and fisheries	5.695** (2.114)	3.250 (2.538)	6.386* (2.914)	3.004 (3.999)	9.435*** (2.091)
Craft and related trades workers	2.806** (0.932)	2.321* (1.067)	2.317 (1.951)	7.480*** (1.016)	4.713*** (0.882)
Plant and machine operators, and assemblers	1.916 (1.098)	-0.172 (1.254)	-0.283 (1.909)	6.244*** (1.053)	2.430* (1.074)
**Constant**	68.46*** (1.031)	84.84*** (1.022)	70.22*** (1.741)	62.90*** (1.050)	75.67*** (0.955)
**Observations**	14849	10029	4461	14948	14181
**Adjusted R-squared**	0.014	0.008	0.013	0.104	0.024

Note: * p < 0.05, ** p < 0.01, *** p < 0.001. **(i)** Indicates that the categories "Part-time" and "Marginal work" are included under the same category. **(j)** Indicates that the categories "EU/EFTA" and "Non-EU/EFTA" were included under the same category. **(!)** Indicates that the coefficient is unreliable because of few observations, according to Eurostat guidelines. **na** Refers to coefficients that are not shown because of the low number of observations.

Table A6: Linear regression estimates. Difference in job satisfaction between permanent (ref.) and voluntary temporary workers with different contract durations, by country - Full models corresponding to Figure 6.

	Denmark	Sweden	Italy	Norway	Finland	Czechia	Austria	Slovakia	Spain
	B<br>(Robust SE)	B<br>(Robust SE)	B<br>(Robust SE)	B<br>(Robust SE)	B<br>(Robust SE)	B<br>(Robust SE)	B<br>(Robust SE)	B<br>(Robust SE)	B<br>(Robust SE)
**Age (ref: 35 to 44)**									
15 to 24	0.874	-0.151	1.847*	1.008	3.311**	-0.788	0.991	1.546	4.478***
	(0.892)	(0.819)	(0.750)	(0.849)	(1.008)	(0.858)	(0.774)	(1.383)	(1.028)
25 to 34	0.290	-1.774***	0.383	-0.813	0.159	0.189	0.253	-1.209	0.450
	(0.725)	(0.515)	(0.384)	(0.600)	(0.684)	(0.519)	(0.569)	(0.844)	(0.485)
45 to 54	-0.485	0.863	-0.130	1.992***	0.434	-0.583	0.920	0.159	-0.499
	(0.650)	(0.451)	(0.304)	(0.543)	(0.627)	(0.466)	(0.519)	(0.756)	(0.379)
55 to 64	0.671	2.094***	-1.915***	2.525***	0.949	-0.975	1.749**	0.606	0.975*
	(0.665)	(0.470)	(0.352)	(0.593)	(0.677)	(0.514)	(0.611)	(0.822)	(0.431)
**Gender (ref: Man)**									
Woman	0.367	-1.066**	0.824**	0.568	-0.432	-1.694***	2.031***	-3.988***	0.403
	(0.462)	(0.350)	(0.282)	(0.429)	(0.498)	(0.385)	(0.459)	(0.624)	(0.339)
**Educational level**	-0.537**	-0.505***	-0.633***	-0.612***	-0.819***	0.372*	-0.176	0.635**	-0.344**
	(0.176)	(0.132)	(0.0850)	(0.145)	(0.177)	(0.147)	(0.156)	(0.214)	(0.113)
**Working time (ref: Full-time)**									
Part-time	0.263	-1.970**	-0.946**	-1.054	0.473	1.457	0.431	5.297**	-1.688***
	(0.694)	(0.646)	(0.323)	(0.605)	(0.772)	(0.878)	(0.522)	(1.948)	(0.502)
Marginal work	1.466	-0.480	-7.735***	-2.146*	3.355*	5.612**	2.156*	-4.122	-6.806***
	(0.831)	(1.246)	(1.148)	(1.073)	(1.311)	(2.126)	(0.906)	(4.593)	(1.151)
**Nationality (ref: Local)**									
EU/EFTA	-4.133*	-2.809*	0.773	-3.970***	0.317	-1.327	-3.423***	-	1.385
	(1.720)	(1.323)	(0.599)	(0.918)	(2.033)	(1.504)	(0.741)		(1.107)
Non-EU/EFTA	-0.495	-5.159**	-0.797	-2.401	2.996	0.491	-3.254***	-	-0.603
	(1.755)	(1.753)	(0.467)	(1.473)	(2.513)	(2.182)	(0.872)		(1.053)
**Contract duration (ref: Permanent)**									
6 months or less	4.333*	3.691**	2.219	1.584	1.553	1.276	0.801	0.315	-0.384
	(1.706)	(1.277)	(2.458)	(3.369)	(1.492)	(3.434)	(1.840)	(4.242)	(2.416)
Between 7 and 12 months	2.288	0.337	0.985	na	5.515*	2.202	0.355	6.765*	2.475
	(2.144)	(2.221)	(2.887)		(2.654)	(1.842)	(1.703)	(3.373)	(4.480)
More than one year	-1.073	0.578	na	0.206	0.0647	-0.218	3.177*	3.883	na
	(1.514)	(1.621)		(3.280)	(3.452)	(1.843)	(1.614)	(4.899)	
**Supervisory role (ref: No / DK)**									
Yes	2.285***	1.110**	2.260***	1.222**	2.501***	2.402***	2.741***	3.054**	0.902*
	(0.618)	(0.360)	(0.305)	(0.436)	(0.590)	(0.512)	(0.449)	(0.948)	(0.388)
**Occupation (ref: Elementary occupations)**									
Managers	4.759**	8.130***	8.604***	6.480***	12.98***	14.01***	8.013***	23.09***	9.070***
	(1.597)	(1.203)	(1.052)	(1.391)	(1.676)	(1.227)	(1.150)	(2.037)	(1.059)
Professionals	2.225*	5.960***	9.329***	4.607***	7.613***	15.33***	7.143***	22.02***	9.317***
	(1.004)	(1.151)	(0.576)	(1.324)	(1.161)	(1.027)	(0.941)	(1.652)	(0.738)
Technicians and associate professionals	3.038***	4.907***	4.810***	4.404***	7.061***	11.66***	7.334***	17.78***	4.687***
	(0.886)	(1.097)	(0.513)	(1.298)	(1.107)	(0.909)	(0.827)	(1.513)	(0.685)
Clerical support workers	2.054*	2.388	3.524***	2.913*	4.794***	9.059***	5.494***	14.60***	4.226***
	(1.045)	(1.232)	(0.517)	(1.421)	(1.304)	(0.970)	(0.912)	(1.580)	(0.671)
Service and sales workers	0.149	0.758	2.732***	2.274	3.045**	5.625***	3.708***	8.924***	2.932***
	(0.830)	(1.108)	(0.484)	(1.271)	(1.074)	(0.943)	(0.826)	(1.492)	(0.595)
Skilled agricultural, forestry and fisheries	4.578	0.375	3.955**	7.233**	6.150*	4.494*	8.574***	11.54**	2.413
	(2.736)	(2.368)	(1.489)	(2.199)	(2.488)	(2.130)	(2.111)	(3.641)	(1.549)
Craft and related trades workers	2.613*	3.440**	3.124***	3.506*	4.285***	3.364***	4.389***	6.372***	2.649***
	(1.109)	(1.175)	(0.528)	(1.388)	(1.178)	(0.934)	(0.890)	(1.540)	(0.705)
Plant and machine operators, and assemblers	-0.382	1.625	1.538**	1.718	3.380**	0.264	2.562*	3.729*	0.144
	(1.257)	(1.253)	(0.596)	(1.460)	(1.266)	(0.919)	(1.071)	(1.520)	(0.735)
**Constant**	84.91***	85.95***	81.63***	80.51***	71.17***	72.20***	75.67***	60.21***	77.43***
	(1.021)	(1.160)	(0.520)	(1.364)	(1.245)	(0.992)	(0.950)	(1.602)	(0.658)
**Observations**	9963	15190	30515	10557	8448	13434	13969	6786	22178
**Adjusted R-squared**	0.006	0.020	0.019	0.016	0.017	0.084	0.022	0.105	0.020

Note: * $p < 0.05$, ** $p < 0.01$, *** $p < 0.001$. **(j)** Indicates that the categories "EU/EFTA" and "Non-EU/EFTA" were included under the same category. **(!)** Indicates that the coefficient is unreliable because of few observations, according to Eurostat guidelines. **na** Refers to coefficients that are not shown because of the low number of observations.

(continued)

	France	Portugal	UK	Belgium	Poland	Netherlands	Hungary
	B (Robust SE)	B (Robust SE)	B (Robust SE)	B (Robust SE)	B (Robust SE)	B (Robust SE)	B (Robust SE)
**Age (ref: 35 to 44)**							
15 to 24	1.537	2.284	0.0133	2.992***	0.224	2.131***	-2.242**
	(1.973)	(1.506)	(0.629)	(0.875)	(1.141)	(0.469)	(0.706)
25 to 34	-1.795	1.802*	-0.785	-0.235	-0.0389	-0.166	-1.700***
	(1.121)	(0.708)	(0.462)	(0.511)	(0.499)	(0.369)	(0.473)
45 to 54	-0.531	-0.927	0.824	-0.352	-0.446	0.0252	-0.657
	(0.940)	(0.555)	(0.455)	(0.481)	(0.486)	(0.327)	(0.426)
55 to 64	-0.167	-2.153**	1.310*	-0.334	1.091*	-0.140	-0.0325
	(1.114)	(0.672)	(0.519)	(0.565)	(0.514)	(0.337)	(0.497)
**Gender (ref: Man)**							
Woman	-1.403	0.446	1.797***	-0.263	-2.035***	0.494	-2.372***
	(0.822)	(0.492)	(0.364)	(0.418)	(0.399)	(0.273)	(0.361)
**Educational level**	-0.137	-1.240***	0.493***	-0.281*	0.776***	0.0481	1.052***
	(0.287)	(0.176)	(0.109)	(0.130)	(0.137)	(0.0777)	(0.171)
**Working time (ref: Full-time)**							
Part-time	0.737	-1.027	-1.627***	-0.755	1.777*	-1.064***	-2.611**
	(1.107)	(1.190)	(0.450)	(0.497)	(0.810)	(0.308)	(0.918)
Marginal work	2.254	-7.478**	1.386	1.432	-1.983	-1.289**	2.386
	(2.681)	(2.437)	(0.853)	(1.427)	(2.670)	(0.472)	(3.606)
**Nationality (ref: Local)**							
EU/EFTA	0.459	2.838	-1.427*	-0.546	1.566 (j)	-2.295*	5.694
	(2.739)	(2.711)	(0.659)	(0.678)	(3.562)	(0.924)	(3.357)
Non-EU/EFTA	-0.0445	3.259	-2.230*	-6.430***	-	-3.000*	-0.764
	(2.348)	(2.406)	(0.903)	(1.450)		(1.167)	(6.938)
**Contract duration (ref: Permanent)**							
6 months or less	-0.834	-1.580	-1.933	-2.002	-2.091	-2.629	-7.339
	(2.343)	(2.859)	(3.170)	(1.740)	(1.832)	(1.369)	(4.094)
Between 7 and 12 months	-1.967	0.911	-6.300	-2.558	-1.647	-0.935	-4.256
	(5.087)	(4.280)	(4.685)	(2.941)	(1.308)	(1.042)	(2.774)
More than one year	9.846**	na	-1.066	6.826**	-1.126	3.803	3.300
	(3.464)		(3.982)	(2.642)	(0.992)	(2.377)	(2.739)
**Supervisory role (ref: No / DK)**							
Yes	1.017	2.564***	1.412***	2.719***	1.992***	0.696*	3.023***
	(1.046)	(0.523)	(0.373)	(0.475)	(0.511)	(0.291)	(0.506)
**Occupation (ref: Elementary occupations)**							
Managers	8.608***	5.342***	10.89***	4.911***	19.45***	4.387***	15.51***
	(2.111)	(1.542)	(0.834)	(1.076)	(1.178)	(0.707)	(1.100)
Professionals	7.106***	4.672***	9.674***	6.684***	19.79***	2.445***	16.81***
	(1.778)	(1.236)	(0.773)	(0.848)	(1.015)	(0.550)	(0.970)
Technicians and associate professionals	8.411***	2.361*	7.980***	4.996***	15.93***	2.420***	13.97***
	(1.522)	(0.989)	(0.784)	(0.816)	(0.973)	(0.531)	(0.780)
Clerical support workers	5.012**	-0.198	5.483***	4.616***	10.63***	1.984***	10.69***
	(1.693)	(1.059)	(0.803)	(0.804)	(1.088)	(0.557)	(0.873)
Service and sales workers	4.979**	0.190	5.248***	3.414***	7.524***	1.687***	9.120***
	(1.620)	(0.896)	(0.734)	(0.794)	(0.986)	(0.496)	(0.768)
Skilled agricultural, forestry and fisheries	8.627**	0.938	15.41***	3.139	5.615	3.180**	5.350***
	(2.908)	(1.943)	(2.426)	(2.488)	(3.766)	(1.209)	(1.299)
Craft and related trades workers	2.408	-0.367	7.356***	2.922***	8.343***	1.372*	6.085***
	(1.961)	(1.012)	(0.922)	(0.857)	(0.991)	(0.609)	(0.764)
Plant and machine operators, and assemblers	-0.330	0.0986	4.302***	2.837**	6.574***	1.509*	2.721***
	(1.864)	(1.045)	(1.018)	(0.893)	(1.027)	(0.717)	(0.738)
**Constant**	70.22***	76.01***	64.46***	76.86***	62.52***	72.57***	67.39***
	(1.726)	(0.945)	(0.801)	(0.796)	(1.026)	(0.576)	(0.838)
**Observations**	4477	10512	28223	14533	15048	26537	17757
**Adjusted R-squared**	0.014	0.010	0.022	0.014	0.108	0.005	0.095

Note: * $p < 0.05$, ** $p < 0.01$, *** $p < 0.001$. **(j)** Indicates that the categories "EU/EFTA" and "Non-EU/EFTA" were included under the same category. **(!)** Indicates that the coefficient is unreliable because of few observations, according to Eurostat guidelines. **na** Refers to coefficients that are not shown because of the low number of observations.

III - Robustness tests

Table B1: Average marginal effects from multinomial logistic regression models. Difference in job satisfaction between permanent (ref.) and different kinds of temporary workers for the overall sample - Replications of models in Figure 1 and Table A1.

		Coef.<br>(SE)
**ref: Permanent**		
**Involuntary temporary**	Not satisfied at all + Satisfied to a small extent	0.0461***<br>(0.00206)
	Satisfied to some extent	-0.000569<br>(0.00315)
	Satisfied to a large extent	-0.0455***<br>(0.00309)
**Instrumental temporary**	Not satisfied at all + Satisfied to a small extent	-0.00496<br>(0.00296)
	Satisfied to some extent	-0.0598***<br>(0.00519)
	Satisfied to a large extent	0.0648***<br>(0.00526)
**Voluntary temporary**	Not satisfied at all + Satisfied to a small extent	-0.00331<br>(0.00332)
	Satisfied to some extent	-0.00258<br>(0.00636)
	Satisfied to a large extent	0.00590<br>(0.00629)
	**Observations**	378112

Note: p < 0.10, * p < 0.05, ** p < 0.01, *** p < 0.001.

Table B2: Average marginal effects from multinomial logistic regression models. Difference in job satisfaction between permanent (ref.) and different kinds of temporary workers, by country
Replications of models in Figure 2 and Table A2.

		**Finland**	**Luxembourg**	**Cyprus**	**Norway**	**Sweden**	**Malta**	**Denmark**	**Estonia**	**France**
		Coef. (SE)	Coef. (SE)	Coef. (SE)	Coef. (SE)	Coef. (SE)	Coef. (SE)	Coef. (SE)	Coef. (SE)	Coef. (SE)
**ref: Permanent**										
**Involuntary temporary**	Not satisfied at all + Satisfied to a small extent	-0.0264***	-0.0368	-0.0203	0.0173	0.0004	0.0145	0.0177	0.0231 (!)	0.0106
		(0.0076)	(0.0304)	(0.0135)	(0.0092)	(0.0064)	(0.0229)	(0.0117)	(0.0486)	(0.0177)
	Satisfied to some extent	-0.0302	0.0186	0.0417	-0.0425	0.0199	0.0107	-0.00231	0.0142 (!)	0.0270
		(0.0173)	(0.0460)	(0.0269)	(0.0225)	(0.0153)	(0.0376)	(0.0200)	(0.0928)	(0.0261)
	Satisfied to a large extent	0.0566***	0.0183	-0.0214	0.0253	-0.0204	-0.0252	-0.0154	-0.0373 (!)	-0.0376
		(0.0168)	(0.0433)	(0.0265)	(0.0229)	(0.0159)	(0.0411)	(0.0211)	(0.0936)	(0.0247)
**Instrumental temporary**	Not satisfied at all + Satisfied to a small extent	-0.0283	-0.120*** (!)	0.0411	-0.0058	0.0065	-0.0248	-0.0191	0.0554	-0.0388
		(0.0242)	(0.0299)	(0.0624)	(0.0177)	(0.0117)	(0.0198)	(0.0102)	(0.0315)	(0.0231)
	Satisfied to some extent	-0.172**	0.0130 (!)	-0.122	-0.128**	-0.0687**	0.0221	-0.0643**	-0.0243	-0.0471
		(0.0532)	(0.0714)	(0.0899)	(0.0443)	(0.0224)	(0.0469)	(0.0224)	(0.0519)	(0.0429)
	Satisfied to a large extent	0.201***	0.107 (!)	0.0807	0.134**	0.0622**	0.00269	0.0834***	-0.0312	0.0860*
		(0.0532)	(0.0705)	(0.0949)	(0.0454)	(0.0241)	(0.0490)	(0.0235)	(0.0534)	(0.0423)
**Voluntary temporary**	Not satisfied at all + Satisfied to a small extent	-0.0066	na	na	0.0013	-0.0148*	-0.0163	-0.0101	na	-0.0178
		(0.0160)			(0.0123)	(0.0067)	(0.0300)	(0.0121)		(0.0241)
	Satisfied to some extent	-0.0432	na	na	-0.0029	-0.0243	-0.0709	-0.0092	na	0.0275
		(0.0298)			(0.0347)	(0.0185)	(0.0488)	(0.0246)		(0.0397)
	Satisfied to a large extent	0.0499	na	na	0.0016	0.0391*	0.0871	0.0194	na	-0.0097
		(0.0285)			(0.0350)	(0.0192)	(0.0549)	(0.0257)		(0.0380)
	**Observations**	9553	2944	3763	11314	16657	4379	11020	5615	5134

Note: * p < 0.05, ** p < 0.01, *** p < 0.001. **(!)** Indicates that the coefficient is unreliable because of few observations, according to Eurostat guidelines. **na R**efers to coefficients that are not shown because of the low number of observations.

(continued)

		**Austria**	**Netherlands**	**Italy**	**Czechia**	**Greece**	**Portugal**	**Belgium**	**Spain**	**Poland**
		Coef. (SE)	Coef. (SE)	Coef. (SE)	Coef. (SE)	Coef. (SE)	Coef. (SE)	Coef. (SE)	Coef. (SE)	Coef. (SE)
**ref: Permanent**										
**Involuntary temporary**	Not satisfied at all + Satisfied to a small extent	0.0697	0.0145**	0.0132***	0.0353***	0.0302**	0.0278**	0.0313***	0.0297***	0.0574***
		(0.0360)	(0.0054)	(0.0039)	(0.0089)	(0.0106)	(0.0089)	(0.0093)	(0.0048)	(0.0071)
	Satisfied to some extent	-0.0396	0.0060	0.0282**	-0.0252	-0.0151	-0.00347	-0.0042	0.0114	0.0119
		(0.0534)	(0.0113)	(0.0088)	(0.0160)	(0.0156)	(0.0132)	(0.0163)	(0.0081)	(0.0112)
	Satisfied to a large extent	-0.0301	-0.0205	-0.0414***	-0.0102	-0.0150	-0.0243*	-0.0271	-0.0411***	-0.0693***
		(0.0556)	(0.0107)	(0.0088)	(0.0159)	(0.0159)	(0.0123)	(0.0162)	(0.0081)	(0.0107)
**Instrumental temporary**	Not satisfied at all + Satisfied to a small extent	-0.0189*	-0.0079	0.00501	na	-0.0193	-0.0158	-0.0361	0.0168	0.0284**
		(0.0090)	(0.0044)	(0.0070)		(0.0194)	(0.0172)	(0.0212)	(0.0149)	(0.0103)
	Satisfied to some extent	-0.0662***	-0.0629***	-0.0188	na	0.0076	-0.0052	-0.0535	-0.0634**	-0.0535**
		(0.0200)	(0.0120)	(0.0149)		(0.0328)	(0.0296)	(0.0580)	(0.0246)	(0.0173)
	Satisfied to a large extent	0.0851***	0.0709***	0.0138	na	0.0116	0.0211	0.0896	0.0466	0.0251
		(0.0206)	(0.0118)	(0.0152)		(0.0326)	(0.0284)	(0.0585)	(0.0250)	(0.0169)
**Voluntary temporary**	Not satisfied at all + Satisfied to a small extent	-0.0100	0.0004	-0.0127	0.0052	-0.0124	-0.0269	-0.0075	-0.0145	0.0126
		(0.0105)	(0.0076)	(0.0118)	(0.0159)	(0.0362)	(0.0206)	(0.0144)	(0.0148)	(0.0093)
	Satisfied to some extent	-0.0194	0.0471**	-0.0441	-0.0422	0.0063	0.0360	0.0139	0.0195	0.0056
		(0.0224)	(0.0167)	(0.0379)	(0.0319)	(0.0623)	(0.0429)	(0.0319)	(0.0337)	(0.0167)
	Satisfied to a large extent	0.0294	-0.0476**	0.0568	0.0369	0.0061	-0.0091	-0.0063	-0.0049	-0.0182
		(0.0228)	(0.0158)	(0.0384)	(0.0317)	(0.0646)	(0.0414)	(0.0317)	(0.0339)	(0.0161)
	**Observations**	14752	30871	35925	14530	11336	13186	15728	29157	18963

Note: * p < 0.05, ** p < 0.01, *** p < 0.001. **(!)** Indicates that the coefficient is unreliable because of few observations, according to Eurostat guidelines. **na** Refers to coefficients that are not shown because of the low number of observations.

(continued)

ref: Permanent		Germany	UK	Switzerland	Ireland	Hungary	Bulgaria	Romania	Lithuania	Slovakia
		Coef. (SE)	Coef. (SE)	Coef. (SE)	Coef. (SE)	Coef. (SE)	Coef. (SE)	Coef. (SE)	Coef. (SE)	Coef. (SE)
**Involuntary temporary**	Not satisfied at all + Satisfied to a small extent	0.0892***	0.0750***	0.0347	0.0990***	0.117***	0.150***	0.118**	0.124**	0.124***
		(0.0222)	(0.0200)	(0.0311)	(0.0193)	(0.0096)	(0.0233)	(0.0393)	(0.0405)	(0.0217)
	Satisfied to some extent	-0.0458	0.0222	0.134*	0.0651*	-0.0353**	-0.0394	0.111*	0.00161	-0.0297
		(0.0285)	(0.0249)	(0.0564)	(0.0256)	(0.0133)	(0.0315)	(0.0545)	(0.0585)	(0.0286)
	Satisfied to a large extent	-0.0434	-0.0973***	-0.169**	-0.164***	-0.0820***	-0.111***	-0.229***	-0.126*	-0.0942***
		(0.0256)	(0.0240)	(0.0575)	(0.0252)	(0.0131)	(0.0271)	(0.0429)	(0.0616)	(0.0248)
**Instrumental temporary**	Not satisfied at all + Satisfied to a small extent	-0.0162	-0.0150	-0.0451***	-0.0040	0.00133	0.0985**	na	0.0407 (!)	na
		(0.0112)	(0.0261)	(0.0076)	(0.0260)	(0.0163)	(0.0374)		(0.0431)	
	Satisfied to some extent	-0.0333	-0.0665	-0.104***	-0.128**	-0.0770*	-0.0921	na	0.0406 (!)	na
		(0.0188)	(0.0377)	(0.0225)	(0.0416)	(0.0309)	(0.0506)		(0.0739)	
	Satisfied to a large extent	0.0495**	0.0815*	0.149***	0.132**	0.0756*	-0.0064	na	-0.0813 (!)	na
		(0.0181)	(0.0392)	(0.0233)	(0.0450)	(0.0310)	(0.0438)		(0.0746)	
**Voluntary temporary**	Not satisfied at all + Satisfied to a small extent	0.0626	-0.0100	-0.0360	-0.0218	0.0379	0.158**	na	na	-0.0764***
		(0.0579)	(0.0179)	(0.0248)	(0.0168)	(0.0230)	(0.0525)			(0.0204)
	Satisfied to some extent	-0.146*	0.0689*	-0.0818	0.124**	-0.0311	-0.188**	na	na	-0.0549
		(0.0730)	(0.0274)	(0.0645)	(0.0418)	(0.0389)	(0.0610)			(0.0497)
	Satisfied to a large extent	0.0835	-0.0589*	0.118	-0.102*	-0.0067	0.0299	na	na	0.131**
		(0.0705)	(0.0264)	(0.0673)	(0.0417)	(0.0380)	(0.0526)			(0.0490)
	**Observations**	15221	28979	5770	11754	20102	10877	17325	5918	7339

Note: * p < 0.05, ** p < 0.01, *** p < 0.001. **(!)** Indicates that the coefficient is unreliable because of few observations, according to Eurostat guidelines. **na R**efers to coefficients that are not shown because of the low number of observations.

Table B3: Average marginal effects from multinomial logistic regression models. Difference in job satisfaction between permanent (ref.) and different kinds of temporary workers with different contract durations for the overall sample - Replications of models in Figure 3 and Table A3

		Involuntary temporary vs. Permanent	Instrumental temporary vs. Permanent	Voluntary temporary vs. Permanent
		Coef. (SE)	Coef. (SE)	Coef. (SE)
**ref: Permanent**				
**6 months or less**	Not satisfied at all + Satisfied to a small extent	0.0689*** (0.00344)	0.0218*** (0.00576)	0.0218*** (0.00576)
	Satisfied to some extent	-0.00775 (0.00497)	-0.0336*** (0.00946)	-0.0336*** (0.00946)
	Satisfied to a large extent	-0.0612*** (0.00489)	0.0118 (0.00942)	0.0118 (0.00942)
**Between 7 and 12 months**	Not satisfied at all + Satisfied to a small extent	0.0466*** (0.00332)	0.0165* (0.00752)	0.0165* (0.00752)
	Satisfied to some extent	-0.00113 (0.00504)	-0.0522*** (0.0126)	-0.0522*** (0.0126)
	Satisfied to a large extent	-0.0455*** (0.00497)	0.0357** (0.0127)	0.0357** (0.0127)
**More than one year**	Not satisfied at all + Satisfied to a small extent	0.0336*** (0.00431)	-0.0282*** (0.00409)	-0.0282*** (0.00409)
	Satisfied to some extent	-0.00504 (0.00686)	-0.0717*** (0.00869)	-0.0717*** (0.00869)
	Satisfied to a large extent	-0.0286*** (0.00674)	0.0999*** (0.00876)	0.0999*** (0.00876)
	**Observations**	357222	339346	337257

Note: p < 0.10, * p < 0.05, ** p < 0.01, *** p < 0.001.

Table B4: Average marginal effects from multinomial logistic regression models. Difference in job satisfaction between permanent (ref.) and involuntary temporary workers with different contract durations, by country - Replications of models in Figure 4 and Table A4.

		Luxembourg	Finland	Norway	Sweden	Denmark	France	Netherlands	Malta	Cyprus
		Coef. (SE)	Coef. (SE)	Coef. (SE)	Coef. (SE)	Coef. (SE)	Coef. (SE)	Coef. (SE)	Coef. (SE)	Coef. (SE)
**ref: Permanent**										
**6 months or less**	Not satisfied at all + Satisfied to a small extent	na	-0.0248*	0.0186	0.00552	0.0312	0.0128	0.0403*	0.0408 (!)	0.000000973
			(0.0106)	(0.0309)	(0.0107)	(0.0252)	(0.0216)	(0.0160)	(0.0478)	(0.0252)
	Satisfied to some extent	na	-0.0663**	-0.0293	0.0109	-0.0441	0.0114	-0.0331	0.0362 (!)	0.111*
			(0.0244)	(0.0718)	(0.0246)	(0.0393)	(0.0331)	(0.0285)	(0.0681)	(0.0474)
	Satisfied to a large extent	na	0.0911***	0.0106	-0.0164	0.0129	-0.0242	-0.00722	-0.0771 (!)	-0.111*
			(0.0240)	(0.0730)	(0.0258)	(0.0426)	(0.0319)	(0.0268)	(0.0758)	(0.0463)
**Between 7 and 12 months**	Not satisfied at all + Satisfied to a small extent	-0.0554 (!)	-0.0362***	-0.0110	-0.0143	-0.0110	-0.0256	0.0111	0.0264 (!)	0.00900
		(0.0516)	(0.0106)	(0.0175)	(0.0107)	(0.0180)	(0.0262)	(0.0071)	(0.0408)	(0.0206)
	Satisfied to some extent	-0.0727 (!)	-0.0552*	-0.0767	0.0319	-0.0159	0.0372	-0.0121	-0.0807 (!)	0.0847*
		(0.0855)	(0.0273)	(0.0557)	(0.0319)	(0.0375)	(0.0449)	(0.0155)	(0.0483)	(0.0339)
	Satisfied to a large extent	0.128 (!)	0.0914***	0.0877	-0.0176	0.0269	-0.0116	0.00100	0.0542 (!)	-0.0937**
		(0.0855)	(0.0269)	(0.0564)	(0.0327)	(0.0391)	(0.0433)	(0.0149)	(0.0594)	(0.0323)
**More than one year**	Not satisfied at all + Satisfied to a small extent	-0.0716*	-0.00907	0.0515**	0.00116	0.0306	0.0318	0.00363	-0.00866 (!)	-0.0299
		(0.0361)	(0.0180)	(0.0189)	(0.0100)	(0.0179)	(0.0428)	(0.0138)	(0.0347)	(0.0277)
	Satisfied to some extent	0.0251	-0.0340	-0.0201	-0.00836	0.0142	-0.0550	-0.0560	0.0769 (!)	-0.0258
		(0.0620)	(0.0358)	(0.0363)	(0.0229)	(0.0271)	(0.0583)	(0.0334)	(0.0718)	(0.0485)
	Satisfied to a large extent	0.0465	0.0431	-0.0314	0.00719	-0.0449	0.0233	0.0523	-0.0682 (!)	0.0558
		(0.0595)	(0.0346)	(0.0370)	(0.0240)	(0.0288)	(0.0570)	(0.0326)	(0.0747)	(0.0478)
	**Observations**	2872	9129	10775	15551	10194	4768	27580	4246	3724

Note: p < 0.10, * p < 0.05, ** p < 0.01, *** p < 0.001. **(!)** Indicates that the coefficient is unreliable because of few observations, according to Eurostat guidelines. **na** Refers to coefficients that are not shown because of the low number of observations.

(continued)

		Ireland	Austria	Italy	Portugal	Czechia	Spain	Belgium	Germany	Greece
		Coef. (SE)	Coef. (SE)	Coef. (SE)	Coef. (SE)	Coef. (SE)	Coef. (SE)	Coef. (SE)	Coef. (SE)	Coef. (SE)
**ref: Permanent**										
**6 months or less**	Not satisfied at all + Satisfied to a small extent	0.0670	0.0569 (!)	0.0166**	0.0464***	0.0466*	0.0521***	0.0515***	0.146**	0.086***
		(0.0455)	(0.0597)	(0.0052)	(0.0131)	(0.0212)	(0.0074)	(0.0137)	(0.0504)	(0.0170)
	Satisfied to some extent	-0.0233	-0.0138 (!)	0.0276*	-0.0069	0.0060	0.0023	0.0273	-0.137*	0.0485*
		(0.0637)	(0.0984)	(0.0115)	(0.0185)	(0.0374)	(0.0117)	(0.0219)	(0.0582)	(0.0231)
	Satisfied to a large extent	-0.0437	-0.0431 (!)	-0.0441***	-0.0395*	-0.0527	-0.0544***	-0.0788***	-0.0095	-0.1350***
		(0.0669)	(0.1020)	(0.0116)	(0.0170)	(0.0370)	(0.0117)	(0.0216)	(0.0555)	(0.0230)
**Between 7 and 12 months**	Not satisfied at all + Satisfied to a small extent	0.0658	0.0755 (!)	0.0008	0.0040	0.0489***	0.0028	0.0075	0.103**	0.0521**
		(0.0498)	(0.0546)	(0.0053)	(0.0108)	(0.0142)	(0.0091)	(0.0136)	(0.0326)	(0.0164)
	Satisfied to some extent	0.155*	0.0026 (!)	0.00826	0.00864	-0.0387	0.00301	-0.0463	-0.0295	0.0649**
		(0.0705)	(0.0810)	(0.0130)	(0.0175)	(0.0241)	(0.0170)	(0.0266)	(0.0403)	(0.0227)
	Satisfied to a large extent	-0.221***	-0.0781 (!)	-0.0091	-0.0126	-0.0102	-0.00587	0.0387	-0.0732*	-0.117***
		(0.0661)	(0.0831)	(0.0132)	(0.0166)	(0.0240)	(0.0171)	(0.0269)	(0.0354)	(0.0213)
**More than one year**	Not satisfied at all + Satisfied to a small extent	0.140**	na	-0.0009	-0.0349	0.0300*	0.0036	0.0219	0.0218	0.0136
		(0.0449)		(0.0134)	(0.0193)	(0.0124)	(0.0145)	(0.0198)	(0.0352)	(0.0239)
	Satisfied to some extent	0.0295	na	0.0251	-0.0365	-0.0187	-0.0275	-0.0472	-0.0271	-0.0393
		(0.0533)		(0.0333)	(0.0376)	(0.0224)	(0.0270)	(0.0365)	(0.0525)	(0.0348)
	Satisfied to a large extent	-0.169**	na	-0.0242	0.0714	-0.0113	0.0238	0.0253	0.00538	0.0256
		(0.0528)		(0.0334)	(0.0369)	(0.0221)	(0.0274)	(0.0372)	(0.0490)	(0.0357)
	**Observations**	11263	13558	34183	12379	14231	25517	15371	14109	11059

Note: * p < 0.05, ** p < 0.01, *** p < 0.001. **(!)** Indicates that the coefficient is unreliable because of few observations, according to Eurostat guidelines. **na** Refers to coefficients that are not shown because of the low number of observations.

(continued)

ref: Permanent		Poland Coef. (SE)	UK Coef. (SE)	Switzerland Coef. (SE)	Slovakia Coef. (SE)	Hungary Coef. (SE)	Bulgaria Coef. (SE)	Romania Coef. (SE)
**6 months or less**	Not satisfied at all + Satisfied to a small extent	0.0917***	0.135*	0.0104	0.131***	0.165***	0.204***	0.192** (!)
		(0.0144)	(0.0534)	(0.0407)	(0.0277)	(0.0156)	(0.0320)	(0.0732)
	Satisfied to some extent	-0.00394	-0.0400	0.256**	-0.0302	-0.0351	-0.00928	0.132 (!)
		(0.0220)	(0.0604)	(0.0827)	(0.0379)	(0.0207)	(0.0389)	(0.0733)
	Satisfied to a large extent	-0.0878***	-0.0947	-0.266**	-0.101**	-0.130***	-0.194***	-0.324*** (!)
		(0.0213)	(0.0600)	(0.0822)	(0.0329)	(0.0203)	(0.0288)	(0.0034)
**Between 7 and 12 months**	Not satisfied at all + Satisfied to a small extent	0.0725***	0.0247	0.0471	0.170***	0.112***	0.0716*	0.114
		(0.0109)	(0.0475)	(0.0520)	(0.0324)	(0.0111)	(0.0365)	(0.0624)
	Satisfied to some extent	0.00651	0.0420	0.0444	-0.0669	-0.0234	-0.107	0.129
		(0.0168)	(0.0648)	(0.0817)	(0.0409)	(0.0157)	(0.0568)	(0.0778)
	Satisfied to a large extent	-0.0790***	-0.0667	-0.0915	-0.103**	-0.0885***	0.0354	-0.243***
		(0.0160)	(0.0630)	(0.0871)	(0.0351)	(0.0155)	(0.0549)	(0.0534)
**More than one year**	Not satisfied at all + Satisfied to a small extent	0.0544***	0.0382	na	0.103*	0.120***	na	na
		(0.0088)	(0.0396)		(0.0499)	(0.0336)		
	Satisfied to some extent	0.0266	0.0128	na	0.0229	0.0860	na	na
		(0.0142)	(0.0512)		(0.0727)	(0.0464)		
	Satisfied to a large extent	-0.0810***	-0.0510	na	-0.126*	-0.206***	na	na
		(0.0136)	(0.0504)		(0.0617)	(0.0419)		
	**Observations**	16983	28276	5221	7199	19702	10644	17301

Note: p < 0.10, * p < 0.05, ** p < 0.01, *** p < 0.001. **(!)** Indicates that the coefficient is unreliable because of few observations, according to Eurostat guidelines. **na** Refers to coefficients that are not shown because of the low number of observations.

Table B5: Average marginal effects from multinomial logistic regression models. Difference in job satisfaction between permanent (ref.) and instrumental temporary workers with different contract durations, by country. - Replications of models in Figure 5 and Table A5.

		Ireland	Finland	Sweden	Switzerland	Portugal	UK	Greece	Spain	Italy
		Coef. (SE)	Coef. (SE)	Coef. (SE)	Coef. (SE)	Coef. (SE)	Coef. (SE)	Coef. (SE)	Coef. (SE)	Coef. (SE)
**ref: Permanent**										
**6 months or less**	Not satisfied at all + Satisfied to a small extent	na	-0.0018	-0.0109	-0.0327	-0.0036	-0.0415	-0.0218	0.0345	0.0054
			(0.0417)	(0.0109)	(0.0278)	(0.0263)	(0.0454)	(0.0282)	(0.0247)	(0.0097)
	Satisfied to some extent	na	-0.195**	-0.0683**	-0.0304	-0.0429	0.0289	0.0515	-0.0772*	0.0029
			(0.0735)	(0.0259)	(0.0670)	(0.0441)	(0.0784)	(0.0515)	(0.0361)	(0.0216)
	Satisfied to a large extent	na	0.196**	0.0793**	0.0631	0.0464	0.0126	-0.0297	0.0427	-0.0083
			(0.0742)	(0.0273)	(0.0696)	(0.0427)	(0.0787)	(0.0522)	(0.0375)	(0.0219)
**Between 7 and 12 months**	Not satisfied at all + Satisfied to a small extent	0.0483	na	0.074	-0.0226	-0.0119	na	-0.0154	0.0287	0.0123
		(0.0621)		(0.0427)	(0.0270)	(0.0270)		(0.0363)	(0.0327)	(0.0159)
	Satisfied to some extent	-0.230***	na	-0.0876	-0.0650	-0.0124	na	0.0207	-0.00315	-0.0174
		(0.0617)		(0.0528)	(0.0552)	(0.0459)		(0.0633)	(0.0496)	(0.0334)
	Satisfied to a large extent	0.181*	na	0.0136	0.0876	0.0244	na	-0.00532	-0.0255	0.00509
		(0.0794)		(0.0627)	(0.0588)	(0.0440)		(0.0628)	(0.0494)	(0.0341)
**More than one year**	Not satisfied at all + Satisfied to a small extent	-0.0083	-0.0409	na	-0.0497***	na	-0.0608	0.0441	-0.0009	-0.00702
		(0.0536)	(0.0353)		(0.0070)		(0.0371)	(0.0423)	(0.0347)	(0.0105)
	Satisfied to some extent	-0.1050	-0.1180	na	-0.118***	na	-0.0559	0.0473	-0.0869	-0.0410
		(0.0896)	(0.0941)		(0.0237)		(0.0625)	(0.0587)	(0.0553)	(0.0241)
	Satisfied to a large extent	0.1130	0.1590	na	0.168***	na	0.1170	-0.0913	0.0878	0.0480
		(0.0954)	(0.0940)		(0.0244)		(0.0643)	(0.0551)	(0.0566)	(0.0246)
	**Observations**	11143	8222	14959	5653	10691	28183	10089	22431	31582

Note: * $p < 0.05$, ** $p < 0.01$, *** $p < 0.001$. **(!)** Indicates that the coefficient is unreliable because of few observations, according to Eurostat guidelines. **na** Refers to coefficients that are not shown because of the low number of observations.

(continued)

		Germany	Denmark	France	Poland	Austria
		Coef. (SE)	Coef. (SE)	Coef. (SE)	Coef. (SE)	Coef. (SE)
**ref: Permanent**						
**6 months or less**	Not satisfied at all + Satisfied to a small extent	0.0398	0.0275	0.0129	0.0486***	0.0696*
		(0.0315)	(0.0340)	(0.0590)	(0.0146)	(0.0314)
	Satisfied to some extent	-0.0754	-0.0174	0.0353	-0.0637**	-0.00511
		(0.0442)	(0.0542)	(0.0874)	(0.0238)	(0.0465)
	Satisfied to a large extent	0.0356	-0.0101	-0.0482	0.0151	-0.0645
		(0.0423)	(0.0579)	(0.0818)	(0.0236)	(0.0474)
**Between 7 and 12 months**	Not satisfied at all + Satisfied to a small extent	0.0193	-0.0425*	na	0.0284	-0.0434*
		(0.0257)	(0.0169)		(0.0179)	(0.0175)
	Satisfied to some extent	-0.0214	-0.0753	na	-0.0282	-0.125*
		(0.0388)	(0.0551)		(0.0308)	(0.0505)
	Satisfied to a large extent	0.0021	0.118*	na	-0.0002	0.169**
		(0.0365)	(0.0564)		(0.0300)	(0.0517)
**More than one year**	Not satisfied at all + Satisfied to a small extent	-0.0400***	-0.0256*	-0.0198	0.0213	-0.0367***
		(0.0115)	(0.0111)	(0.0359)	(0.0181)	(0.0084)
	Satisfied to some extent	-0.0321	-0.0798**	-0.0898	-0.0304	-0.0757**
		(0.0224)	(0.0259)	(0.0605)	(0.0323)	(0.0233)
	Satisfied to a large extent	0.0721***	0.105***	0.1100	0.0091	0.112***
		(0.0219)	(0.0270)	(0.0598)	(0.0316)	(0.0238)
	**Observations**	14849	10029	4461	14948	14181

Note: * $p < 0.05$, ** $p < 0.01$, *** $p < 0.001$. **(!)** Indicates that the coefficient is unreliable because of few observations, according to Eurostat guidelines. **na** Refers to coefficients that are not shown because of the low number of observations.

Table B6: Average marginal effects from multinomial logistic regression models. Difference in job satisfaction between permanent (ref.) and voluntary temporary workers with different contract durations, by country. - Replications of models in Figure 6 and Table A6.

		Denmark	Sweden	Italy	Norway	Finland	Czechia	Austria	Slovakia	Spain
		Coef. (SE)	Coef. (SE)	Coef. (SE)	Coef. (SE)	Coef. (SE)	Coef. (SE)	Coef. (SE)	Coef. (SE)	Coef. (SE)
**ref: Permanent**										
**6 months or less**	Not satisfied at all + Satisfied to a small extent	-0.0294	-0.0158	-0.0177	-0.0052	-0.0090	0.051	-0.0006	0.0541	-0.0229
		(0.0170)	(0.0090)	(0.0154)	(0.0301)	(0.0199)	(0.0432)	(0.0171)	(0.0517)	(0.0227)
	Satisfied to some extent	-0.0633	-0.0533*	-0.0277	-0.0311	-0.0243	-0.137*	-0.0369	-0.167*	0.059
		(0.0422)	(0.0252)	(0.0547)	(0.0859)	(0.0371)	(0.0673)	(0.0358)	(0.0726)	(0.0542)
	Satisfied to a large extent	0.0926*	0.0691**	0.0454	0.0363	0.0333	0.086	0.0375	0.113	-0.036
		(0.0438)	(0.0263)	(0.0553)	(0.0868)	(0.0354)	(0.0693)	(0.0368)	(0.0700)	(0.0540)
**Between 7 and 12 months**	Not satisfied at all + Satisfied to a small extent	-0.016	-0.0133	-0.0116	na	-0.0128	-0.0083	0.0032	-0.0893**	0.0053
		(0.0247)	(0.0176)	(0.0191)		(0.0312)	(0.0197)	(0.0184)	(0.0297)	(0.0544)
	Satisfied to some extent	-0.0300	0.0315	-0.0186	na	-0.142*	-0.0368	-0.0276	0.0276	-0.0651
		(0.0512)	(0.0512)	(0.0640)		(0.0630)	(0.0455)	(0.0349)	(0.0740)	(0.1020)
	Satisfied to a large extent	0.0461	-0.0183	0.0302	na	0.155*	0.0452	0.0244	0.0617	0.0597
		(0.0538)	(0.0525)	(0.0637)		(0.0625)	(0.0453)	(0.0358)	(0.0710)	(0.1060)
**More than one year**	Not satisfied at all + Satisfied to a small extent	0.0045	-0.0066	na	0.0114	0.0316	0.0037	-0.0491***	-0.0402	na
		(0.0191)	(0.0128)		(0.0328)	(0.0458)	(0.0254)	(0.0135)	(0.0578)	
	Satisfied to some extent	0.0287	-0.0006	na	-0.0256	-0.074	0.011	0.0108	-0.0191	na
		(0.0347)	(0.0331)		(0.0836)	(0.0729)	(0.0491)	(0.0454)	(0.1030)	
	Satisfied to a large extent	-0.0332	0.0072	na	0.0142	0.0424	-0.0147	0.0383	0.0593	na
		(0.0360)	(0.0344)		(0.0848)	(0.0689)	(0.0477)	(0.0455)	(0.0973)	
	**Observations**	9963	15190	30515	10557	8448	13434	13969	6786	22178

Note: * p < 0.05, ** p < 0.01, *** p < 0.001. **na** Refers to coefficients that are not shown because of the low number of observations.

(continued)

		France	Portugal	UK	Belgium	Poland	Netherlands	Hungary
		Coef. (SE)	Coef. (SE)	Coef. (SE)	Coef. (SE)	Coef. (SE)	Coef. (SE)	Coef. (SE)
**ref: Permanent**								
**6 months or less**	Not satisfied at all + Satisfied to a small extent	-0.0172	-0.0068	-0.0162	0.0110	0.0085	-0.0078	0.0994*
		(0.0278)	(0.0375)	(0.0377)	(0.0219)	(0.0170)	(0.0173)	(0.0415)
	Satisfied to some extent	0.0559	0.0955	0.108	0.0439	0.0263	0.0872*	-0.144*
		(0.0472)	(0.0595)	(0.0600)	(0.0407)	(0.0358)	(0.0384)	(0.0609)
	Satisfied to a large extent	-0.0388	-0.0887	-0.0913	-0.0550	-0.0348	-0.0794*	0.0450
		(0.0448)	(0.0523)	(0.0575)	(0.0400)	(0.0349)	(0.0362)	(0.0643)
**Between 7 and 12 months**	Not satisfied at all + Satisfied to a small extent	0.0061	-0.0403	0.0659	-0.0287	0.016	0.0237	0.0146
		(0.0639)	(0.0439)	(0.0634)	(0.0260)	(0.0146)	(0.0164)	(0.0294)
	Satisfied to some extent	0.0407	0.0609	0.0235	0.1340	0.0185	-0.009	0.0809
		(0.0980)	(0.0900)	(0.0787)	(0.0687)	(0.0279)	(0.0290)	(0.0568)
	Satisfied to a large extent	-0.0468	-0.0206	-0.0894	-0.1050	-0.0344	-0.0147	-0.0956
		(0.0903)	(0.0868)	(0.0747)	(0.0672)	(0.0269)	(0.0271)	(0.0550)
**More than one year**	Not satisfied at all + Satisfied to a small extent	-0.0627	na	-0.0028	-0.0108	0.0209	-0.016	-0.0814***
		(0.0448)		(0.0474)	(0.0308)	(0.0128)	(0.0218)	(0.0020)
	Satisfied to some extent	-0.138	na	0.0092	-0.174**	0.000872	-0.0820	0.0390
		(0.0879)		(0.0677)	(0.0613)	(0.0226)	(0.0672)	(0.0943)
	Satisfied to a large extent	0.201*	na	-0.0064	0.185**	-0.0218	0.0980	0.0424
		(0.0895)		(0.0668)	(0.0640)	(0.0218)	(0.0665)	(0.0943)
	**Observations**	4477	10512	28223	14533	15048	26537	17757

Note: * p < 0.05, ** p < 0.01, *** p < 0.001. **na** Refers to coefficients that are not shown because of the low number of observations.

Table B7: Linear regression estimates. Determinants of job satisfaction - Income and household composition included as control variables, compared to reference models (Figure 2 and Table A2).

	Finland	Luxembourg	Cyprus	Malta	Denmark	Estonia	France	Austria
	B (Robust SE)	B (Robust SE)	B (Robust SE)	B (Robust SE)	B (Robust SE)	B (Robust SE)	B (Robust SE)	B (Robust SE)
**Age (ref: 35 to 44)**								
15 to 24	3.138*** (0.941)	2.843 (2.428)	-1.995 (1.899)	2.722* (1.146)	1.547 (0.873)	2.129 (1.260)	1.480 (1.632)	2.169** (0.798)
25 to 34	0.437 (0.648)	-1.267 (1.439)	-0.600 (1.178)	2.146* (1.017)	0.672 (0.723)	1.869* (0.851)	-2.233* (1.122)	0.677 (0.603)
45 to 54	0.899 (0.617)	-2.378 (1.297)	-0.477 (1.088)	1.152 (1.009)	-0.231 (0.661)	-0.220 (0.795)	1.052 (0.975)	1.046 (0.551)
55 to 64	1.897** (0.735)	0.155 (1.807)	-2.775 (1.417)	2.663* (1.102)	0.597 (0.718)	0.555 (0.965)	3.200* (1.283)	2.277** (0.708)
**Gender (ref: Man)**								
Woman	0.672 (0.498)	0.830 (1.106)	3.729*** (0.909)	1.201 (0.705)	0.195 (0.460)	1.483* (0.660)	-1.022 (0.775)	2.338*** (0.462)
**Educational level**	-1.041*** (0.170)	-0.825* (0.344)	-1.019** (0.320)	-0.208 (0.260)	-0.518** (0.176)	-0.534** (0.206)	-0.308 (0.269)	-0.356* (0.159)
**Working time (ref: Full-time)**								
Part-time	2.065** (0.772)	4.850** (1.512)	-4.337* (1.712)	1.485 (0.981)	0.286 (0.722)	1.158 (1.329)	0.251 (1.009)	1.073 (0.618)
Marginal work	5.046*** (1.325)	13.17*** (!) (2.769)	-10.38* (5.145)	-1.329 (!) (2.691)	0.664 (0.861)	-3.682 (2.891)	-0.0805 (2.458)	3.037** (1.066)
**Nationality (ref: Local)**								
EU/EFTA	0.660 (1.964)	-2.451* (1.075)	0.960 (1.261)	-2.587 (j) (2.745)	-3.585* (1.629)	-3.727 (!) (3.383)	-0.205 (2.689)	-2.978*** (0.735)
Non-EU/EFTA	2.704 (2.531)	-4.298 (2.920)	15.44*** (1.592)	-	0.00573 (1.599)	-5.658*** (0.993)	0.309 (2.094)	-3.123*** (0.862)
**Work contract (ref: Permanent)**								
Involuntary temporary	3.793*** (0.736)	3.357 (2.419)	0.902 (1.364)	-1.173 (2.033)	-1.284 (1.032)	-0.273 (!) (4.243)	-2.130 (1.366)	-4.851 (3.145)
Instrumental temporary	8.768*** (2.391)	7.752* (!) (3.750)	6.574 (6.100)	1.176 (2.204)	4.181*** (1.064)	-5.207 (3.111)	5.032* (2.092)	4.094*** (0.986)
Voluntary temporary	2.788* (1.375)	na	na	4.016 (2.488)	1.089 (1.119)	na	0.394 (1.949)	1.273 (1.071)
**Supervisory role (ref: No / DK)**								
Yes	1.647** (0.589)	0.245 (1.100)	0.867 (1.110)	2.641*** (0.700)	1.957** (0.634)	0.607 (0.691)	1.137 (1.023)	2.368*** (0.460)
**Occupation (ref: Elementary occupations)**								
Managers	9.509*** (1.700)	-0.000427 (k) (0.00304)	4.575 (2.567)	-0.00316 (k) (0.00192)	5.272** (1.625)	14.39*** (1.514)	8.650*** (2.008)	7.884*** (1.170)
Professionals	4.856*** (1.180)	-	3.044 (2.106)	-	2.616* (1.019)	14.87*** (1.426)	7.300*** (1.658)	7.157*** (0.959)
Technicians and associate professionals	5.347*** (1.090)	-	0.145 (1.894)	-	3.558*** (0.901)	11.78*** (1.422)	8.054*** (1.406)	7.371*** (0.843)
Clerical support workers	3.749** (1.240)	-	-1.057 (1.725)	-	2.485* (1.024)	9.225*** (1.575)	4.757** (1.563)	5.440*** (0.923)
Service and sales workers	2.492* (1.039)	-	-3.762* (1.622)	-	0.705 (0.808)	8.452*** (1.366)	4.450** (1.487)	4.042*** (0.822)
Skilled agricultural, forestry and fisheries	6.698** (2.368)	-	3.354 (4.648)	-	2.937 (2.798)	10.77*** (!) (2.642)	3.365 (2.800)	10.24*** (1.977)
Craft and related trades workers	2.922* (1.162)	-	-3.377 (1.829)	-	2.606* (1.068)	8.198*** (1.401)	2.544 (1.799)	4.816*** (0.883)
Plant and machine operators, and assemblers	2.417 (1.250)	-	-3.879 (2.382)	-	0.221 (1.250)	3.851** (1.404)	-0.628 (1.720)	2.577* (1.071)
**Tenure**	-0.00967*** (0.00222)	-0.0168** (0.00542)	0.0195*** (0.00458)	0.000132 (0.00285)	-0.0000319 (0.00227)	0.00364 (0.00315)	-0.0126*** (0.00345)	-0.00227 (0.00192)
**Income (deciles)**	0.816*** (0.120)	1.852*** (0.275)	1.943*** (0.240)	0.390* (0.165)	0.0113 (0.119)	1.434*** (0.137)	-	0.361** (0.116)
**Number of children in the household**	-	-	-0.461 (0.404)	0.245 (0.384)	-	0.0918 (0.270)	0.819* (0.364)	0.593** (0.224)
**Unemployed adults in the household (ref: no)**								
Yes	-	-	-1.834 (1.331)	-0.882 (2.000)	-	-3.062* (1.453)	-0.578 (1.663)	-1.558 (1.080)
**Constant**	69.35*** (1.294)	65.72*** (3.289)	69.43*** (2.037)	85.29*** (2.041)	84.29*** (1.200)	67.09*** (1.609)	71.20*** (1.726)	73.56*** (1.115)
**Observations**	9026	2527	3574	4378	10529	5615	5134	14398
**Adjusted R-squared**	0.025	0.033	0.120	0.010	0.006	0.119	0.017	0.024

Note: * p < 0.05, ** p < 0.01, *** p < 0.001. **(i)** Indicates that the categories "Part-time" and "Marginal work" are included under the same category. **(j)** Indicates that the categories "EU/EFTA" and "Non-EU/EFTA" were included under the same category. **(k)** Indicates that Occupation was included as a continuous (instead of categorical) variable. **(!)** Indicates that the coefficient is unreliable because of few observations, according to Eurostat guidelines. **na** Refers to coefficients that are not shown because of the low number of observations.

(continued)

	Netherlands	Italy	Czechia	Greece	Portugal	Belgium	Spain	Poland
	B<br>(Robust SE)	B<br>(Robust SE)	B<br>(Robust SE)	B<br>(Robust SE)	B<br>(Robust SE)	B<br>(Robust SE)	B<br>(Robust SE)	B<br>(Robust SE)
**Age (ref: 35 to 44)**								
15 to 24	1.625***<br>(0.443)	3.601***<br>(0.557)	0.660<br>(0.807)	-2.751*<br>(1.105)	3.135**<br>(1.063)	3.604***<br>(0.820)	3.451***<br>(0.715)	1.295<br>(0.822)
25 to 34	-0.212<br>(0.364)	1.225***<br>(0.356)	0.641<br>(0.537)	-2.125**<br>(0.670)	2.957***<br>(0.674)	0.192<br>(0.521)	0.628<br>(0.432)	0.445<br>(0.478)
45 to 54	0.597<br>(0.321)	-0.158<br>(0.296)	-0.600<br>(0.484)	1.078<br>(0.563)	-0.579<br>(0.569)	-0.207<br>(0.497)	-0.330<br>(0.362)	-0.923<br>(0.496)
55 to 64	1.206**<br>(0.381)	-1.790***<br>(0.386)	-0.903<br>(0.587)	0.939<br>(0.776)	-0.350<br>(0.766)	0.193<br>(0.641)	1.084*<br>(0.474)	0.0847<br>(0.589)
**Gender (ref: Man)**								
Woman	0.517*<br>(0.260)	2.088***<br>(0.264)	-1.593***<br>(0.375)	-0.919*<br>(0.454)	1.342**<br>(0.488)	0.296<br>(0.411)	1.219***<br>(0.310)	-1.702***<br>(0.370)
**Educational level**	-0.0391<br>(0.0757)	-0.725***<br>(0.0803)	0.382**<br>(0.143)	0.632***<br>(0.167)	-1.681***<br>(0.173)	-0.489***<br>(0.133)	-0.729***<br>(0.104)	0.659***<br>(0.129)
**Working time (ref: Full-time)**								
Part-time	-1.235***<br>(0.316)	1.216***<br>(0.317)	1.316<br>(0.824)	-9.008***<br>(0.599)	-0.626<br>(1.018)	0.621<br>(0.539)	-0.154<br>(0.475)	1.205<br>(0.716)
Marginal work	-1.431**<br>(0.478)	-5.068***<br>(0.974)	4.821*<br>(1.959)	-24.29***<br>(2.006)	-5.192*<br>(2.212)	1.111<br>(1.359)	-6.038***<br>(0.921)	-2.129<br>(2.082)
**Nationality (ref: Local)**								
EU/EFTA	-2.960**<br>(0.907)	0.567<br>(0.547)	-1.660<br>(1.589)	-1.139<br>(1.712)	1.064<br>(2.406)	-0.463<br>(0.649)	1.070<br>(0.948)	2.650 (j)<br>(2.814)
Non-EU/EFTA	-4.224***<br>(1.046)	-0.621<br>(0.428)	1.416<br>(2.087)	-2.227*<br>(0.901)	1.486<br>(2.028)	-4.583***<br>(1.272)	-1.943*<br>(0.865)	-
**Work contract (ref: Permanent)**								
Involuntary temporary	-1.313**<br>(0.465)	-1.376***<br>(0.386)	-2.061**<br>(0.746)	-2.260**<br>(0.785)	-1.978**<br>(0.685)	-2.023**<br>(0.770)	-2.699***<br>(0.388)	-5.209***<br>(0.549)
Instrumental temporary	2.423***<br>(0.459)	1.077<br>(0.641)	na	1.217<br>(1.623)	2.047<br>(1.459)	4.815*<br>(2.131)	2.049<br>(1.178)	-0.897<br>(0.906)
Voluntary temporary	-1.696**<br>(0.629)	2.826<br>(1.639)	1.429<br>(1.330)	1.104<br>(2.865)	1.298<br>(2.239)	0.233<br>(1.376)	0.835<br>(1.561)	-0.768<br>(0.771)
**Supervisory role (ref: No / DK)**								
Yes	0.544<br>(0.282)	1.322***<br>(0.295)	2.349***<br>(0.500)	0.244<br>(0.676)	1.897***<br>(0.518)	2.270***<br>(0.471)	0.309<br>(0.375)	2.074***<br>(0.485)
**Occupation (ref: Elementary occupations)**								
Managers	4.111***<br>(0.685)	5.325***<br>(1.059)	14.91***<br>(1.197)	18.00***<br>(1.854)	2.721<br>(1.522)	3.533**<br>(1.079)	7.348***<br>(1.012)	19.85***<br>(1.063)
Professionals	2.461***<br>(0.514)	6.694***<br>(0.558)	16.25***<br>(0.992)	20.54***<br>(1.017)	2.157<br>(1.182)	5.767***<br>(0.845)	8.000***<br>(0.676)	20.29***<br>(0.899)
Technicians and associate professionals	2.661***<br>(0.491)	3.106***<br>(0.484)	12.69***<br>(0.877)	16.57***<br>(1.026)	-0.0147<br>(0.970)	4.358***<br>(0.809)	3.887***<br>(0.621)	15.92***<br>(0.849)
Clerical support workers	1.501**<br>(0.514)	2.640***<br>(0.475)	10.19***<br>(0.933)	11.89***<br>(0.900)	-1.845<br>(0.990)	4.186***<br>(0.782)	3.725***<br>(0.597)	11.27***<br>(0.939)
Service and sales workers	1.591***<br>(0.454)	2.725***<br>(0.431)	6.855***<br>(0.902)	6.373***<br>(0.827)	-1.348<br>(0.820)	2.962***<br>(0.760)	2.386***<br>(0.508)	7.688***<br>(0.830)
Skilled agricultural, forestry and fisheries	3.726***<br>(1.131)	3.302**<br>(1.127)	6.618**<br>(2.048)	-2.125<br>(2.578)	0.642<br>(1.909)	2.355<br>(2.629)	3.225*<br>(1.299)	4.724<br>(3.219)
Craft and related trades workers	1.197*<br>(0.562)	2.394***<br>(0.478)	4.295***<br>(0.900)	6.163***<br>(1.010)	-1.285<br>(0.938)	2.654**<br>(0.833)	2.140***<br>(0.603)	8.518***<br>(0.838)
Plant and machine operators, and assemblers	1.699**<br>(0.642)	0.586<br>(0.542)	1.373<br>(0.882)	5.535***<br>(1.063)	-1.011<br>(0.956)	2.871***<br>(0.857)	-0.686<br>(0.638)	6.599***<br>(0.876)
**Tenure**	-0.00710***<br>(0.00103)	-0.00315**<br>(0.00116)	0.00395*<br>(0.00177)	0.0383***<br>(0.00240)	-0.00708**<br>(0.00228)	-0.00388*<br>(0.00177)	-0.00271<br>(0.00146)	0.00708***<br>(0.00172)
**Income (deciles)**	0.0220<br>(0.0692)	1.056***<br>(0.0595)	-	-	0.928***<br>(0.119)	0.609***<br>(0.101)	0.843***<br>(0.0780)	-
**Number of children in the household**	0.211<br>(0.110)	0.264*<br>(0.124)	0.292<br>(0.206)	0.775***<br>(0.235)	0.764**<br>(0.262)	0.183<br>(0.173)	0.239<br>(0.156)	0.422*<br>(0.191)
**Unemployed adults in the household (ref: no)**								
Yes	0.538<br>(0.613)	0.312<br>(0.403)	-2.185<br>(1.146)	-1.206*<br>(0.586)	1.142<br>(0.840)	-1.259<br>(0.864)	-1.661***<br>(0.415)	0.569<br>(0.886)
**Constant**	73.36***<br>(0.669)	76.06***<br>(0.566)	70.29***<br>(1.000)	59.84***<br>(1.010)	73.05***<br>(1.034)	74.50***<br>(0.890)	74.53***<br>(0.688)	61.34***<br>(0.949)
**Observations**	30293	35925	14530	11336	11912	15728	29157	18963
**Adjusted R-squared**	0.008	0.034	0.088	0.203	0.018	0.017	0.036	0.122

Note: * p < 0.05, ** p < 0.01, *** p < 0.001. **(i)** Indicates that the categories "Part-time" and "Marginal work" are included under the same category. **(j)** Indicates that the categories "EU/EFTA" and "Non-EU/EFTA" were included under the same category. **(k)** Indicates that Occupation was included as a continuous (instead of categorical) variable. **(!)** Indicates that the coefficient is unreliable because of few observations, according to Eurostat guidelines. **na** Refers to coefficients that are not shown because of the low number of observations.

(continued)

	Germany	UK	Switzerland	Ireland	Hungary	Bulgaria	Romania	Lithuania	Slovakia
	B (Robust SE)	B (Robust SE)	B (Robust SE)	B (Robust SE)	B (Robust SE)	B (Robust SE)	B (Robust SE)	B (Robust SE)	B (Robust SE)
**Age (ref: 35 to 44)**									
15 to 24	2.691** (0.868)	-0.532 (0.644)	0.633 (1.258)	-1.313 (0.921)	-0.833 (0.707)	1.809 (1.257)	-0.536 (0.809)	1.258 (1.375)	3.556** (1.350)
25 to 34	1.515* (0.627)	-1.159* (0.475)	0.1000 (0.949)	0.0947 (0.634)	-0.127 (0.487)	1.745* (0.686)	0.673 (0.441)	-0.0789 (0.900)	0.349 (0.873)
45 to 54	-0.304 (0.583)	1.594*** (0.470)	0.427 (0.844)	1.474* (0.610)	-0.470 (0.435)	1.376* (0.598)	0.297 (0.386)	-1.197 (0.792)	0.189 (0.795)
55 to 64	-0.0424 (0.678)	2.959*** (0.575)	-0.0142 (0.994)	3.053*** (0.794)	-0.132 (0.536)	1.593* (0.673)	-0.00307 (0.517)	-1.375 (0.915)	0.687 (0.940)
**Gender (ref: Man)**									
Woman	0.0714 (0.471)	1.900*** (0.360)	-0.758 (0.700)	1.241* (0.503)	-1.052** (0.361)	-0.113 (0.475)	-1.102*** (0.323)	-1.543* (0.627)	-3.064*** (0.615)
**Educational level**	-0.613*** (0.163)	0.409*** (0.108)	-0.389 (0.214)	0.217 (0.166)	0.467** (0.174)	0.265 (0.188)	0.518*** (0.144)	1.337*** (0.241)	0.796*** (0.214)
**Working time (ref: Full-time)**									
Part-time	2.073*** (0.606)	-1.943*** (0.451)	3.923*** (1.059)	-1.126 (0.586)	-2.077* (0.863)	-10.75***(i) (1.761)	0.285 (i) (1.579)	-2.876* (1.157)	-5.865*** (1.711)
Marginal work	5.254*** (0.849)	0.304 (0.843)	6.499*** (1.462)	0.367 (1.260)	-1.410 (3.206)	-	-	-9.165** (3.212)	-20.31*** (3.350)
**Nationality (ref: Local)**									
EU/EFTA	0.413 (0.956)	-1.967** (0.651)	-3.217*** (0.691)	-5.086*** (0.811)	7.488* (2.915)	-	-	-	-
Non-EU/EFTA	-1.275 (0.932)	-2.897** (0.893)	-4.156** (1.296)	-3.091* (1.324)	0.527 (6.229)	-	-	-	-
**Work contract (ref: Permanent)**									
Involuntary temporary	-5.179*** (1.500)	-8.020*** (1.575)	-6.500* (3.036)	-9.532*** (1.354)	-9.517*** (0.742)	-12.48*** (1.496)	-13.77*** (2.587)	-11.84** (3.848)	-14.69*** (1.586)
Instrumental temporary	3.382*** (0.901)	2.614 (2.299)	9.787*** (1.310)	5.063* (2.258)	2.470 (1.594)	-3.803 (2.537)	na	-4.276 (!) (4.131)	na
Voluntary temporary	0.266 (3.934)	-1.437 (1.508)	6.849* (2.921)	-1.632 (1.905)	-2.597 (2.056)	-8.132* (3.478)	na	na	12.50*** (2.635)
**Supervisory role (ref: No / DK)**									
Yes	1.507** (0.511)	1.618*** (0.373)	0.250 (0.713)	0.103 (0.536)	2.595*** (0.508)	4.642*** (0.776)	1.586** (0.595)	2.164* (0.850)	2.629** (0.938)
**Occupation (ref: Elementary occupations)**									
Managers	8.397*** (1.322)	11.08*** (0.823)	3.380 (1.966)	10.81*** (1.267)	14.36*** (1.090)	14.98*** (1.512)	14.18*** (1.358)	12.51*** (1.515)	25.02*** (1.958)
Professionals	7.537*** (1.027)	10.03*** (0.760)	3.309 (1.784)	11.54*** (1.094)	15.68*** (0.961)	15.66*** (1.178)	13.88*** (0.873)	12.32*** (1.305)	23.45*** (1.581)
Technicians and associate professionals	5.731*** (0.893)	8.338*** (0.772)	3.242 (1.722)	8.918*** (1.085)	13.02*** (0.755)	11.67*** (1.106)	11.83*** (0.839)	12.45*** (1.376)	19.49*** (1.437)
Clerical support workers	5.331*** (0.917)	5.595*** (0.790)	3.160 (1.858)	8.238*** (1.104)	10.30*** (0.818)	12.12*** (1.107)	8.403*** (0.886)	9.427*** (1.627)	16.59*** (1.483)
Service and sales workers	3.845*** (0.891)	5.106*** (0.721)	-0.273 (1.762)	4.616*** (1.016)	9.569*** (0.706)	3.401*** (0.882)	5.496*** (0.712)	4.753*** (1.276)	11.65*** (1.395)
Skilled agricultural, forestry and fisheries	7.284*** (2.166)	14.91*** (2.479)	-1.035 (3.221)	9.376*** (2.054)	7.366*** (1.177)	1.068 (1.956)	5.706** (1.867)	3.188 (2.311)	12.55*** (3.634)
Craft and related trades workers	3.135*** (0.941)	7.422*** (0.907)	2.375 (1.850)	9.343*** (1.166)	6.258*** (0.705)	4.186*** (0.908)	3.879*** (0.693)	4.462*** (1.300)	8.618*** (1.442)
Plant and machine operators, and assemblers	1.840 (1.094)	3.975*** (1.001)	-0.174 (2.388)	4.552*** (1.310)	3.086*** (0.683)	4.300*** (0.903)	3.333*** (0.713)	4.227** (1.303)	5.970*** (1.420)
**Tenure**	-0.00472* (0.00184)	-0.0136*** (0.00180)	0.000991 (0.00348)	0.00178 (0.00229)	0.0151*** (0.00165)	0.00839*** (0.00253)	0.0151*** (0.00187)	0.0154*** (0.00345)	0.0113*** (0.00281)
**Income (deciles)**	0.737*** (0.110)	-	0.936*** (0.213)	-	0.625*** (0.0770)	1.594*** (0.0938)	0.534*** (0.0567)	0.825*** (0.106)	-
**Number of children in the household**	-0.0412 (0.227)	0.470** (0.176)	-	0.115 (0.199)	0.593*** (0.169)	0.766* (0.302)	0.625*** (0.187)	0.961** (0.337)	1.183*** (0.316)
**Unemployed adults in the household (ref: no)**									
Yes	-1.475 (1.406)	-0.482 (0.955)	-	-1.339 (1.151)	0.0699 (0.937)	-2.108 (1.102)	-1.390 (0.727)	0.714 (1.204)	-1.911 (1.063)
**Constant**	65.24*** (1.165)	65.39*** (0.833)	79.24*** (2.156)	71.84*** (1.161)	63.27*** (0.846)	49.65*** (1.101)	61.05*** (0.838)	64.38*** (1.571)	54.76*** (1.580)
**Observations**	14864	28925	5500	11754	20102	10181	17325	5622	7339
**Adjusted R-squared**	0.018	0.026	0.028	0.043	0.146	0.205	0.100	0.152	0.190

Note: * p < 0.05, ** p < 0.01, *** p < 0.001. **(i)** Indicates that the categories "Part-time" and "Marginal work" are included under the same category. **(j)** Indicates that the category "EU/EFTA" and "Non-EU/EFTA" were included under the same categories. **(k)** Indicates that Occupation was included as a continuous (instead of categorical) variable. **(!)** Indicates that the coefficient is unreliable because of few observations, according to Eurostat guidelines. **na** Refers to coefficients that are not shown because of the low number of observations.

IV- Descriptive statistics of the sample

Table C1: Descriptive statistics of the sample - Correspondence with samples from Figure 1, Table A1 and Table B1.

	Mean / Percentage *(SD)*
**Age**	
15 to 24	8.49
25 to 34	19.83
35 to 44	25.54
45 to 54	27.55
55 to 64	18.60
**Gender**	
Man	50.08
Woman	49.92
**Educational level**	3.97
	(1.84)
**Working time**	
Full-time	81.26
Part-time	14.97
Marginal work	3.78
**Nationality**	
Native	93.44
EU/EFTA	3.70
Non-EU/EFTA	2.86
**Work contract**	
Permanent	87.79
Involuntary temporary	8.01
Instrumental temporary	2.52
Voluntary temporary	1.68
**Supervisory role**	
No	78.47
Yes	21.53
**Occupation**	
Managers	4.78
Professionals	20.58
Technicians and associate professionals	15.52
Clerical support workers	10.39
Service and sales workers	17.88
Skilled agricultural, forestry and fisheries	0.96
Craft and related trades workers	11.13
Plant and machine operators, and assemblers	8.80
Elementary	9.95
**Tenure (in months)**	122.22
	(120.69)
**Country**	
Austria	3.90
Belgium	4.16
Bulgaria	2.88
Switzerland	1.53
Cyprus	1.00
Czechia	3.84
Germany	4.03
Denmark	2.91
Estonia	1.49
Spain	7.71
Finland	2.53
France	1.36
Greece	3.00
Hungary	5.32
Ireland	3.11
Italy	9.50
Lithuania	1.57
Luxembourg	0.78
Malta	1.16
Netherlands	8.16
Norway	2.99
Poland	5.02
Portugal	3.49
Romania	4.58
Sweden	4.41
Slovak Republic	1.94
United Kingdom	7.66

Table C2: Descriptive statistics of the sample - Correspondence with samples from Figure 2, Table A2 and Table B2.

	Finland	Luxembourg	Cyprus	Norway	Sweden	Malta	Denmark	Estonia	France
	Mean / Percentage *(SD)*	Mean / Percentage *(SD)*	Mean / Percentage *(SD)*	Mean / Percentage *(SD)*	Mean / Percentage *(SD)*	Mean / Percentage *(SD)*	Mean / Percentage *(SD)*	Mean / Percentage *(SD)*	Mean / Percentage *(SD)*
**Age**									
15 to 24	10.13	6.45	6.35	10.99	8.48	14.43	20.10	7.87	9.04
25 to 34	21.42	22.79	24.18	21.70	20.25	22.77	17.11	19.55	18.52
35 to 44	23.05	27.48	28.09	22.85	24.01	24.71	19.36	23.97	26.28
45 to 54	24.90	31.32	25.11	25.64	26.11	21.33	23.89	26.79	29.68
55 to 64	20.50	11.96	16.26	18.83	21.16	16.76	19.55	21.82	16.48
**Gender**									
Man	47.85	51.94	47.54	50.96	49.17	56.66	48.21	46.34	44.99
Woman	52.15	48.06	52.46	49.04	50.83	43.34	51.79	53.66	55.01
**Educational level**	4.33	4.21	4.19	4.18	4.35	3.50	3.99	4.26	3.92
	(1.77)	*(2.11)*	*(1.87)*	*(1.77)*	*(1.75)*	*(1.74)*	*(1.87)*	*(1.80)*	*(1.72)*
**Working time**									
Full-time	84.10	80.43	90.41	79.30	86.26	82.96	72.51	91.65	81.22
Part-time	11.65	16.71	8.26	14.76	10.53	15.25	13.10	6.95	16.38
Marginal work	4.25	2.85 (!)	1.33	5.94	3.21	1.78 (!)	14.38	1.41	2.40
**Nationality**									
Native	96.83	59.82	78.63	91.54	96.15	98.06	95.60	87.98	96.07
EU/EFTA	1.81	36.99	12.60	6.05	2.25	1.94	2.34	0.62 (!)	1.71
Non-EU/EFTA	1.36	3.19	8.77	2.41	1.60	-	2.06	11.40	2.22
**Work contract**									
Permanent	85.14	93.04	83.87	92.53	88.01	94.11	86.96	97.81	83.66
Involuntary temporary	10.58	4.55	15.09	4.52	6.12	2.85	5.54	0.46 (!)	9.21
Instrumental temporary	0.94	1.83 (!)	0.66	1.01	1.90	1.92	4.05	1.44	3.58
Voluntary temporary	3.34	na	na	1.94	3.97	1.12	3.45	na	3.54
**Supervisory role**									
No	81.28	67.22	80.65	64.13	67.19	63.78	84.84	74.19	81.55
Yes	18.72	32.78	19.35	35.87	32.81	36.22	15.16	25.81	18.45
**Occupation**									
Managers	2.92	404.52 (k)	4.12	8.83	6.33	443.89 (k)	2.45	9.23	6.90
		(236.01)				*(243.02)*			
Professionals	25.51		17.41	27.81	30.26		27.79	19.57	16.61
Technicians and associate professionals	20.21	-	13.93	18.20	20.10	-	17.31	14.07	22.48
Clerical support workers	6.97	-	14.35	6.02	6.80	-	7.76	6.13	11.04
Service and sales workers	19.46	-	18.76	20.16	17.96	-	22.01	13.48	14.45
Skilled agricultural, forestry and fisheries	1.12	-	0.64	0.85	0.70	-	0.88	0.94 (!)	2.16
Craft and related trades workers	9.74	-	9.70	9.09	8.43	-	6.72	12.18	7.62
Plant and machine operators, and assemblers	7.46	-	4.41	5.86	5.66	-	4.11	14.80	7.73
Elementary	6.61	-	16.69	3.18	3.76	-	10.96	9.60	11.01
**Tenure (in months)**	117.02	127.69	112.47	108.45	114.60	117.88	85.19	97.83	138.72
	(125.04)	*(118.22)*	*(113.91)*	*(111.71)*	*(125.71)*	*(126.14)*	*(106.46)*	*(101.55)*	*(127.96)*

Note: (i) Indicates that the categories "Part-time" and "Marginal work" are included under the same category. **(j)** Indicates that the categories "EU/EFTA" and "Non-EU/EFTA" were included under the same category. **(k)** Indicates that Occupation was included as a continuous (instead of categorical) variable. **(!)** Indicates that the coefficient is unreliable because of few observations, according to Eurostat guidelines. **na** Refers to frequencies that are not shown because of the low number of observations.

(continued)

	Austria	Netherlands	Italy	Czechia	Greece	Portugal	Belgium	Spain	Poland
	Mean / Percentage *(SD)*	Mean / Percentage *(SD)*	Mean / Percentage *(SD)*	Mean / Percentage *(SD)*	Mean / Percentage *(SD)*	Mean / Percentage *(SD)*	Mean / Percentage *(SD)*	Mean / Percentage *(SD)*	Mean / Percentage *(SD)*
**Age**									
15 to 24	12.22	13.57	4.84	6.52	5.05	6.55	6.53	5.14	6.86
25 to 34	21.41	18.24	15.63	19.46	20.34	16.62	23.47	17.55	24.78
35 to 44	22.74	18.50	26.95	27.51	31.02	29.80	26.15	29.89	27.47
45 to 54	29.56	26.68	32.10	27.21	30.97	28.94	27.49	29.46	22.47
55 to 64	14.06	23.02	20.48	19.30	12.62	18.09	16.35	17.96	18.42
**Gender**									
Man	50.58	50.75	52.14	50.58	52.83	46.92	49.51	50.57	47.26
Woman	49.42	49.25	47.86	49.42	47.17	53.08	50.49	49.43	52.74
**Educational level**	3.77	4.09	3.46	3.79	4.03	3.23	4.30	4.03	4.43
	(1.56)	*(1.91)*	*(1.81)*	*(1.62)*	*(1.82)*	*(2.22)*	*(1.99)*	*(2.03)*	*(1.90)*
**Working time**									
Full-time	73.74	61.92	73.78	93.75	80.68	91.54	75.41	82.09	92.59
Part-time	21.17	27.81	23.71	5.42	17.86	6.40	22.25	14.34	6.46
Marginal work	5.09	10.27	2.51	0.83	1.46	2.06	2.34	3.57	0.95
**Nationality**									
Native	85.59	97.52	88.55	98.25	91.60	97.94	89.85	94.33	99.61
EU/EFTA	8.58	1.49	3.89	1.13	1.49	0.62	7.63	2.29	0.39 (j)
Non-EU/EFTA	5.84	0.99	7.56	0.63	6.91	1.43	2.52	3.38	-
**Work contract**									
Permanent	91.36	84.42	84.53	90.47	87.09	78.91	90.60	75.63	73.87
Involuntary temporary	0.54	6.66	11.52	7.47	10.47	17.56	7.13	22.12	15.69
Instrumental temporary	4.77	6.19	3.51	na	1.91	2.46	0.47	1.49	4.96
Voluntary temporary	3.33	2.73	0.44	1.98	0.53	1.08	1.80	0.76	5.48
**Supervisory role**									
No	75.64	77.96	80.18	82.77	88.36	73.36	78.94	82.97	81.80
Yes	24.36	22.04	19.82	17.23	11.64	26.64	21.06	17.03	18.20
**Occupation**									
Managers	4.58	5.56	1.29	3.89	1.38	3.03	6.17	2.49	5.92
Professionals	15.92	28.21	13.78	14.03	20.84	18.28	23.61	18.85	22.59
Technicians and associate professionals	20.04	17.80	17.26	18.18	9.62	13.04	15.09	11.54	14.94
Clerical support workers	10.71	10.91	15.25	10.87	15.16	9.16	13.10	12.01	7.89
Service and sales workers	19.27	17.38	17.99	14.52	23.54	19.24	13.11	20.45	14.35
Skilled agricultural, forestry and fisheries	0.75	0.95	0.88	0.80	0.74	2.05	0.50	1.13	0.36
Craft and related trades workers	13.67	7.19	11.80	14.91	9.02	11.91	9.39	10.47	14.81
Plant and machine operators, and assemblers	5.97	4.37	8.21	16.42	7.36	8.88	7.48	8.46	10.81
Elementary	9.10	7.63	13.54	6.38	12.35	14.41	11.55	14.60	8.33
**Tenure (in months)**	122.09	133.98	152.72	122.05	129.16	146.66	135.53	137.94	123.60
	(124.77)	*(133.25)*	*(130.71)*	*(114.99)*	*(115.01)*	*(131.10)*	*(126.88)*	*(130.10)*	*(122.94)*

Note: (i) Indicates that the categories "Part-time" and "Marginal work" are included under the same category. **(j)** Indicates that the categories "EU/EFTA" and "Non-EU/EFTA" were included under the same category. **(k)** Indicates that Occupation was included as a continuous (instead of categorical) variable. **(!)** Indicates that the coefficient is unreliable because of few observations, according to Eurostat guidelines. **na** Refers to frequencies that are not shown because of the low number of observations.

(continued)

	Germany	UK	Switzerland	Ireland	Hungary	Bulgaria	Romania	Lithuania	Slovakia
	Mean / Percentage *(SD)*	Mean / Percentage *(SD)*	Mean / Percentage *(SD)*	Mean / Percentage *(SD)*	Mean / Percentage *(SD)*	Mean / Percentage *(SD)*	Mean / Percentage *(SD)*	Mean / Percentage *(SD)*	Mean / Percentage *(SD)*
**Age**									
15 to 24	9.80	11.17	13.97	9.96	7.56	4.51	4.62	5.68	6.74
25 to 34	19.74	22.59	17.40	23.92	19.83	18.06	20.74	16.49	20.17
35 to 44	19.83	23.70	23.40	29.52	28.39	26.94	30.15	20.78	26.86
45 to 54	29.08	25.39	27.23	22.55	26.61	27.53	30.94	31.50	26.03
55 to 64	21.54	17.15	18.01	14.05	17.62	22.97	13.55	25.55	20.21
**Gender**									
Man	50.84	48.10	50.07	47.65	52.78	51.35	53.92	44.19	49.30
Woman	49.16	51.90	49.93	52.35	47.22	48.65	46.08	55.81	50.70
**Educational level**	3.94	4.04	4.27	4.52	3.58	3.94	3.84	4.59	3.86
	(1.66)	*(1.75)*	*(2.00)*	*(1.68)*	*(1.48)*	*(1.84)*	*(1.67)*	*(1.61)*	*(1.70)*
**Working time**									
Full-time	73.04	74.08	74.45	73.63	94.50	97.43	98.89	91.31	92.68
Part-time	19.18	21.23	17.90	22.42	5.10	2.57 (i)	1.11 (i)	7.52	6.27
Marginal work	7.78	4.69	7.64	3.96	0.40	-	-	1.17	1.05
**Nationality**									
Native	91.80	90.16	65.49	87.57	99.67	-	-	99.56	-
EU/EFTA	3.94	6.68	27.56	9.72	0.20	-	-	0.44 (j) (!)	-
Non-EU/EFTA	4.26	3.16	6.95	2.71	0.13	-	-	-	-
**Work contract**									
Permanent	90.60	96.83	89.13	94.20	87.53	94.75	99.43	98.07	90.75
Involuntary temporary	2.11	1.41	1.35	3.52	10.48	3.64	0.43	1.12	7.39
Instrumental temporary	6.98	0.56	8.84	0.89	1.18	0.95	na	0.68 (!)	na
Voluntary temporary	0.31	1.21	0.68	1.39	0.81	0.66	na	na	1.72
**Supervisory role**									
No	76.58	63.41	67.38	70.48	87.25	89.30	92.61	85.82	89.03
Yes	23.42	36.59	32.62	29.52	12.75	10.70	7.39	14.18	10.97
**Occupation**									
Managers	3.98	10.86	9.25	6.87	3.71	3.42	1.62	8.09	3.15
Professionals	16.73	24.74	24.32	23.47	10.99	15.66	19.41	23.47	11.99
Technicians and associate professionals	23.40	13.37	21.01	13.31	13.78	9.43	7.86	9.43	15.27
Clerical support workers	14.20	11.17	8.49	11.46	7.56	6.75	6.15	5.10	10.78
Service and sales workers	13.94	19.77	16.85	21.61	13.70	21.08	19.25	13.40	18.57
Skilled agricultural, forestry and fisheries	0.74	0.45	1.21	0.88	2.15	1.64	0.74	2.01	0.63
Craft and related trades workers	12.82	6.22	11.18	8.36	15.38	14.27	20.89	13.72	12.89
Plant and machine operators, and assemblers	6.46	4.79	3.60	5.44	18.35	15.42	16.04	13.84	16.72
Elementary	7.73	8.64	4.09	8.60	14.38	12.34	8.03	10.93	10.00
**Tenure (in months)**	136.01	99.66	99.48	110.80	103.51	107.51	107.03	87.84	121.51
	(131.03)	*(104.71)*	*(106.44)*	*(111.21)*	*(110.90)*	*(94.68)*	*(89.35)*	*(83.73)*	*(115.42)*

Note: (i) Indicates that the categories "Part-time" and "Marginal work" are included under the same category. **(j)** Indicates that the categories "EU/EFTA" and "Non-EU/EFTA" were included under the same category. **(k)** Indicates that Occupation was included as a continuous (instead of categorical) variable. **(!)** Indicates that the coefficient is unreliable because of few observations, according to Eurostat guidelines. **na** Refers to frequencies that are not shown because of the low number of observations.

Table C3: Descriptive statistics of the sample - Correspondence with samples from Figure 3, Table A3 and Table B3.

	Model: Involuntary temporary vs. Permanent	Model: Instrumental temporary vs. Permanent	Model: Voluntary temporary vs. Permanent
	Mean / Percentage	Mean / Percentage	Mean / Percentage
	(SD)	*(SD)*	*(SD)*
**Age**			
15 to 24	6.81	7.04	6.56
25 to 34	19.48	18.97	18.81
35 to 44	26.12	25.96	26.09
45 to 54	28.41	28.62	28.82
55 to 64	19.18	19.42	19.72
**Gender**			
Man	50.05	50.42	50.26
Woman	49.95	49.58	49.74
**Educational level**	4.00	4.02	4.02
	(1.84)	*(1.83)*	*(1.83)*
**Working time**			
Full-time	82.19	82.92	82.43
Part-time	14.49	13.95	14.19
Marginal work	3.32	3.13	3.38
**Nationality**			
Native	93.53	93.69	93.75
EU/EFTA	3.69	3.71	3.70
Non-EU/EFTA	2.77	2.60	2.55
**Work contract**			
Permanent	92.92	97.82	98.42
6 months or less	2.87	0.78	0.62
Between 7 and 12 months	2.75	0.43	0.49
More than one year	1.45	0.97	0.46
**Supervisory role**			
No	77.68	76.92	76.75
Yes	22.32	23.08	23.25
**Occupation**			
Managers	4.97	5.18	5.24
Professionals	20.83	21.13	21.22
Technicians and associate professionals	15.72	16.21	16.15
Clerical support workers	10.47	10.58	10.54
Service and sales workers	17.46	17.26	17.33
Skilled agricultural, forestry and fisheries	0.93	0.87	0.86
Craft and related trades workers	11.02	11.29	11.06
Plant and machine operators, and assemblers	8.92	8.89	8.89
Elementary	9.68	8.59	8.70
**Country**			
Austria	3.80	4.18	4.14
Belgium	4.30	4.22	4.31
Bulgaria	2.98	3.07	3.08
Switzerland	1.46	1.67	1.54
Cyprus	1.04	0.94	0.94
Czechia	3.98	3.88	3.98
Germany	3.95	4.38	4.10
Denmark	2.85	2.96	2.95
Estonia	1.54	1.64	1.63
Spain	7.14	6.61	6.58
Finland	2.56	2.42	2.50
France	1.33	1.31	1.33
Greece	3.10	2.97	2.94
Hungary	5.52	5.26	5.27
Ireland	3.15	3.28	3.31
Italy	9.57	9.31	9.05
Lithuania	1.64	1.72	1.72
Luxembourg	0.80	0.82	0.82
Malta	1.19	1.24	1.24
Netherlands	7.72	7.70	7.87
Norway	3.02	3.11	3.13
Poland	4.75	4.40	4.46
Portugal	3.47	3.15	3.12
Romania	4.84	5.08	5.11
Sweden	4.35	4.41	4.50
Slovak Republic	2.02	1.97	2.01
United Kingdom	7.92	8.31	8.37

Table C4: Descriptive statistics of the sample - Correspondence with samples from Figure 4, Table A4 and Table B4.

	Luxembourg	Finland	Norway	Sweden	Denmark	France	Netherlands	Malta	Cyprus
	Mean / Percentage *(SD)*	Mean / Percentage *(SD)*	Mean / Percentage *(SD)*	Mean / Percentage *(SD)*	Mean / Percentage *(SD)*	Mean / Percentage *(SD)*	Mean / Percentage *(SD)*	Mean / Percentage *(SD)*	Mean / Percentage *(SD)*
**Age**									
15 to 24	5.33	8.07	9.21	5.93	16.77	5.66	10.64	13.68	5.88
25 to 34	22.77	21.32	21.23	19.79	16.75	18.52	17.28	22.89	24.09
35 to 44	28.03	23.68	23.48	25.01	20.39	27.52	19.09	24.94	28.30
45 to 54	31.82	25.73	26.52	27.34	25.40	31.15	28.19	21.50	25.32
55 to 64	12.05	21.20	19.56	21.93	20.69	17.16	24.81	16.98	16.41
**Gender**									
Man	52.02	47.80	51.03	49.23	48.05	44.06	50.56	56.92	47.53
Woman	47.98	52.20	48.97	50.77	51.95	55.94	49.44	43.08	52.47
**Educational level**	4.21	4.37	4.22	4.39	4.05	3.94	4.14	3.50	4.18
	(2.10)	*(1.77)*	*(1.77)*	*(1.75)*	*(1.87)*	*(1.74)*	*(1.91)*	*(1.74)*	*(1.87)*
**Working time**									
Full-time	80.68	85.28	80.72	88.77	74.18	81.69	63.25	83.82	90.60
Part-time	16.82	11.21	14.13	9.45	12.70	16.25	27.31	14.70	8.16
Marginal work	2.51 (!)	3.52	5.14	1.78	13.12	2.06	9.44	1.48 (!)	1.24
**Nationality**									
Native	59.96	96.85	91.83	96.36	95.67	95.99	97.63	98.12	78.60
EU/EFTA	36.87	1.83	6.02	2.22	2.30	1.76	1.49	1.88 (j)	12.65
Non-EU/EFTA	3.17	1.33	2.14	1.42	2.03	2.24	0.88	-	8.75
**Work contract**									
Permanent	95.37	89.09	97.16	94.26	94.01	90.08	94.49	97.06	84.75
6 months or less	na	4.95	0.43	2.15	1.25	5.60	1.07	0.89 (!)	3.09
Between 7 and 12 months	1.22 (!)	3.82	0.67	1.26	1.47	2.75	3.65	1.11 (!)	7.01
More than one year	2.33	2.14	1.74	2.32	3.28	1.57	0.79	0.94 (!)	5.16
**Supervisory role**									
No	66.82	80.64	62.72	65.72	83.95	80.41	76.53	63.52	80.45
Yes	33.18	19.36	37.28	34.28	16.05	19.59	23.47	36.48	19.55
**Occupation**									
Managers	403.76 (k)	3.03	9.20	6.69	2.65	7.30	5.98	444.63 (k)	4.16
	(236.01)							*(243.49)*	
Professionals		26.02	28.32	31.18	28.46	16.97	29.31		17.13
Technicians and associate professionals	-	20.36	18.64	20.46	18.01	22.92	18.28	-	13.99
Clerical support workers	-	6.98	6.00	6.66	7.88	11.12	10.97	-	14.39
Service and sales workers	-	19.01	19.14	16.81	20.96	14.28	16.52	-	18.74
Skilled agricultural, forestry and fisheries	-	1.04	0.80	0.66	0.78	1.78	0.94	-	0.64
Craft and related trades workers	-	9.74	8.97	8.60	6.14	7.07	6.94	-	9.77
Plant and machine operators, and assemblers	-	7.53	5.85	5.64	4.31	7.59	4.20	-	4.43
Elementary	-	6.30	3.10	3.31	10.81	10.97	6.86	-	16.73

Note: (i) Indicates that the categories "Part-time" and "Marginal work" are included under the same category. **(j)** Indicates that the categories "EU/EFTA" and "Non-EU/EFTA" were included under the same category. **(k)** Indicates that Occupation was included as a continuous (instead of categorical) variable. **(!)** Indicates that the coefficient is unreliable because of few observations, according to Eurostat guidelines. **na** Refers to frequencies that are not shown because of the low number of observations.

(continued)

	Ireland	Austria	Italy	Portugal	Czechia	Spain	Belgium	Germany	Greece
	Mean / Percentage *(SD)*	Mean / Percentage *(SD)*	Mean / Percentage *(SD)*	Mean / Percentage *(SD)*	Mean / Percentage *(SD)*	Mean / Percentage *(SD)*	Mean / Percentage *(SD)*	Mean / Percentage *(SD)*	Mean / Percentage *(SD)*
**Age**									
15 to 24	8.46	8.83	3.40	4.94	6.00	3.68	5.52	5.59	4.55
25 to 34	24.13	21.39	14.72	15.87	19.50	16.06	23.51	19.53	20.07
35 to 44	30.15	23.63	27.54	30.72	27.82	30.43	26.53	20.84	31.27
45 to 54	22.99	31.24	33.07	29.76	27.65	30.59	27.92	31.01	31.38
55 to 64	14.27	14.91	21.26	18.70	19.03	19.23	16.52	23.02	12.74
**Gender**									
Man	47.65	50.66	51.99	46.76	50.92	50.40	49.46	50.60	53.02
Woman	52.35	49.34	48.01	53.24	49.08	49.60	50.54	49.40	46.98
**Educational level**	4.54	3.79	3.46	3.23	3.78	4.09	4.31	4.01	4.04
	(1.68)	*(1.54)*	*(1.82)*	*(2.23)*	*(1.62)*	*(2.03)*	*(1.98)*	*(1.66)*	*(1.82)*
**Working time**									
Full-time	75.21	73.30	74.20	92.73	95.10	83.33	76.01	72.02	80.88
Part-time	21.40	21.60	23.44	5.69	4.44	13.59	22.15	19.97	17.72
Marginal work	3.39	5.10	2.35	1.58	0.46	3.08	1.84	8.01	1.40
**Nationality**									
Native	87.46	85.83	88.59	98.08	98.25	94.78	90.03	92.01	91.74
EU/EFTA	9.88	8.40	3.86	0.58	1.11	2.19	7.56	3.92	1.46
Non-EU/EFTA	2.65	5.77	7.54	1.34	0.64	3.03	2.41	4.08	6.80
**Work contract**									
Permanent	98.30	99.41	88.84	84.05	92.38	86.42	92.71	97.74	89.27
6 months or less	0.49	0.18 (!)	6.01	7.08	1.22	8.69	3.71	0.52	4.65
Between 7 and 12 months	0.45	0.27 (!)	4.50	7.40	2.95	3.59	2.37	1.11	4.49
More than one year	0.75	na	0.65	1.46	3.45	1.31	1.21	0.64	1.59
**Supervisory role**									
No	69.53	74.38	79.56	72.44	82.55	81.26	78.60	75.17	88.23
Yes	30.47	25.62	20.44	27.56	17.45	18.74	21.40	24.83	11.77
**Occupation**									
Managers	7.14	4.86	1.33	3.16	3.87	2.78	6.27	4.25	1.38
Professionals	23.63	15.58	14.07	18.22	13.98	19.37	23.64	17.20	20.82
Technicians and associate professionals	13.65	20.32	17.61	13.44	18.29	12.04	15.24	23.11	9.75
Clerical support workers	11.62	11.00	15.45	9.27	10.91	12.57	13.21	14.37	15.28
Service and sales workers	20.79	18.75	17.42	19.18	14.30	20.41	12.74	13.55	23.38
Skilled agricultural, forestry and fisheries	0.84	0.68	0.85	1.97	0.79	1.14	0.50	0.71	0.70
Craft and related trades workers	8.51	13.18	11.60	11.81	15.08	9.76	9.37	12.40	9.00
Plant and machine operators, and assemblers	5.44	6.22	8.23	9.04	16.63	8.59	7.53	6.46	7.47
Elementary	8.38	9.41	13.44	13.91	6.15	13.35	11.49	7.96	12.22

Note: (i) Indicates that the categories "Part-time" and "Marginal work" are included under the same category. **(j)** Indicates that the categories "EU/EFTA" and "Non-EU/EFTA" were included under the same category. **(k)** Indicates that Occupation was included as a continuous (instead of categorical) variable. **(!)** Indicates that the coefficient is unreliable because of few observations, according to Eurostat guidelines. **na** Refers to frequencies that are not shown because of the low number of observations.

(continued)

	Poland	UK	Switzerland	Slovakia	Hungary	Bulgaria	Romania
	Mean / Percentage *(SD)*	Mean / Percentage *(SD)*	Mean / Percentage *(SD)*	Mean / Percentage *(SD)*	Mean / Percentage *(SD)*	Mean / Percentage *(SD)*	Mean / Percentage *(SD)*
**Age**							
15 to 24	4.89	10.46	6.86	6.42	7.14	4.12	4.59
25 to 34	23.97	22.67	18.00	20.32	19.77	17.94	20.73
35 to 44	28.42	23.92	25.38	27.23	28.44	27.19	30.16
45 to 54	23.71	25.71	29.94	26.32	26.88	27.71	30.98
55 to 64	19.02	17.24	19.82	19.71	17.76	23.04	13.55
**Gender**							
Man	47.33	48.21	49.84	49.37	52.79	51.22	53.92
Woman	52.67	51.79	50.16	50.63	47.21	48.78	46.08
**Educational level**	4.47	4.04	4.43	3.87	3.58	3.97	3.84
	(1.91)	*(1.75)*	*(1.97)*	*(1.70)*	*(1.48)*	*(1.83)*	*(1.67)*
**Working time**							
Full-time	94.12	74.84	72.74	93.74	94.74	97.75	98.90
Part-time	5.33	20.88	19.02	5.53	4.89	2.25 (i)	1.10 (i)
Marginal work	0.54	4.28	8.24	0.74	0.37	-	-
**Nationality**							
Native	99.71	90.29	64.68	-	99.68	-	-
EU/EFTA	0.29 (j)	6.62	28.52	-	0.19	-	-
Non-EU/EFTA	-	3.09	6.80	-	0.13	-	-
**Work contract**							
Permanent	82.48	99.23	98.51	92.51	89.31	96.82	99.57
6 months or less	3.39	0.23	0.73	3.93	3.58	2.14	0.17 (!)
Between 7 and 12 months	5.82	0.21	0.61	2.71	6.51	0.87	0.18
More than one year	8.30	0.33	na	0.85	0.60	na	na
**Supervisory role**							
No	80.93	62.72	64.18	88.86	87.15	89.26	92.61
Yes	19.07	37.28	35.82	11.14	12.85	10.74	7.39
**Occupation**							
Managers	6.32	11.08	10.07	3.18	3.72	3.39	1.62
Professionals	23.12	24.85	24.77	12.06	11.07	15.85	19.41
Technicians and associate professionals	15.24	13.45	21.53	15.42	13.87	9.52	7.84
Clerical support workers	7.68	11.14	8.43	10.81	7.58	6.76	6.16
Service and sales workers	13.58	19.56	16.20	18.39	13.64	21.04	19.25
Skilled agricultural, forestry and fisheries	0.37	0.45	0.98	0.61	2.17	1.43	0.75
Craft and related trades workers	14.76	6.28	9.86	12.86	15.45	14.44	20.89
Plant and machine operators, and assemblers	11.10	4.77	3.79	16.92	18.19	15.62	16.05
Elementary	7.83	8.42	4.37	9.75	14.32	11.94	8.02

Note: (i) Indicates that the categories "Part-time" and "Marginal work" are included under the same category. **(j)** Indicates that the categories "EU/EFTA" and "Non-EU/EFTA" were included under the same category. **(k)** Indicates that Occupation was included as a continuous (instead of categorical) variable. **(!)** Indicates that the coefficient is unreliable because of few observations, according to Eurostat guidelines. **na** Refers to frequencies that are not shown because of the low number of observations.

Table C5: Descriptive statistics of the sample - Correspondence with samples from Figure 5, Table A5 and Table B5.

	Ireland	Finland	Sweden	Switzerland	Portugal	UK	Greece	Spain	Italy
	Mean / Percentage *(SD)*	Mean / Percentage *(SD)*	Mean / Percentage *(SD)*	Mean / Percentage *(SD)*	Mean / Percentage *(SD)*	Mean / Percentage *(SD)*	Mean / Percentage *(SD)*	Mean / Percentage *(SD)*	Mean / Percentage *(SD)*
**Age**									
15 to 24	8.58	7.20	5.16	13.69	3.58	10.52	4.55	2.42	3.77
25 to 34	24.10	20.10	19.34	17.30	13.93	22.68	19.67	14.32	14.21
35 to 44	30.07	23.80	25.42	23.30	31.39	23.88	31.25	30.68	26.89
45 to 54	23.05	26.76	27.89	27.49	31.01	25.70	31.64	31.97	33.29
55 to 64	14.20	22.14	22.19	18.22	20.09	17.22	12.89	20.62	21.84
**Gender**									
Man	47.73	49.44	50.00	50.13	46.64	48.22	53.83	50.81	52.42
Woman	52.27	50.56	50.00	49.87	53.36	51.78	46.17	49.19	47.58
**Educational level**	4.54	4.35	4.40	4.28	3.24	4.03	4.11	4.15	3.48
	(1.68)	*(1.76)*	*(1.75)*	*(2.00)*	*(2.25)*	*(1.75)*	*(1.81)*	*(2.04)*	*(1.81)*
**Working time**									
Full-time	75.84	86.20	89.91	74.54	94.13	74.94	81.31	85.57	75.79
Part-time	20.80	10.59	8.76	17.87	4.54	20.80	17.52	12.14	22.35
Marginal work	3.36	3.21	1.34	7.59	1.34	4.26	1.17	2.28	1.86
**Nationality**									
Native	87.36	96.87	96.77	65.88	98.44	90.30	92.73	95.31	89.19
EU/EFTA	9.93	1.86	2.16	27.28	0.50	6.61	1.31	2.01	3.66
Non-EU/EFTA	2.71	1.26	1.07	6.85	1.07	3.09	5.96	2.68	7.15
**Work contract**									
Permanent	99.36	98.92	97.99	90.98	97.32	99.56	97.85	98.31	96.16
6 months or less	na	0.56	1.49	0.74	1.31	0.14	0.89	0.85	1.71
Between 7 and 12 months	0.27	na	0.33	0.97	1.20	na	0.56	0.47	0.67
More than one year	0.22	0.34	na	7.31	na	0.21	0.69	0.37	1.46
**Supervisory role**									
No	69.27	79.30	64.81	66.88	70.62	62.65	87.22	79.23	78.67
Yes	30.73	20.70	35.19	33.12	29.38	37.35	12.78	20.77	21.33
**Occupation**									
Managers	7.17	3.30	6.91	9.32	3.52	11.11	1.52	3.09	1.37
Professionals	23.74	25.81	31.25	24.25	19.03	24.86	22.01	20.01	13.92
Technicians and associate professionals	13.75	20.42	20.98	21.25	14.24	13.48	10.21	12.72	18.48
Clerical support workers	11.49	6.86	6.73	8.38	9.48	11.08	15.80	13.16	16.04
Service and sales workers	20.79	18.37	15.85	16.86	18.32	19.56	23.00	19.54	17.05
Skilled agricultural, forestry and fisheries	0.77	0.97	0.54	1.19	1.66	0.45	0.68	1.13	0.62
Craft and related trades workers	8.57	10.37	8.94	11.22	12.12	6.30	9.07	9.79	12.16
Plant and machine operators, and assemblers	5.47	7.89	5.76	3.56	8.99	4.76	7.40	8.40	8.19
Elementary	8.26	6.01	3.05	3.98	12.66	8.40	10.31	12.17	12.17

Note: (i) Indicates that the categories "Part-time" and "Marginal work" are included under the same category. **(j)** Indicates that the categories "EU/EFTA" and "Non-EU/EFTA" were included under the same category. **(!)** Indicates that the coefficient is unreliable because of few observations, according to Eurostat guidelines. **na** Refers to frequencies that are not shown because of the low number of observations.

(continued)

	Germany	Denmark	France	Poland	Austria
	Mean / Percentage *(SD)*	Mean / Percentage *(SD)*	Mean / Percentage *(SD)*	Mean / Percentage *(SD)*	Mean / Percentage *(SD)*
**Age**					
15 to 24	9.60	16.69	6.50	4.20	12.02
25 to 34	19.44	17.08	17.46	22.67	21.02
35 to 44	19.79	20.31	27.08	29.01	22.81
45 to 54	29.38	25.37	31.65	24.56	29.89
55 to 64	21.79	20.55	17.31	19.57	14.27
**Gender**					
Man	51.00	48.74	44.77	47.36	50.79
Woman	49.00	51.26	55.23	52.64	49.21
**Educational level**	3.94	4.05	3.95	4.57	3.75
	(1.66)	*(1.85)*	*(1.73)*	*(1.92)*	*(1.55)*
**Working time**					
Full-time	73.51	76.59	83.55	94.63	74.25
Part-time	18.88	12.15	14.68	5.37 (i)	20.90
Marginal work	7.61	11.26	1.77	-	4.85
**Nationality**					
Native	92.00	95.71	96.53	99.75	85.95
EU/EFTA	3.83	2.28	1.64	0.25 (j)	8.27
Non-EU/EFTA	4.17	2.00	1.84	-	5.78
**Work contract**					
Permanent	92.87	95.55	96.28	93.71	95.04
6 months or less	0.88	0.71	0.76	3.00	0.78
Between 7 and 12 months	1.14	0.63	na	1.73	0.54
More than one year	5.11	3.11	2.29	1.55	3.64
**Supervisory role**					
No	76.21	83.79	79.58	79.48	75.40
Yes	23.79	16.21	20.42	20.52	24.60
**Occupation**					
Managers	4.07	2.69	7.64	6.88	4.66
Professionals	16.61	28.40	17.66	25.05	15.34
Technicians and associate professionals	23.66	18.20	22.98	16.32	20.34
Clerical support workers	14.27	7.86	11.30	7.95	10.85
Service and sales workers	13.81	20.46	13.97	12.42	19.14
Skilled agricultural, forestry and fisheries	0.74	0.94	1.75	0.31	0.71
Craft and related trades workers	12.98	7.24	7.60	14.14	13.91
Plant and machine operators, and assemblers	6.42	4.34	7.13	10.53	6.02
Elementary	7.45	9.88	9.98	6.41	9.03

Note: (i) Indicates that the categories "Part-time" and "Marginal work" are included under the same category. **(j)** Indicates that the categories "EU/EFTA" and "Non-EU/EFTA" were included under the same category. **(!)** Indicates that the coefficient is unreliable because of few observations, according to Eurostat guidelines. **na** Refers to frequencies that are not shown because of the low number of observations.

Table C6: Descriptive statistics of the sample - Correspondence with samples from Figure 6, Table A6 and Table B6.

	**Denmark**	**Sweden**	**Italy**	**Norway**	**Finland**	**Czechia**	**Austria**	**Slovakia**	**Spain**
	Mean / Percentage *(SD)*	Mean / Percentage *(SD)*	Mean / Percentage *(SD)*	Mean / Percentage *(SD)*	Mean / Percentage *(SD)*	Mean / Percentage *(SD)*	Mean / Percentage *(SD)*	Mean / Percentage *(SD)*	Mean / Percentage *(SD)*
**Age**									
15 to 24	16.21	6.28	2.39	9.25	8.71	5.51	9.11	5.95	2.07
25 to 34	16.55	19.04	13.35	20.67	20.06	18.73	21.63	19.88	13.73
35 to 44	20.43	25.01	27.46	23.37	23.35	27.76	23.62	27.06	30.98
45 to 54	25.72	27.45	34.21	26.82	26.14	27.97	30.87	26.38	32.34
55 to 64	21.10	22.22	22.59	19.89	21.73	20.04	14.77	20.73	20.89
**Gender**									
Man	48.07	49.49	52.15	51.41	49.14	51.35	50.55	49.56	50.80
Woman	51.93	50.51	47.85	48.59	50.86	48.65	49.45	50.44	49.20
**Educational level**	4.06	4.39	3.48	4.20	4.34	3.80	3.80	3.93	4.13
	(1.85)	*(1.75)*	(1.82)	*(1.76)*	*(1.76)*	*(1.63)*	*(1.55)*	*(1.71)*	*(2.04)*
**Working time**									
Full-time	74.72	88.31	75.80	80.70	84.97	94.34	73.07	96.30	85.51
Part-time	12.97	9.35	22.27	14.02	11.09	4.92	21.68	3.24	12.12
Marginal work	12.32	2.33	1.92	5.29	3.94	0.74	5.25	0.46	2.37
**Nationality**									
Native	95.82	96.73	89.27	92.08	96.92	98.35	85.60	-	95.29
EU/EFTA	2.28	2.15	3.65	5.92	1.83	1.06	8.61	-	2.05
Non-EU/EFTA	1.90	1.12	7.07	2.00	1.24	0.60	5.79	-	2.66
**Work contract**									
Permanent	96.19	96.50	99.52	99.17	96.27	97.86	96.49	98.14	99.43
6 months or less	1.07	1.79	0.25	0.30	2.39	0.35	1.30	0.78	0.40
Between 7 and 12 months	0.78	0.51	0.18	na	0.78	0.98	1.37	0.71	0.10
More than one year	1.96	1.19	na	0.32	0.56	0.81	0.84	0.37	na
**Supervisory role**									
No	83.63	65.16	78.16	62.32	79.71	81.92	74.44	88.28	79.09
Yes	16.37	34.84	21.84	37.68	20.29	18.08	25.56	11.72	20.91
**Occupation**									
Managers	2.71	6.82	1.42	9.40	3.23	4.16	4.82	3.36	3.12
Professionals	28.67	31.03	14.09	27.88	25.52	14.37	16.05	12.81	19.70
Technicians and associate professionals	18.26	20.83	18.69	18.75	20.42	18.64	20.04	16.14	12.69
Clerical support workers	7.81	6.70	16.18	5.98	6.85	10.83	10.89	11.14	13.13
Service and sales workers	20.97	16.51	16.69	19.13	18.77	14.28	18.84	18.91	19.68
Skilled agricultural, forestry and fisheries	0.79	0.61	0.62	0.81	1.07	0.80	0.74	0.63	1.12
Craft and related trades workers	6.26	8.64	11.86	9.05	10.09	15.14	13.06	13.42	9.79
Plant and machine operators, and assemblers	4.37	5.59	8.20	5.91	7.72	16.10	6.19	17.06	8.45
Elementary	10.17	3.27	12.27	3.11	6.33	5.68	9.38	6.53	12.32

Note: (j) Indicates that the categories "EU/EFTA" and "Non-EU/EFTA" were included under the same category. **(!)** Indicates that the coefficient is unreliable because of few observations, according to Eurostat guidelines.**na** Refers to frequencies that are not shown because of the low number of observations.

(continued)

	France	Portugal	UK	Belgium	Poland	Netherlands	Hungary
	Mean / Percentage *(SD)*	Mean / Percentage *(SD)*	Mean / Percentage *(SD)*	Mean / Percentage *(SD)*	Mean / Percentage *(SD)*	Mean / Percentage *(SD)*	Mean / Percentage *(SD)*
**Age**							
15 to 24	5.16	2.92	10.53	5.25	3.64	11.02	6.85
25 to 34	17.78	13.47	22.63	22.47	22.32	16.41	19.72
35 to 44	27.27	31.75	23.86	26.49	28.84	19.06	28.86
45 to 54	31.96	31.43	25.72	28.56	24.61	28.35	26.90
55 to 64	17.82	20.42	17.26	17.23	20.59	25.16	17.67
**Gender**							
Man	44.56	46.65	48.14	49.67	47.13	50.88	53.21
Woman	55.44	53.35	51.86	50.33	52.87	49.12	46.79
**Educational level**	3.96	3.23	4.04	4.33	4.58	4.13	3.70
	(1.73)	*(2.25)*	*(1.75)*	*(1.98)*	*(1.92)*	*(1.91)*	*(1.49)*
**Working time**							
Full-time	82.89	93.73	74.70	76.45	93.59	62.89	95.14
Part-time	14.92	4.78	20.92	21.62	5.68	27.10	4.55
Marginal work	2.19	1.49	4.38	1.93	0.74	10.00	0.31
**Nationality**							
Native	96.56	98.44	90.31	90.61	99.69	97.77	99.70
EU/EFTA	1.68	0.52	6.60	7.30	0.31 (j)	1.42	0.16
Non-EU/EFTA	1.76	1.04	3.09	2.08	-	0.81	0.14
**Work contract**							
Permanent	95.93	98.98	99.42	98.05	93.09	98.20	99.09
6 months or less	2.75	0.63	0.25	1.20	1.38	0.50	0.33
Between 7 and 12 months	0.60	0.28	0.14	0.36	2.26	1.10	0.42
More than one year	0.71	na	0.19	0.39	3.27	0.20	0.16
**Supervisory role**							
No	79.58	70.35	62.66	77.75	79.00	76.05	86.06
Yes	20.42	29.65	37.34	22.25	21.00	23.95	13.94
**Occupation**							
Managers	7.75	3.58	11.10	6.52	6.98	6.17	4.17
Professionals	17.67	18.72	24.82	23.52	25.47	29.33	12.20
Technicians and associate professionals	23.03	14.30	13.46	15.66	16.17	18.27	15.03
Clerical support workers	11.06	9.44	11.10	13.40	7.56	10.76	7.81
Service and sales workers	13.94	18.50	19.63	12.54	12.68	16.81	14.37
Skilled agricultural, forestry and fisheries	1.68	1.64	0.45	0.46	0.34	0.94	2.00
Craft and related trades workers	7.13	12.02	6.27	9.35	13.83	6.90	16.06
Plant and machine operators, and assemblers	7.53	9.04	4.75	7.43	10.39	3.96	19.65
Elementary	10.23	12.77	8.42	11.11	6.59	6.86	8.70

Note: (j) Indicates that the categories "EU/EFTA" and "Non-EU/EFTA" were included under the same category. **(!)** Indicates that the coefficient is unreliable because of few observations, according to Eurostat guidelines.**na** Refers to frequencies that are not shown because of the low number of observations.

Table C7: Descriptive statistics of the sample - Correspondence with samples from Table B7

	Finland	Luxembourg	Cyprus	Malta	Denmark	Estonia	France	Austria	Netherlands
	Mean / Percentage *(SD)*	Mean / Percentage *(SD)*	Mean / Percentage *(SD)*	Mean / Percentage *(SD)*	Mean / Percentage *(SD)*	Mean / Percentage *(SD)*	Mean / Percentage *(SD)*	Mean / Percentage *(SD)*	Mean / Percentage *(SD)*
**Age**									
15 to 24	9.41	6.02	6.38	14.41	18.90	7.87	9.04	12.31	12.78
25 to 34	21.65	24.06	24.01	22.77	17.02	19.55	18.52	20.58	18.35
35 to 44	23.42	28.33	28.29	24.71	19.75	23.97	26.28	22.66	18.69
45 to 54	25.12	30.15	24.82	21.33	24.41	26.79	29.68	30.12	26.91
55 to 64	20.41	11.44	16.51	16.77	19.93	21.82	16.48	14.34	23.27
**Gender**									
Man	47.86	51.80	47.09	56.65	48.16	46.34	44.99	51.53	50.78
Woman	52.14	48.20	52.91	43.35	51.84	53.66	55.01	48.47	49.22
**Educational level**	4.37	4.22	4.14	3.50	4.03	4.26	3.92	3.75	4.11
	(1.77)	*(2.12)*	*(1.87)*	*(1.74)*	*(1.87)*	*(1.80)*	*(1.72)*	*(1.56)*	*(1.90)*
**Working time**									
Full-time	85.29	81.24	90.46	82.98	73.93	91.65	81.22	74.30	62.53
Part-time	11.00	16.07	8.28	15.24	12.79	6.95	16.38	20.86	27.93
Marginal work	3.71	2.69 (!)	1.26	1.78 (!)	13.28	1.41	2.40	4.85	9.54
**Nationality**									
Native	97.06	57.58	78.37	98.06	95.79	87.98	96.07	85.64	97.62
EU/EFTA	1.78	38.94	12.76	1.94 (j)	2.26	0.62 (!)	1.71	8.49	1.43
Non-EU/EFTA	1.15	3.48	8.87	-	1.95	11.40	2.22	5.86	0.95
**Work contract**									
Permanent	85.80	93.11	83.69	94.11	87.65	97.81	83.66	91.33	84.84
Involuntary temporary	10.33	4.79	15.30	2.86	5.29	0.46	9.21	0.54	6.56
Instrumental temporary	0.86	1.54 (!)	0.62	1.92	3.83	1.44	3.58	4.86	6.05
Voluntary temporary	3.01	na	na	1.12	3.23	na	3.54	3.27	2.55
**Supervisory role**									
No	80.80	67.08	80.83	63.77	84.54	74.19	81.55	75.48	77.71
Yes	19.20	32.92	19.17	36.23	15.46	25.81	18.45	24.52	22.29
**Occupation**									
Managers	3.00	406.77 (k)	3.95	443.86 (k)	2.53	9.23	6.90	4.65	5.61
		(237.86)		*(243.04)*					
Professionals	26.06		16.90		28.27	19.57	16.61	15.61	28.52
Technicians and associate professionals	20.73	-	13.49	-	17.32	14.07	22.48	19.98	17.98
Clerical support workers	7.12	-	14.41	-	7.87	6.13	11.04	10.61	10.98
Service and sales workers	19.00	-	18.83	-	21.64	13.48	14.45	19.22	17.21
Skilled agricultural, forestry and fisheries	1.04	-	0.64	-	0.79	0.94 (!)	2.16	0.75	0.93
Craft and related trades workers	9.57	-	9.99	-	6.88	12.18	7.62	13.95	7.23
Plant and machine operators, and assemblers	7.37	-	4.53	-	4.18	14.80	7.73	6.08	4.40
Elementary	13.47	-	21.80	-	14.70	24.40	18.74	15.25	11.55
**Tenure (in months)**	118.59	121.81	112.85	117.88	87.32	97.83	138.72	123.26	135.65
	(125.07)	*(114.15)*	*(113.7)*	*(126.15)*	*(107.62)*	*(101.55)*	*(127.96)*	*(125.74)*	*(133.37)*
**Income (deciles)**	5.43	5.43	4.85	5.45	5.41	5.34	-	5.55	5.90
	(2.85)	*(2.84)*	*(2.78)*	*(2.87)*	*(2.94)*	*(2.76)*	-	*(2.82)*	*(2.81)*
**Number of children in the household**	-	-	0.87	0.73	-	0.88	0.99	0.64	0.92
	-	-	*(1.03)*	*(0.90)*	-	*(1.08)*	*(1.07)*	*(0.91)*	*(1.07)*
**Unemployed adults in the household**									
No	-	-	89.87	96.55	-	95.14	95.48	96.38	96.89
Yes	-	-	10.13	3.45	-	4.86	4.52	3.62	3.11

Note: (i) Indicates that the categories "Part-time" and "Marginal work" are included under the same category. **(j)** Indicates that the categories "EU/EFTA" and "Non-EU/EFTA" were included under the same category. **(k)** Indicates that Occupation was included as a continuous (instead of categorical) variable. **(!)** Indicates that the coefficient is unreliable because of few observations, according to Eurostat guidelines. **na** Refers to frequencies that are not shown because of the low number of observations.

(continued)

	Italy	Czechia	Greece	Portugal	Belgium	Spain	Poland	Germany	UK
	Mean / Percentage *(SD)*	Mean / Percentage *(SD)*	Mean / Percentage *(SD)*	Mean / Percentage *(SD)*	Mean / Percentage *(SD)*	Mean / Percentage *(SD)*	Mean / Percentage *(SD)*	Mean / Percentage *(SD)*	Mean / Percentage *(SD)*
**Age**									
15 to 24	4.84	6.52	5.05	6.20	6.53	5.14	6.86	9.70	11.04
25 to 34	15.63	19.46	20.34	16.24	23.47	17.55	24.78	19.78	22.60
35 to 44	26.95	27.51	31.02	30.31	26.15	29.89	27.47	19.89	23.74
45 to 54	32.10	27.21	30.97	29.31	27.49	29.46	22.47	29.18	25.44
55 to 64	20.48	19.30	12.62	17.94	16.35	17.96	18.42	21.45	17.18
**Gender**									
Man	52.14	50.58	52.83	45.95	49.51	50.57	47.26	50.69	48.08
Woman	47.86	49.42	47.17	54.05	50.49	49.43	52.74	49.31	51.92
**Educational level**	3.46	3.79	4.03	3.24	4.30	4.03	4.43	3.93	4.04
	(1.81)	*(1.62)*	*(1.82)*	*(2.22)*	*(1.99)*	*(2.03)*	*(1.90)*	*(1.66)*	*(1.75)*
**Working time**									
Full-time	73.78	93.75	80.68	92.27	75.41	82.09	92.59	72.97	74.14
Part-time	23.71	5.42	17.86	6.05	22.25	14.34	6.46	19.24	21.22
Marginal work	2.51	0.83	1.46	1.68	2.34	3.57	0.95	7.78	4.65
**Nationality**									
Native	88.55	98.25	91.60	98.01	89.85	94.33	99.61	91.77	90.15
EU/EFTA	3.89	1.13	1.49	0.57	7.63	2.29	0.39 (j)	3.93	6.69
Non-EU/EFTA	7.56	0.63	6.91	1.42	2.52	3.38		4.31	3.16
**Work contract**									
Permanent	84.53	90.47	87.09	80.05	90.60	75.63	73.87	90.66	96.85
Involuntary temporary	11.52	7.47	10.47	16.70	7.13	22.12	15.69	2.10	1.41
Instrumental temporary	3.51	na	1.91	2.31	0.47	1.49	4.96	6.93	0.56
Voluntary temporary	0.44	1.98	0.53	0.95	1.80	0.76	5.48	0.31	1.19
**Supervisory role**									
No	80.18	82.77	88.36	72.64	78.94	82.97	81.80	76.68	63.36
Yes	19.82	17.23	11.64	27.36	21.06	17.03	18.20	23.32	36.64
**Occupation**									
Managers	1.29	3.89	1.38	3.04	6.17	2.49	5.92	3.90	10.88
Professionals	13.78	14.03	20.84	18.38	23.61	18.85	22.59	16.58	24.75
Technicians and associate professionals	17.26	18.18	9.62	13.22	15.09	11.54	14.94	23.43	13.39
Clerical support workers	15.25	10.87	15.16	9.27	13.10	12.01	7.89	14.22	11.16
Service and sales workers	17.99	14.52	23.54	19.54	13.11	20.45	14.35	13.97	19.72
Skilled agricultural, forestry and fisheries	0.88	0.80	0.74	1.63	0.50	1.13	0.36	0.74	0.44
Craft and related trades workers	11.80	14.91	9.02	11.77	9.39	10.47	14.81	12.84	6.22
Plant and machine operators, and assemblers	8.21	16.42	7.36	9.01	7.48	8.46	10.81	6.53	4.78
Elementary	21.75	22.80	19.71	23.15	19.04	23.06	19.14	14.31	13.43
**Tenure (in months)**	152.72	122.05	129.16	148.76	135.53	137.94	123.60	136.29	99.80
	(130.71)	*(114.99)*	*(115.01)*	*(130.53)*	*(126.88)*	*(130.1)*	*(122.94)*	*(131.17)*	*(104.74)*
**Income (deciles)**	5.48	-	-	5.46	5.55	5.60	-	5.61	-
	(2.85)	-	-	*(2.86)*	*(2.86)*	*(2.87)*	-	*(2.85)*	-
**Number of children in the household**	0.79	0.72	0.80	0.83	0.89	0.83	0.83	0.58	0.77
	(0.93)	*(0.94)*	*(0.95)*	*(0.88)*	*(1.07)*	*(0.93)*	*(0.96)*	*(0.90)*	*(1.01)*
**Unemployed adults in the household**									
No	92.09	97.23	83.68	92.37	95.68	86.37	96.00	98.18	96.81
Yes	7.91	2.77	16.32	7.63	4.32	13.63	4.00	1.82	3.19

Note: (i) Indicates that the categories "Part-time" and "Marginal work" are included under the same category. **(j)** Indicates that the categories "EU/EFTA" and "Non-EU/EFTA" were included under the same category. **(k)** Indicates that Occupation was included as a continuous (instead of categorical) variable. **(!)** Indicates that the coefficient is unreliable because of few observations, according to Eurostat guidelines. **na** Refers to frequencies that are not shown because of the low number of observations.

(continued)

	Switzerland	Ireland	Hungary	Bulgaria	Romania	Lithuania	Slovakia
	Mean / Percentage *(SD)*	Mean / Percentage *(SD)*	Mean / Percentage *(SD)*	Mean / Percentage *(SD)*	Mean / Percentage *(SD)*	Mean / Percentage *(SD)*	Mean / Percentage *(SD)*
**Age**							
15 to 24	14.29	9.96	7.56	4.45	4.62	5.55	6.74
25 to 34	17.60	23.92	19.83	17.71	20.74	15.94	20.17
35 to 44	23.44	29.52	28.39	26.99	30.15	20.63	26.86
45 to 54	26.76	22.55	26.61	27.70	30.94	31.86	26.03
55 to 64	17.91	14.05	17.62	23.15	13.55	26.02	20.21
**Gender**							
Man	49.95	47.65	52.78	51.40	53.92	43.79	49.30
Woman	50.05	52.35	47.22	48.60	46.08	56.21	50.70
**Educational level**	4.27	4.52	3.58	3.88	3.84	4.58	3.86
	(1.99)	*(1.68)*	*(1.48)*	*(1.81)*	*(1.67)*	*(1.61)*	*(1.70)*
**Working time**							
Full-time	74.31	73.63	94.50	97.52	98.89	91.36	92.68
Part-time	18.02	22.42	5.10	2.48 (i)	1.11 (i)	7.49	6.27
Marginal work	7.67	3.96	0.40	-	-	1.16	1.05
**Nationality**							
Native	65.71	87.57	99.67	-	-	-	-
EU/EFTA	27.51	9.72	0.20	-	-	-	-
Non-EU/EFTA	6.78	2.71	0.13	-	-	-	-
**Work contract**							
Permanent	88.82	94.20	87.53	94.49	99.43	98.17	90.75
Involuntary temporary	1.36	3.52	10.48	3.82	0.43	1.05	7.39
Instrumental temporary	9.15	0.89	1.18	1.00	na	0.66 (!)	na
Voluntary temporary	0.67	1.39	0.81	0.69	na	na	1.72
**Supervisory role**							
No	67.47	70.48	87.25	89.52	92.61	85.93	89.03
Yes	32.53	29.52	12.75	10.48	7.39	14.07	10.97
**Occupation**							
Managers	9.22	6.87	3.71	3.30	1.62	8.06	3.15
Professionals	24.42	23.47	10.99	14.70	19.41	23.62	11.99
Technicians and associate professionals	21.24	13.31	13.78	9.11	7.86	9.25	15.27
Clerical support workers	8.36	11.46	7.56	6.72	6.15	4.96	10.78
Service and sales workers	16.85	21.61	13.70	21.24	19.25	13.36	18.57
Skilled agricultural, forestry and fisheries	1.22	0.88	2.15	1.74	0.74	2.03	0.63
Craft and related trades workers	11.15	8.36	15.38	14.64	20.89	13.98	12.89
Plant and machine operators, and assemblers	3.45	5.44	18.35	15.80	16.04	13.82	16.72
Elementary	7.55	14.05	32.73	28.55	24.07	24.74	26.72
**Tenure (in months)**	98.49	110.80	103.51	106.83	107.03	87.88	121.51
	(105.73)	*(111.21)*	*(110.9)*	*(94.25)*	*(89.35)*	*(83.22)*	*(115.42)*
**Income (deciles)**	5.66	-	4.78	5.23	5.32	5.56	-
	(2.98)	-	*(2.87)*	*(2.85)*	*(2.85)*	*(2.97)*	-
**Number of children in the household**	-	1.09	0.76	0.57	0.59	0.64	0.81
	-	*(1.19)*	*(1.02)*	*(0.82)*	*(0.82)*	*(0.87)*	*(1.02)*
**Unemployed adults in the household**							
No	-	95.51	96.16	95.60	95.72	95.07	92.04
Yes	-	4.49	3.84	4.40	4.28	4.93	7.96

Note: (i) Indicates that the categories "Part-time" and "Marginal work" are included under the same category. **(j)** Indicates that the categories "EU/EFTA" and "Non-EU/EFTA" were included under the same category. **(k)** Indicates that Occupation was included as a continuous (instead of categorical) variable. **(!)** Indicates that the coefficient is unreliable because of few observations, according to Eurostat guidelines. **na** Refers to frequencies that are not shown because of the low number of observations.
